# Intramuscular Artesunate for Severe Malaria in African Children: A Multicenter Randomized Controlled Trial

**DOI:** 10.1371/journal.pmed.1001938

**Published:** 2016-01-12

**Authors:** Peter G. Kremsner, Akim A. Adegnika, Aurore B. Hounkpatin, Jeannot F. Zinsou, Terrie E. Taylor, Yamikani Chimalizeni, Alice Liomba, Maryvonne Kombila, Marielle K. Bouyou-Akotet, Denise P. Mawili Mboumba, Tsiri Agbenyega, Daniel Ansong, Justice Sylverken, Bernhards R. Ogutu, Godfrey A. Otieno, Anne Wangwe, Kalifa A. Bojang, Uduak Okomo, Frank Sanya-Isijola, Charles R. Newton, Patricia Njuguna, Michael Kazungu, Reinhold Kerb, Mirjam Geditz, Matthias Schwab, Thirumalaisamy P. Velavan, Christian Nguetse, Carsten Köhler, Saadou Issifou, Stefanie Bolte, Thomas Engleitner, Benjamin Mordmüller, Sanjeev Krishna

**Affiliations:** 1 Institut für Tropenmedizin, Eberhard Karls Universität Tübingen, Tübingen, Germany; 2 Centre de Recherches Médicales de Lambaréné, Hôpital Albert Schweitzer, Lambaréné, Gabon; 3 Blantyre Malaria Project, University of Malawi College of Medicine, Blantyre, Malawi; 4 Department of Parasitology Mycology, Faculty of Medicine, Université des Sciences de la Santé, Libreville, Gabon; 5 Department of Physiology, University of Science and Technology, School of Medical Sciences, Kumasi, Ghana; 6 Departments of Child Health and Medicine, Komfo Anokye Teaching Hospital, Kumasi, Ghana; 7 Centre for Clinical Research, Kenya Medical Research Institute, Kisumu, Kenya; 8 Medical Research Council Laboratories, Fajara, The Gambia; 9 Centre for Geographic Medicine Research–Coast, Kenya Medical Research Institute, Kilifi, Kenya; 10 Dr. Margarete Fischer-Bosch-Institut für Klinische Pharmakologie, Stuttgart, Germany; 11 Eberhard Karls Universität Tübingen, Tübingen, Germany; 12 Abteilung Klinische Pharmakologie, Universitätsklinikum Tübingen, Tübingen, Germany; 13 Institute for Infection and Immunity, St George’s, University of London, London, United Kingdom; Kenya Medical Research Institute - Wellcome Trust Research Programme, KENYA

## Abstract

**Background:**

Current artesunate (ARS) regimens for severe malaria are complex. Once daily intramuscular (i.m.) injection for 3 d would be simpler and more appropriate for remote health facilities than the current WHO-recommended regimen of five intravenous (i.v.) or i.m. injections over 4 d. We compared both a three-dose i.m. and a three-dose i.v. parenteral ARS regimen with the standard five-dose regimen using a non-inferiority design (with non-inferiority margins of 10%).

**Methods and Findings:**

This randomized controlled trial included children (0.5–10 y) with severe malaria at seven sites in five African countries to assess whether the efficacy of simplified three-dose regimens is non-inferior to a five-dose regimen. We randomly allocated 1,047 children to receive a total dose of 12 mg/kg ARS as either a control regimen of five i.m. injections of 2.4 mg/kg (at 0, 12, 24, 48, and 72 h) (*n =* 348) or three injections of 4 mg/kg (at 0, 24, and 48 h) either i.m. (*n =* 348) or i.v. (*n* = 351), both of which were the intervention arms. The primary endpoint was the proportion of children with ≥99% reduction in parasitemia at 24 h from admission values, measured by microscopists who were blinded to the group allocations. Primary analysis was performed on the per-protocol population, which was 96% of the intention-to-treat population. Secondary analyses included an analysis of host and parasite genotypes as risks for prolongation of parasite clearance kinetics, measured every 6 h, and a Kaplan–Meier analysis to compare parasite clearance kinetics between treatment groups. A post hoc analysis was performed for delayed anemia, defined as hemoglobin ≤ 7g/dl 7 d or more after admission.

The per-protocol population was 1,002 children (five-dose i.m.: *n =* 331; three-dose i.m.: *n =* 338; three-dose i.v.: *n =* 333); 139 participants were lost to follow-up. In the three-dose i.m. arm, 265/338 (78%) children had a ≥99% reduction in parasitemia at 24 h compared to 263/331 (79%) receiving the five-dose i.m. regimen, showing non-inferiority of the simplified three-dose regimen to the conventional five-dose regimen (95% CI −7, 5; *p =* 0.02). In the three-dose i.v. arm, 246/333 (74%) children had ≥99% reduction in parasitemia at 24 h; hence, non-inferiority of this regimen to the five-dose control regimen was not shown (95% CI −12, 1; *p =* 0.24). Delayed parasite clearance was associated with the ^N86Y^Pfmdr1 genotype. In a post hoc analysis, 192/885 (22%) children developed delayed anemia, an adverse event associated with increased leukocyte counts. There was no observed difference in delayed anemia between treatment arms.

A potential limitation of the study is its open-label design, although the primary outcome measures were assessed in a blinded manner.

**Conclusions:**

A simplified three-dose i.m. regimen for severe malaria in African children is non-inferior to the more complex WHO-recommended regimen. Parenteral ARS is associated with a risk of delayed anemia in African children.

**Trial registration:**

Pan African Clinical Trials Registry PACTR201102000277177

## Abstract 2

In a randomized trial, Sanjeev Krishna and colleagues test for non-inferiority of a simplified artemisinin regimen as treatment for malaria in a low-resource setting.

## Editors' Summary

**Background:**

Globally, about 200 million cases of malaria—a mosquito-borne parasitic disease—occur every year. Malaria infections, which can be caused by several parasites, can be “uncomplicated” or “severe.” Prompt treatment of uncomplicated malaria, which presents with flu-like symptoms, is essential to prevent the development of severe malaria. The World Health Organization (WHO) recommends artemisinin combination therapy (ACT) for the first-line treatment of uncomplicated malaria in countries where the disease is always present. In ACT, artemisinin derivatives (fast-acting antimalarial drugs that are cleared rapidly from the body) are combined with a slower-acting, more slowly eliminated partner drug to prevent the original infection recurring and to reduce the risk of the malaria parasites becoming resistant to either drug. Severe malaria, which is usually caused by *Plasmodium falciparum*, is characterized by anemia and by damage to the brain and other organs. Severe malaria kills more than 400,000 people (mainly young children living in sub-Saharan Africa) every year.

**Why Was This Study Done?:**

WHO recommends that severe malaria be treated with intravenous or intramuscular injections of artesunate, a parenteral (injectable) form of artemisinin; patients with severe malaria cannot take pills reliably or safely. Specifically, WHO recommends that patients be given 2.4 mg of artesunate per kilogram of body weight intravenously or intramuscularly at the time of admission (0 hours) and at 12, 24, 48, and 72 hours (followed by ACT to ensure full parasite clearance). But this five-dose regimen is complex. A simpler regimen would be easier to administer in resource-limited settings, where giving the correct doses on time to small, sick children can be challenging. In this open-label, non-inferiority randomized controlled trial (RCT), the researchers investigate the efficacy of a three-dose artesunate regimen for the treatment of severe malaria in African children. RCTs compare outcomes in people randomly chosen to receive different interventions; in an open-label RCT, both the researchers and the participants know which treatment is being given; a non-inferiority trial investigates whether one treatment is not worse than another treatment.

**What Did the Researchers Do and Find?:**

The researchers randomly allocated 1,047 children aged six months to ten years with severe malaria attending seven clinical centers in five African countries to receive a total dose of 12 mg of artesunate per kilogram of body weight as five intramuscular injections of 2.4 mg/kg given at 0, 12, 24, 48, and 72 hours (the control regimen) or as three intramuscular or intravenous injections of 4 mg/kg given at 0, 12, and 24 hours (three-dose intramuscular and intravenous regimens, respectively). The trial’s primary endpoint was the proportion of children whose parasitemia (parasite count in the blood) at 24 hours was ≤1% of that at admission (in other words, ≥99% parasite clearance). Among the 1,002 children who received the planned drug doses (the per-protocol population), 78% in the three-dose intramuscular group had ≥99% parasite clearance compared to 79% in the five-dose intramuscular group, a result that met a preset criterion for non-inferiority at 24 hours of the three-dose intramuscular regimen to the control regimen. However, because only 74% of the children in the three-dose intravenous group had ≥99% parasite clearance, this regimen was not shown to be non-inferior to the conventional five-dose regimen.

**What Do These Findings Mean?:**

These findings when combined with the findings of several secondary analyses suggest that, in African children, a three-dose intramuscular artesunate regimen is non-inferior to the WHO-recommended regimen for the treatment of severe malaria. The study’s open-label design may limit the accuracy of its findings, as may its use of a primary endpoint midway through drug treatment rather than at the end (the researchers note that 60% of deaths from severe malaria occur during the first 24 hours of illness and that parasitemia is harder to measure later during treatment) and its use of parasite clearance rather than death as the primary endpoint (case fatality rates in severe malaria treatment trials are very low, so a much larger study would be needed if death were used as the primary endpoint). Overall, these findings support the use of the three-dose intramuscular artesunate regimen for the treatment of severe malaria. Importantly, however, 22% of the children in the study developed delayed anemia, irrespective of treatment regimen. Thus, although further studies are needed to clarify whether treatment with artesunate or the malaria infection itself was responsible for the delayed anemia, patients treated with artesunate for severe malaria should be routinely monitored for this complication.

**Additional Information:**

This list of resources contains links that can be accessed when viewing the PDF on a device or via the online version of the article at http://dx.doi.org/10.1371/journal.pmed.1001938.

- Information is available from the World Health Organization on malaria (in several languages); the *World Malaria Report 2014* provides details of the current global malaria situation, including information on malaria in individual African countries; WHO’s *Guidelines for the Treatment of Malaria* and its *Management of Severe Malaria*: *A Practical Handbook* are available
- The US Centers for Disease Control and Prevention provides information on malaria (in English and Spanish), including personal stories about malaria
- The UK National Health Service Choices website also provides information about malaria, including a personal story
- Information is available from the Roll Back Malaria Partnership on the global control of malaria
- The Scientists Against Malaria collaboration applies modern drug design and modeling techniques to develop new treatments against malaria; its website includes information about many aspects of malaria
- Public Health England provides a collection of guidance and research and analysis on malaria
- MedlinePlus provides links to additional information on malaria (in English and Spanish)
- More information about this trial is available

## Introduction

Studies to optimize artesunate (ARS) treatment regimens in malaria have been surprisingly sparse, given that ARS is now established as the treatment of choice for severe malaria in both adults and children [1,2]. WHO recommends ARS (2.4 mg/kg) administered by intravenous (i.v.) or intramuscular (i.m.) routes at 0, 12, 24, 48, and 72 h after admission [2], although simpler regimens would be preferable, assuming that safety and efficacy were not compromised [2]. The advantages of a simpler regimen are obvious to health care workers in under-resourced settings, where finding and maintaining i.v. access in small, sick children to ensure that correct doses are given on time is a challenge [1,3,4].

In an earlier study, a simplified three-dose ARS i.v. regimen was found to be non-inferior in pharmacodynamic efficacy to the conventional WHO regimen [1], and its pharmacokinetics (PK) were defined with a formulation that conformed to standards of good manufacturing practice. Since then, WHO has prequalified another formulation of ARS (Guilin Pharmaceutical, Shanghai, China), making it more widely used. We have also compared the i.v. and i.m. routes of this product for severe malaria in African children and have described the PK of a WHO-recommended dose regimen using ARS [4]. This regimen of one dose of 2.4 mg/kg followed by four doses of 1.2 mg/kg has been superseded by a regimen of five doses of 2.4 mg/kg, and there has since been debate about the simplified (once daily) i.v. regimen for severe malaria [5,6]. The i.m. route has not yet been studied in adequately powered dose optimization trials.

We examined i.m. ARS in severe malaria in seven hospitals of the Severe Malaria in African Children (SMAC) network [7,8]. We assessed whether splitting the total dose of 12 mg/kg into a simplified once daily i.m. or i.v. three-dose regimen (4 mg/kg per dose) is non-inferior to the WHO-recommended five-dose regimen (2.4 mg/kg per dose). We also examined associations of genetic polymorphisms of *pfmdr1* and *kelch-13* with parasite clearance kinetics and, in post hoc analysis, the occurrence of delayed anemia.

Our primary study objective was to assess the non-inferiority of i.v. ARS and i.m. ARS simplified dosing regimens (4 mg/kg ARS at 0, 24, and 48 h; 12 mg/kg total dose) to the standard i.m. treatment dosing regimen (2.4 mg/kg ARS at 0, 12, 24, 48, and 72 h; 12 mg/kg total dose) in clearing parasitemia in African children with severe malaria. Our secondary study objectives were to compare the tolerability and safety of the three ARS dosing regimens, to analyze host and parasite genotypes as risks for prolongation of parasite clearance kinetics, measured every 6 h, and to compare parasite clearance kinetics between treatment groups. An exploratory objective was to analyze genetic polymorphisms in humans and parasites linked to disease and treatment, and a post hoc objective was to assess the occurrence of delayed anemia.

## Methods

The trial was performed according to the principles of the Declaration of Helsinki and Good Clinical Practice. Ethics committees and competent authorities for each study site approved the study. A data monitoring board (DMB) provided oversight for the study with respect to safety and efficacy as well as appropriate implementation of the defined stopping rules.

### Study Design

**Fig 1:**
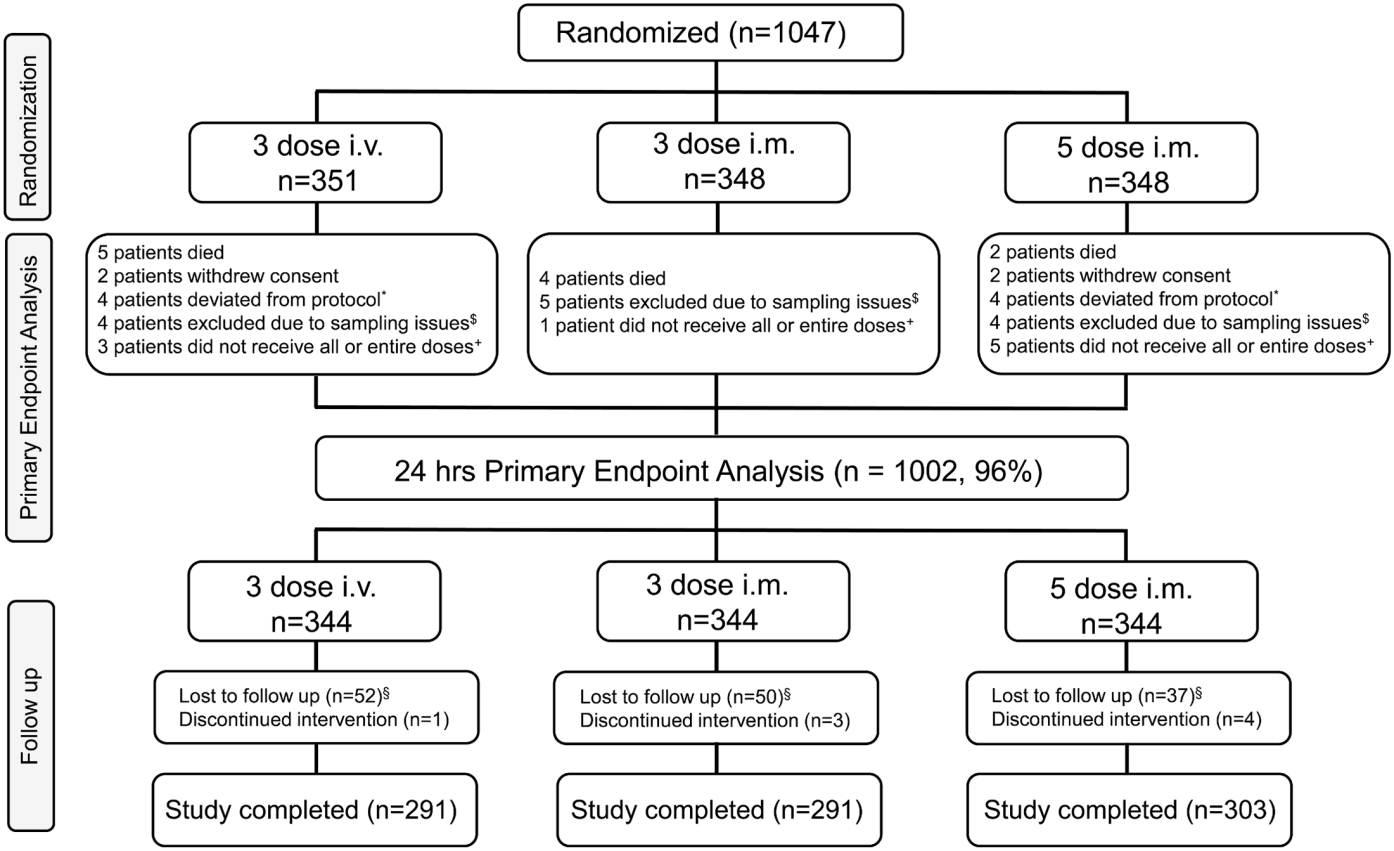
Trial profile. *These patients completed the study but were not included for the primary endpoint analysis because of protocol deviations. ^$^These patients completed the study but were not included for the primary endpoint analysis because of sampling issues. ^+^These patients completed the study but were not included for the primary endpoint analysis because of dosing issues. ^§^Lost to follow-up includes patients who (i) withdrew consent (*n =* 8), (ii) moved from the study area (*n =* 9), and (iii) were discharged from the study due to malaria infection on day 28 (*n =* 1), amongst a variety of other reasons.

This was an open-label, randomized, multicenter, parallel-group, three-arm study to compare the antimalarial activity and safety of three ARS dosing regimens in children with severe *Plasmodium falciparum* malaria. Patients (as shown in Fig 1) were randomly assigned to one of three dosing regimens consisting of a total of 12 mg/kg parenteral ARS: (i) 2.4 mg/kg i.m. on admission and at 12, 24, 48, and 72 h, (ii) 4 mg/kg i.m. on admission and at 24 and 48 h, and (iii) 4 mg/kg i.v. on admission and at 24 and 48 h. Time points of administration of ARS for each group are represented in Table 1.

**Table 1:** Dosing regimen of artesunate.

Group	Route	Dose	0 h	12 h	24 h	48 h	72 h
Five-dose i.m.	i.m.	2.4 mg/kg	X	X	X	X	X
Three-dose i.m.	i.m.	4.0 mg/kg	X		X	X	
Three-dose i.v.	i.v.	4.0 mg/kg	X		X	X	

Parasitemia was measured by thick blood smears at 6-h intervals and prior to the each dose of treatment for at least 48 h following the first dose of study drug, or until three consecutive negative smears were recorded within the last 24-h period. Thick blood films were also examined on days 7, 14, and 28.

The primary efficacy endpoint was the proportion of patients with ≥99% parasite reduction from the baseline asexual parasite count at 24 ± 1 h. Parasitemia was always quantitated before the 24-h dose was administered, i.e., after either one (intervention arms) or two (control arm) doses of ARS had been administered. This endpoint was derived from discussions in the SMAC network and from our own studies [1,4] and is based on the following reasoning. First, WHO guidelines for the treatment of severe malaria [9] are based on studies that have used multiple outcomes. For efficacy, these are death, parasite clearance time, fever clearance time, time to discharge from hospital (days), and coma resolution time. As studies using mortality as an endpoint are impracticable in seasoned centers where overall mortality from severe malaria is <5%—requiring sample sizes that are too large—we have chosen a parasitemia clearance parameter as an endpoint. Parasite clearance kinetics was also used when a quinine loading dose (20 mg/kg salt, if no pretreatment) was being developed and compared with the then standard dose (10 mg/kg salt), when a mortality study comparing the two regimens was ruled out on sample size grounds. Parasite clearance time was a crucial determinant of efficacy for comparing the same drug in two dosing regimens [10]. In severe malaria, clinically advantageous benefits of more rapid clearance of parasites by ARS (even when given by suppository) have been reported in comparison with i.m. artemether [11]. The 24-h time point was chosen as an endpoint because most deaths (>60%) from severe malaria take place within 24 h of admission, and accurate assessment of parasitemia becomes more difficult at later time points.

Evaluation of the whole regimens is included in the secondary outcome measures (and there is no discrepancy between the results for this and the 24-h end point). Further endpoints were time to total clearance of asexual parasites, time to 99% reduction of asexual parasites, time to 90% reduction of asexual parasites, time to 50% reduction of asexual parasites, proportion of patients with genotype-uncorrected adequate clinical and parasitological response on day 28, percent reduction in asexual parasites from baseline at 24 h after initiation of randomized study drug, and percent reduction in asexual parasites from baseline at 48 h after initiation of randomized study drug.

During the conduct of this study, several patients in the case series developed delayed hemolytic anemia following ARS therapy, mostly in the second and third week from the start of therapy [12–15]. Motivated by these events, we amended our trial protocol and undertook two exploratory post hoc analyses of delayed anemia. In the first, all trial participants were screened and treated for delayed anemia, defined as hemoglobin (Hb) ≤ 7 g/dl seven or more days after admission. In the second, a subgroup of 72 patients in Kumasi and Lambaréné who underwent detailed hematological monitoring for 28 d following discharge from the hospital were assessed for hemoglobin and laboratory markers of hemolysis (such as lactate dehydrogenase) during follow-up [16]. For the patients in the subgroup, we were able to assess laboratory markers of hemolysis (such as lactate dehydrogenase) in more intensive follow-up, whereas the last day of scheduled sampling for these particular markers for all other patients in the study was day 7.

### Participants

Children were aged 6 mo to 10 y, with a diagnosis of *P*. *falciparum* infection (parasitemia ≥ 5,000 parasites/μl on initial blood smear) and clinical signs and symptoms severe enough to require hospitalization, according to the SMAC definition of severe malaria that best reflects the policies of African hospitals [7,8]. Most (87%) of these children also fulfilled one or more criteria of the WHO definition of severe malaria [17], which include severe anemia (hematocrit of <15% or Hb < 5 g/dl with a parasite density of >10,000/μl), hyperlactatemia (≥5 mmol/l), hyperparasitemia (>250,000 parasites/μl), hypoglycemia (whole blood or plasma glucose ≤ 2.2 mmol/l), and hemoglobinuria (urine that is dark red or black, with a dipstick that is positive for Hb/myoglobin).

In addition, children were required to be willing and able to comply with the study protocol for the duration of the study, be willing to remain in the hospital for at least 3 d, and have had written informed consent provided by their parents or guardians. Exclusion criteria included known serious adverse reaction or hypersensitivity to artemisinins, any underlying disease that might compromise the diagnosis and evaluation of the response to the study medication, participation in any investigational drug study during the 30 d prior to screening, and adequate (according to WHO and country-specific guidelines) antimalarial treatment within 24 h prior to admission.

Patients were recruited at the Centre de Recherches Médicales de Lambaréné, Lambaréné, Gabon (*n =* 245); Queen Elizabeth Central Hospital, Blantyre, Malawi (*n =* 211); the Université des Sciences de la Santé, Libreville, Gabon (*n =* 150); the School of Medical Sciences at the University of Science and Technology (Komfo Anokye Teaching Hospital), Kumasi, Ghana (*n =* 149), Kenya Medical Research Institute Kondele Children’s Hospital, Kisumu, Kenya (*n =* 129); Edward Francis Small Teaching Hospital (former Royal Victoria Teaching Hospital), Medical Research Council Laboratories, Banjul, The Gambia (*n =* 90); and the Kenya Medical Research Institute Centre for Geographic Medicine–Coast, Kilifi, Kenya (*n =* 73).

A total of 45 participants were lost to follow-up between the time of randomization and the primary endpoint analysis time point at 24 h. Loss to follow-up was due to death, withdrawal of consent, protocol deviations, not receiving all doses, or sampling issues. Sampling issues were defined as physical difficulties in obtaining blood due to the small size of participants and difficulties with venipuncture. A total of 139 participants were lost to follow-up after the 24-h primary endpoint analysis time point. Participants were considered lost to follow-up after the 24-h time point if they were unable to be reached when research staff tried to contact them at least five times at two different times of day via telephone, and with at least two house visits, within a 2-wk period.

Patient participation included hospitalization for at least 3 d and follow-up for at least 28 d following the first dose of study drug. Participants had scheduled follow-up visits in the clinic on days 7, 14, and 28, during which vital sign evaluation, physical examination, adverse event (AE) review, and blood sampling for hematology, biochemistry, parasitological assessments, PK analysis, and exploratory analyses were conducted

### Randomization and Masking

Randomization was balanced at each study site in a 1:1:1 ratio for each regimen. Randomization cards were supplied in numbered, sealed envelopes. The envelope for each participant was opened after inclusion in the trial, directly before treatment initiation. Microscopists were not informed about group allocations.

### Procedures

ARS for injection (Artesun; Guilin Pharmaceutical, Shanghai, China) was supplied as powder and reconstituted before injection. Artemether-lumefantrine was given at discharge in a weight-normalized dosing regimen [18].

Other concomitant therapies were given according to published guidelines [2] and the standard operating procedures of the sites. Malarial infection recurring within 28 d was treated with artemether-lumefantrine.

Malaria smears were done every 6 h until three consecutive negative smears were recorded [19] and were read independently by two microscopists. Vital signs were recorded at least twice daily, and physical examination was done repeatedly, over the period of hospitalization.

Population PK studies were performed on a subset (*n =* 288) of patients for the parent compound ARS, the primary metabolite dihydroartemisinin (DHA) and the secondary metabolite, the primary DHA glucuronide (DHAG), using established population PK techniques that were refined using the results of the first dose optimization study [1]. PK data were available for 39 of the patients in the anemia analysis. Venous samples (400 μl) were collected 30, 60, 120, 240, or 360 min after each of three dosings. Allocation to one of the five predefined sampling time points was random within each treatment arm. Samples were stored at −80°C until use. ARS, DHA, and DHAG concentrations were assayed using liquid chromatography/mass spectrometry [20]. In total, 851 samples were analyzed from three study centers (from 116 patients from Lambaréné, 84 from Kisumu, and 88 from Kumasi). Parasite and host polymorphisms were examined in the following genes using published methods and primers for PCR: *pfmdr1*, *kelch-13*, the sickle cell gene, and the gene for glucose-6-phosphate dehydrogenase (G6PDH) [20,21]. This analysis was carried out on the subset of patients included in the PK analysis.

### Statistical Analysis

Fisher’s exact test for one-sided equivalence [22] was used to assess treatment group differences in parasite clearance for the per-protocol (PP) and intention-to-treat (ITT) populations, and the 95% CI of the difference in proportion is given. Primary analysis was on the PP population, since it is more conservative in non-inferiority models. Testing was done hierarchically, with comparisons of the two experimental arms (three-dose i.m. and three-dose i.v.) against the control (five-dose i.m.), corrected for multiple testing using the Bonferroni method. Only when both tests rejected the null hypothesis was a further test comparing the two experimental arms planned. As secondary endpoints, parasite clearance times were calculated using Kaplan–Meier estimates and a Cox model with treatment arm and study center as covariates, when not otherwise described. Alpha < 0.05 was considered significant.

The needed sample size was calculated based on the results of our prior study [1,6]. We assumed that 82% of patients would have a ≥99% reduction in parasitemia at 24 h (primary endpoint) and set power to 0.8, alpha to 0.05, and delta to 0.1. Using the Farrington and Manning procedure [23] as implemented in the Design package of R v. 2.10.1, the calculated sample size needed was 316 per arm when multiple comparisons between the groups were included. The total estimated sample size needed, with 10% headroom for loss to follow-up, was therefore 1,044 participants.

The non-inferiority margin delta was pre-specified as an absolute difference of 10% for the primary endpoint on the basis of our previous study [1] and published methods for analyzing time-to-event outcomes [24]. For the Cox model, the non-inferiority margin was translated into a hazard ratio (HR) assuming a 82% cure rate (≥99% reduction in parasitemia at 24 h) with the control (five-dose i.m.) regimen and an at least 72% cure rate with the experimental (three-dose i.m. and three-dose i.v.) regimens.

All patients who received at least one dose of the study drug were included in the safety analysis. Delayed anemia was analyzed using a logistic regression model for non-hematological variables, a two-way ANOVA for genotype analysis, and an ANCOVA for hematological variables.

Descriptive statistics for the drug concentration data were calculated for the set of all patients in this study who received the full dose of ARS and who had plasma concentration data available. A population PK model, assuming 100% conversion of ARS to DHA, was developed using the nonlinear mixed-effects modeling software Phoenix NLME 1.2 (Pharsight, St. Louis, Missouri, US). The final population PK models for ARS, DHA, and DHAG were evaluated using visual predictive checks. Plasma concentration over time data were described by a one-compartment PK model with additive residual error and an exponential term for interpatient variability. Initial PK parameter estimates were from our previous SMAC trial [1]. Route of administration, study center, weight, age, height, delayed anemia, parasitemia, and host and parasite genotypes were added as covariates in the model by stepwise forward inclusion. Model improvement by covariates was statistically tested by the decrease in −2 log likelihood. The final population PK model included all covariates associated with a significant increase in log likelihood (5% significance).

### Role of the Funding Source

The clinical sponsor of the trial was Universitätsklinikum Tübingen, and the corresponding author P. G. K. acted as the sponsor’s representative. The corresponding authors had full access to all of the data in the study and had final responsibility for the decision to submit for publication.

## Results

In all, 1,047 patients were randomized and received at least one dose of study drug, as shown in Fig 1. This is the safety population and also defines the ITT population as all patients had *P*. *falciparum* infection. The PP population is defined as all patients from the ITT population who received all doses of randomized study drug and for whom the primary endpoint could be calculated. This is the primary analysis population for efficacy and is 96% of the ITT population (*n* = 1,002).

**Fig 2:**
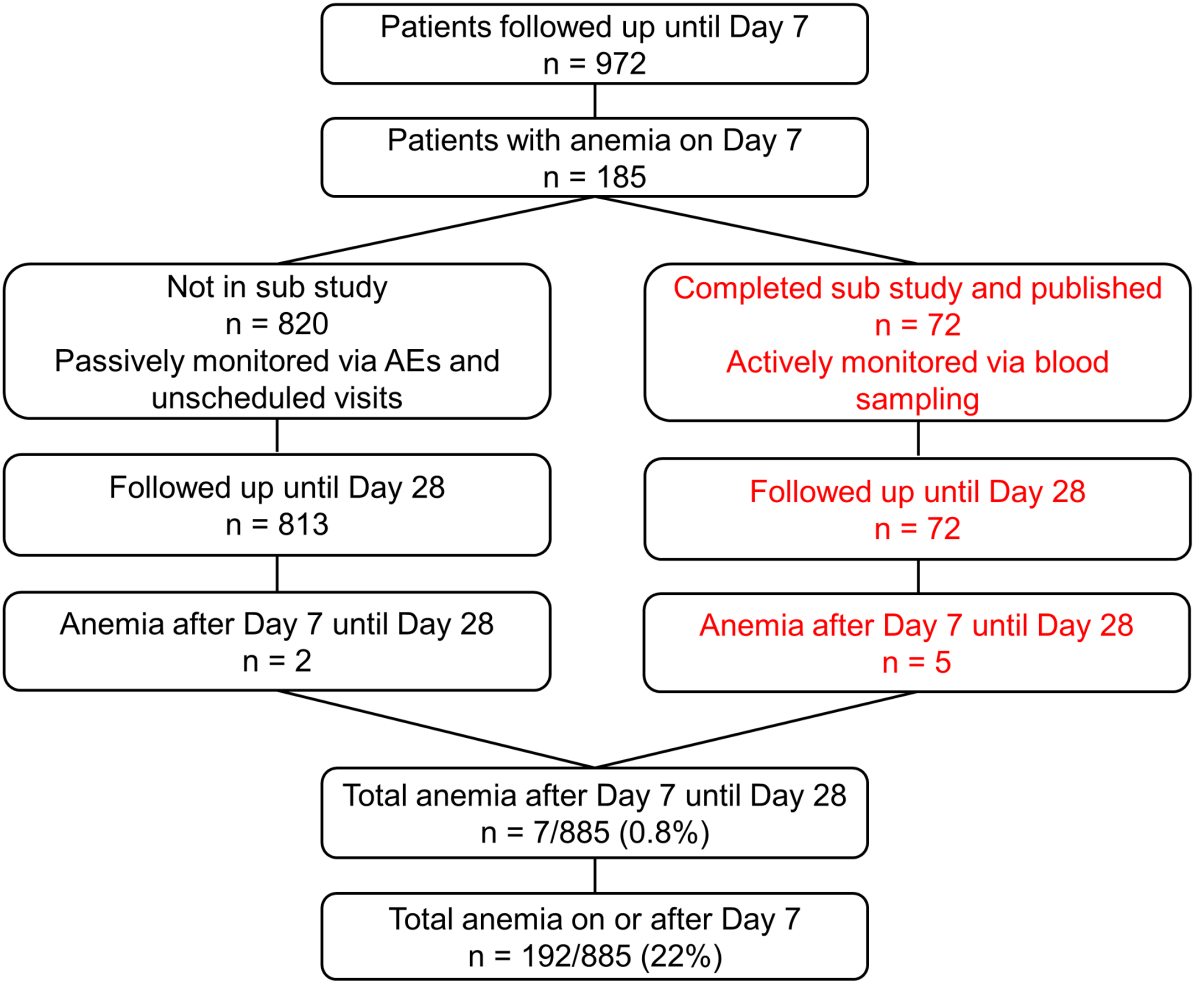
Post hoc analyses of patients with anemia. The right hand side of the diagram (in red) shows patients included in the substudy.

Recruitment was from 4 July 2011 until 25 September 2012. As pre-specified in the protocol, after 50 patients completed the trial procedures up to 72 h, all serious AEs (SAEs) including deaths were reviewed by the DMB, with no findings that required stopping the study. After 100 patients completed the trial up to 24 h in each cohort, parasitemia evaluations were reviewed by the DMB to confirm that in all cohorts, 99% reduction in parasitemia was achieved in at least 60% of patients after 24 ± 1 h (stopping rule). After DMB reviews, there was no finding to stop recruitment. There were to be ad hoc reviews if SAEs and/or deaths in one cohort increased significantly compared with any other. For deaths in any cohort, the threshold to trigger review by the DMB was 4% (stopping rule), but it was not invoked. Anemia was studied in patients as shown in Fig 2. Table 2 summarizes the baseline demographic, clinical, and laboratory variables of patients in the ITT population.

**Table 2:** Patient clinical and laboratory findings on admission in intention-to-treat population.

Variable	Arm			Total (*n =* 1,047)
	Three-Dose i.v. (*n =* 351)	Three-Dose i.m. (*n =* 348)	Five-Dose i.m. (*n =* 348)	
**Clinical findings**				
Female	162 (46%)	166 (48%)	168 (48%)	496 (47%)
Male	189 (54%)	182 (52%)	180 (52%)	551 (53%)
Age, y	4.0 (2.4)	4.1 (2.5)	4.2 (2.5)	4.1 (2.5)
Aged 0–3 y	201 (57%)	185 (53%)	185 (53%)	571 (54%)
Aged 4–7 y	122 (35%)	132 (38%)	130 (37%)	384 (37%)
Aged >8 y	28 (8%)	31 (9%)	33 (10%)	92 (9%)
Pulse, beats/minute	134 (26)	133 (25)	133 (25)	133 (25)
Respirations/minute	40 (13)	39 (11)	40 (13)	40 (12)
Temperature, °C	38.2 (1.2)	38.1 (1.2)	38.1 (1.2)	38.1 (1.2)
Weight, kg	14.3 (5.3)	14.3 (5.0)	14.8 (5.1)	14.4 (5.5)
**Laboratory findings**				
Parasitemia per microliter × 10^−3^ *	129.0 (4.6–2,965.0)	114.0 (5.0–1,439.0)	119.0 (4.1–2,675.0)	121.0 (4.1–2,965.0)
Hb, g/dl	8.5 (2.4)	8.7 (2.3)	8.7 (2.4)	8.6 (2.4)
White blood cell count, 10^3^/μl	10.3 (5.5)	10.1 (5.2)	9.7 (4.6)	10.0 (5.1)
Neutrophils, 10^3^/μl	5.5 (3.2)	5.3 (3.0)	5.4 (3.3)	5.4 (3.2)
Platelet count, 10^3^/mm^3^	115 (117)	107 (108)	123 (130)	115 (119)
**Clinical signs of severe malaria**				
Severe anemia	34 (10%)	42 (12%)	40 (11%)	116 (11%)
Hyperlactatemia	21 (6%)	18 (5%)	23 (7%)	62 (6%)
Hyperparasitemia	103 (29%)	91 (26%)	103 (30%)	297 (28%)
Hypoglycemia	13 (4%)	7 (2%)	18 (5%)	38 (4%)
Jaundice	28 (8%)	29 (8%)	25 (7%)	82 (8%)
Hemoglobinuria	6 (2%)	6 (2%)	7 (2%)	19 (2%)
Respiratory distress	30 (9%)	35 (10%)	28 (8%)	93 (9%)
Severe vomiting	25 (7%)	36 (10%)	26 (7%)	87 (8%)
Prostration	169 (48%)	160 (46%)	140 (40%)	469 (45%)
Cerebral malaria	32 (9%)	28 (8%)	23 (7%)	83 (8%)
Generalized seizures	39 (11%)	44 (13%)	28 (8%)	111 (11%)

Data are given as mean (standard deviation) or *n* (percent), except for parasitemia per milliliter, which is given as geometric mean (range). Clinical classification was according to the following definitions: severe anemia (hematocrit of <15% or Hb < 5 g/dl with a parasite density of >10,000/μl), hyperlactatemia (≥5 mmol/l), hyperparasitemia (>250,000 parasites/μl), hypoglycemia (whole blood or plasma glucose ≤ 2.2 mmol/l), and hemoglobinuria (urine that is dark red or black, with a dipstick that is positive for Hb/myoglobin).

*PP population.

**Fig 3:**
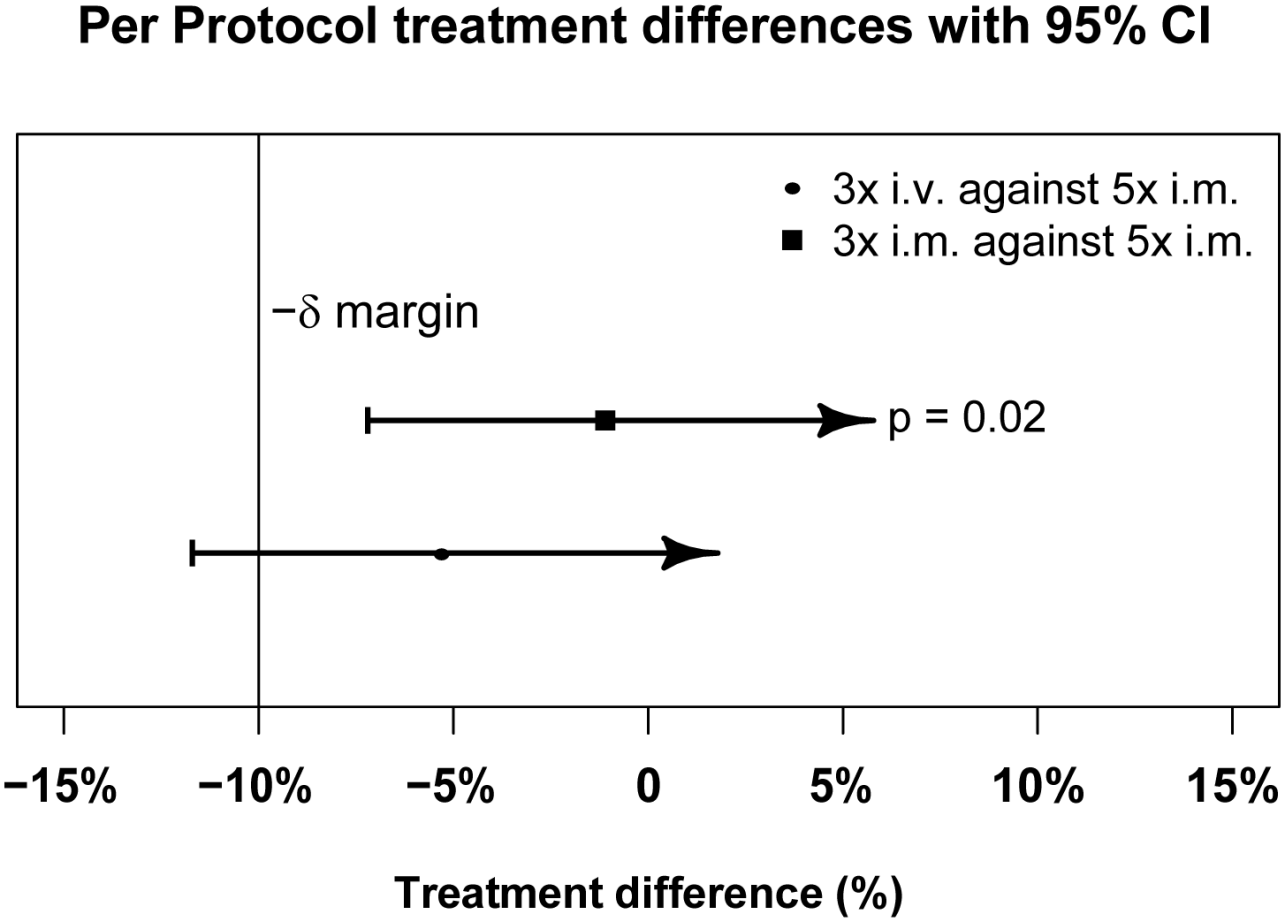
Per-protocol population primary endpoint analysis. PP treatment difference in proportions of patients with ≥99% parasite reduction, with corresponding 95% confidence intervals. The vertical line indicates the non-inferiority margin (δ). The three-dose i.m. treatment group is non-inferior to the five-dose i.m. treatment group (*p =* 0.02), whereas the three-dose i.v. group is not non-inferior (*p =* 0.24). Note that the *p*-value is calculated using Fisher’s exact test for one-sided equivalence under the assumption that both regimens are equally efficacious.

**Fig 4:**
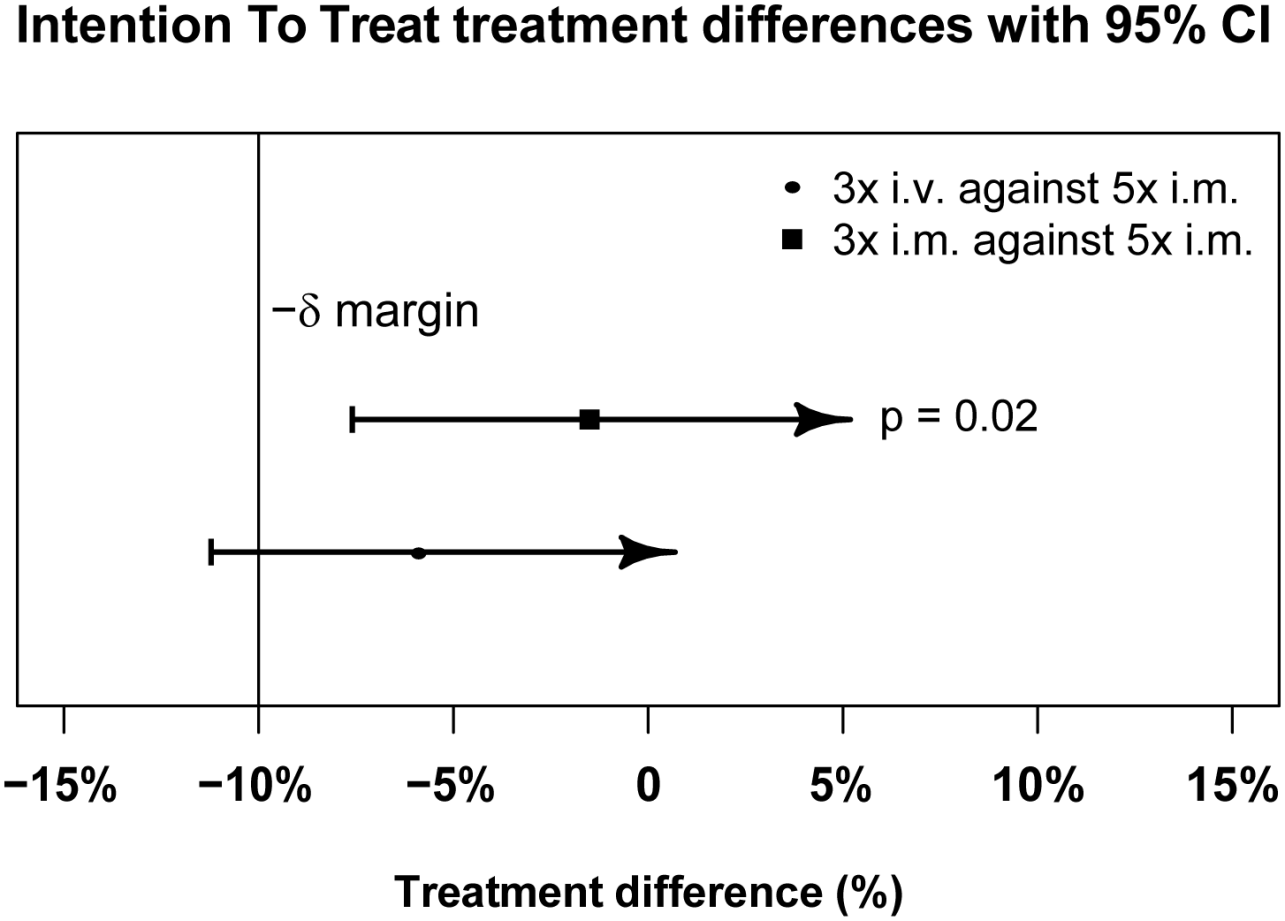
Intention-to-treat population primary endpoint analysis. ITT treatment difference in proportions of patients with ≥99% parasite reduction, with corresponding 95% confidence intervals. The vertical line indicates the non-inferiority margin (δ). The three-dose i.m. treatment group is non-inferior to the five-dose i.m. treatment group (*p =* 0.02), whereas the three-dose i.v. group is not non-inferior (*p =* 0.24). Note that the *p*-value is calculated using Fisher’s exact test for one-sided equivalence under the assumption that both regimens are equally efficacious.

**Fig 5:**
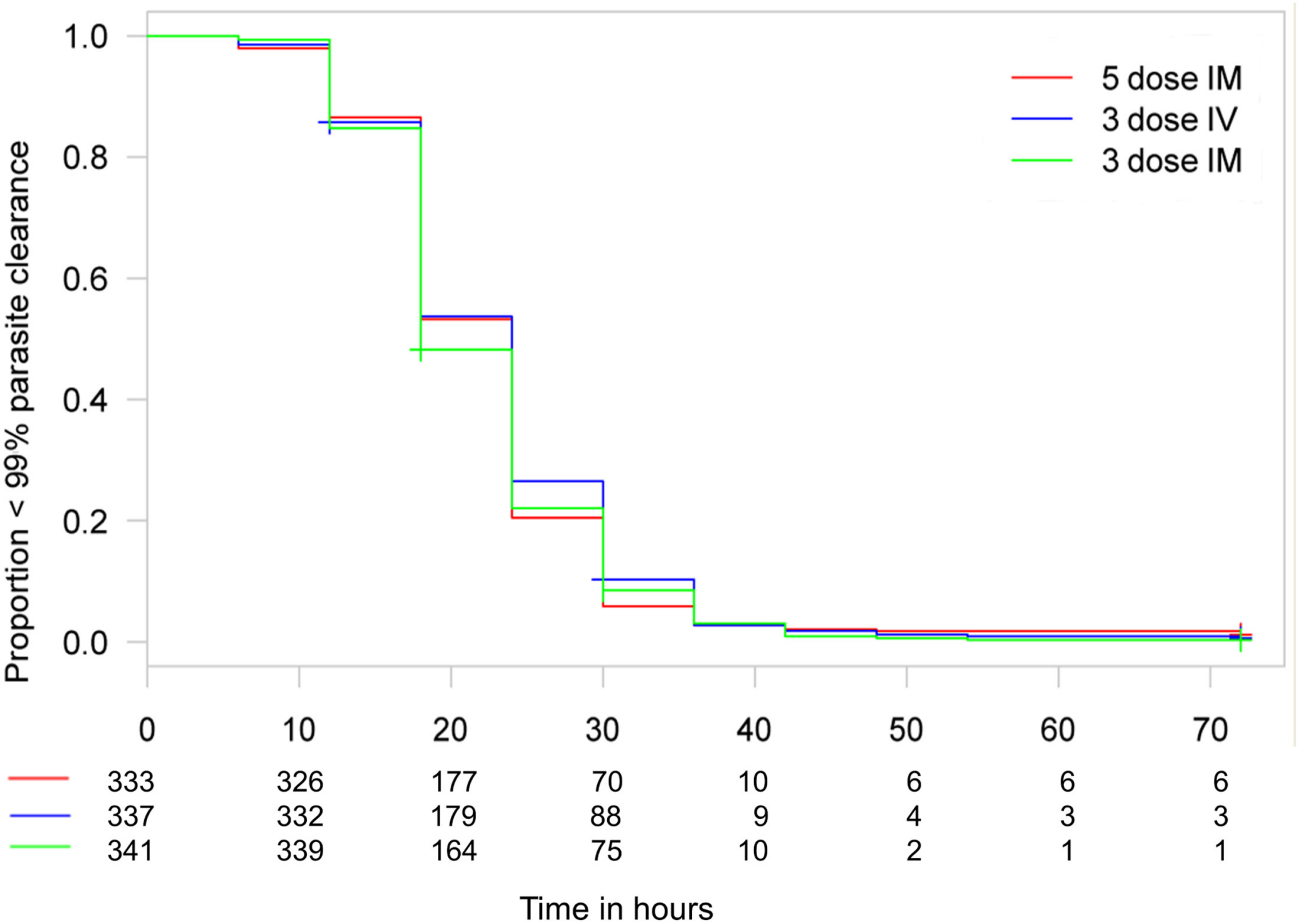
Kaplan–Meier plot for time to 99% parasite clearance in the per-protocol population. Time to 99% parasite clearance under parenteral ARS treatment is shown. Using the 10% delta at 24 h, both three-dose regimens are non-inferior to the five-dose regimen in a Cox proportional hazards model. The PP population has been used for the secondary endpoints.

**Fig 6:**
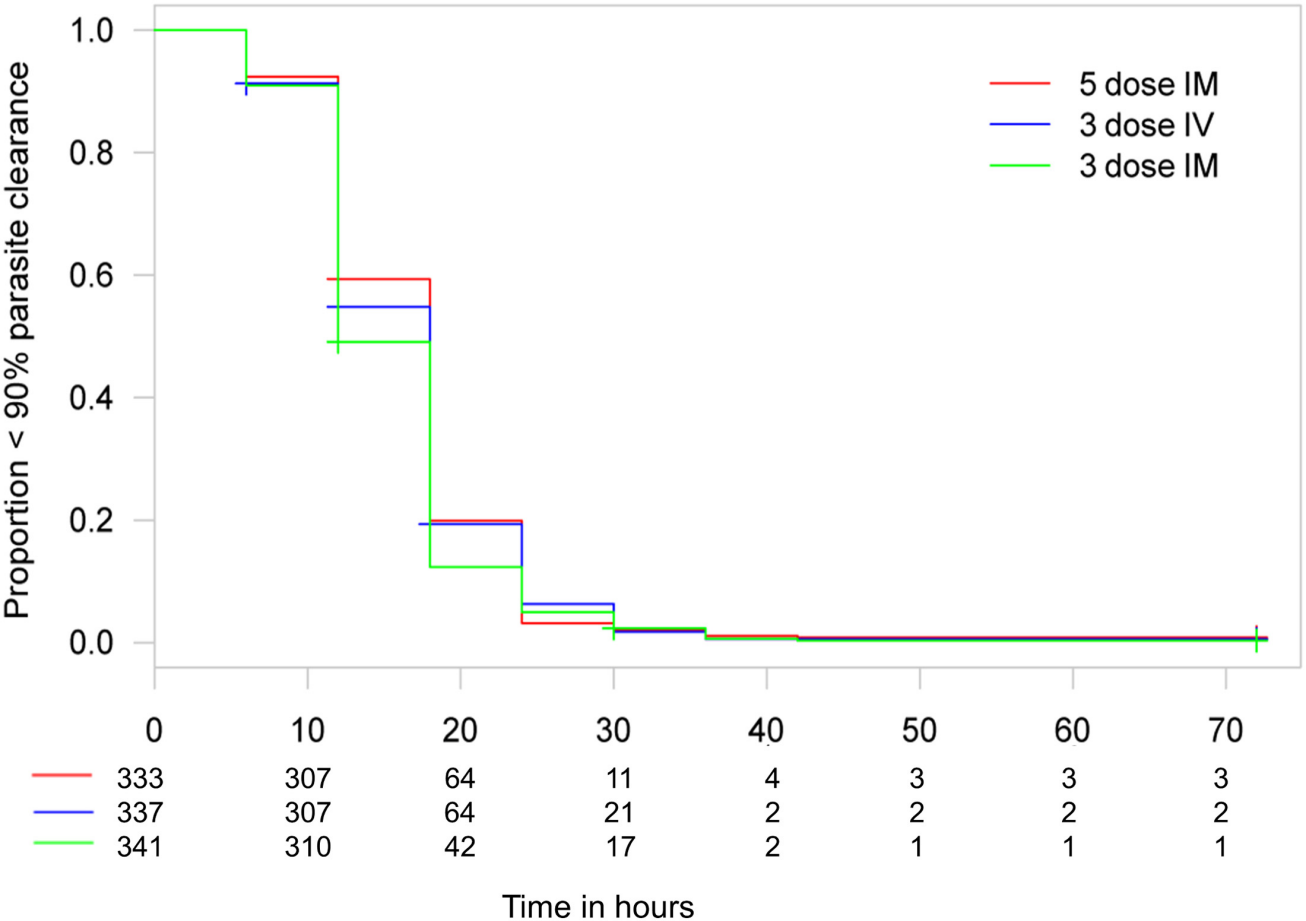
Kaplan–Meier plot for time to 90% parasite clearance in the per-protocol population. Time to 90% parasite clearance under parenteral ARS treatment is shown. Using the 10% delta at 24 h, both three-dose regimens are non-inferior to the five-dose regimen in a Cox proportional hazards model. The PP population has been used for the secondary endpoints.

^N86Y^Pfmdr1 was found in 107/287 (37%) parasites and was associated with delayed time to 99% and 100% parasite clearance estimates of 2.8 h (95% CI 0.9–4.8 h; *p =* 0.005) and 4.8 h (95% CI 1.9–7.6 h; *p <* 0.001), respectively. No other ^N86Y^Pfmdr1 polymorphisms (Y184F, S1034C, N1042D, and D1246Y), including increased gene copy number of *pfmdr1* (found in 13 samples, 5%), were associated with time to parasite clearance. There were no previously reported polymorphisms in *kelch-13* sequences (M476I, Y493H, R539T, I543T, and C580Y). Tests of associations between parasite genotypes and clearance time estimates were corrected for center and treatment group.

Figs 3 and 4 present evidence that the three-dose i.m. route for ARS is non-inferior to the WHO-recommended five-dose i.m. regimen. Both i.m. routes had a higher proportion of patients with ≥99% reduction in parasitemia at 24 h (78%, or 265/338, for the three-dose i.m. group and 79%, or 263/331, for the five-dose i.m. group) (by about 5%) than the three-dose i.v. route (74%, or 246/333). This three-dose i.v. regimen had previously been found to be comparable in efficacy to the WHO-recommended five-dose i.v. regimen [1]. Time to 99% parasite clearance, specified as a secondary analysis, was comparable between treatment groups (Fig 5; S1 Table). Since the Fisher’s exact test for one-sided equivalence does not account for stratifying covariates, which may be anti-conservative, the robustness of the results was tested with Cox proportional hazards models adjusted for study center, which showed non-inferiority (lower confidence interval limit of the HR > 0.74) [24,25] for all comparisons (three-dose i.m. versus five-dose i.m., HR 1.04 [97.5% CI 0.88–1.24]; three-dose i.v. versus five-dose i.m., HR 0.89 [97.5% CI 0.75–1.06]; three-dose i.m. versus three-dose i.v., HR 1.18 [95% CI 1.00–1.37]). For the three treatment groups, the estimates of time to 90% parasite clearance, adjusted for center and initial parasitemia, were significantly different (Fig 6). This difference can be attributed to a more rapid clearance in the three-dose i.m. group compared with the five-dose i.m. group (HR 1.21 [95% CI 1.04–1.41]). In addition, no difference was seen in fever clearance time between groups (S3–S5 Tables). No case required rescue treatment before discharge from hospital, and 16 patients died, with no group differences. There were 41/885 (5%) patients who were parasitemic at 28 d: 13 in the three-dose i.v. group, 11 in the three-dose i.m. group, and 17 in the five-dose i.m. group.

**Fig 7:**
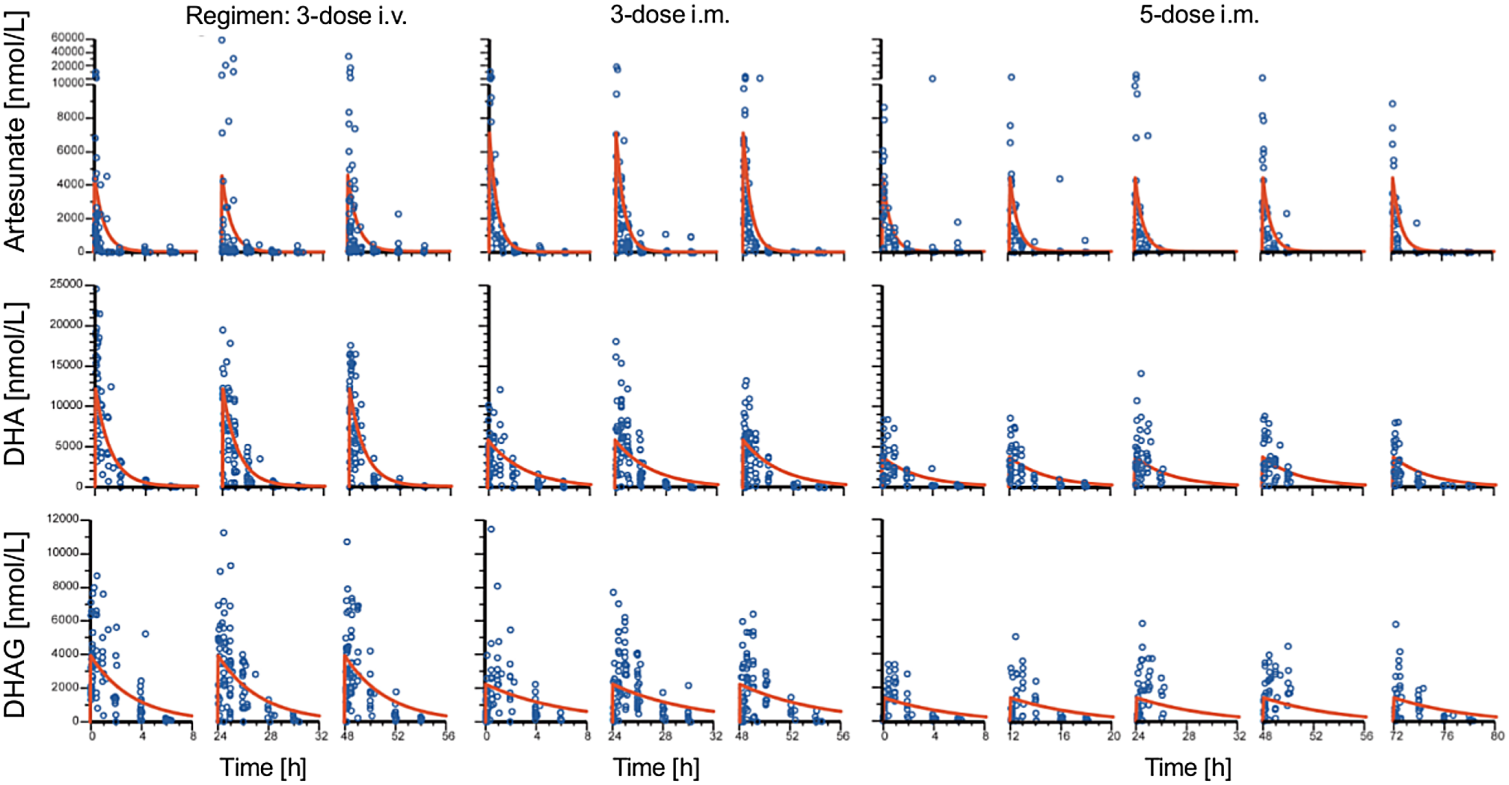
Population pharmacokinetic profiles of artesunate, dihydroartemisinin, and dihydroartemisinin glucuronide. Plots of observed concentration–time profiles for ARS and its major metabolites, DHA and DHAG, are presented according to treatment regimen. Estimated population mean PK profiles are shown by red lines. The three columns of results for each regimen represent the findings after each dose.

Drug detection was linear, with ranges of 1–2,500 nM, 165–16,500 nM, and 4–10,000 nM for ARS, DHA, and DHAG, respectively. In total, 851 samples from 288 patients (153 male and 135 female, aged 0.5 to 10 y, mean 3.8 y) were analyzed: 92, 99, and 97 patients received the five-dose i.m., three-dose i.m., and three-dose i.v. regimens, respectively. The population estimates of PK parameters of the base models are presented in Table 3. These data confirm that the three regimens studied are comparable in their PK parameters, with the exception of a larger volume of distribution of DHA following i.m. injection. In particular, estimates of time to clearance were comparable between groups. Fig 7 presents plots of observed concentration–time profiles for ARS, DHA, and DHAG according to treatment regimen. Estimated population mean PK profiles are shown by the red lines. Interestingly, ARS plasma concentrations varied more after i.v. than after i.m. administration. Study center, age, sex, weight, parasitemia, and delayed anemia were considered as covariates. Of these covariates, parasitemia influenced volume of distribution after i.v. ARS only, while the strongest influence was seen for study center on volume of distribution/bioavailability and clearance of ARS (i.m.), DHA, and DHAG.

**Table 3:** Population pharmacokinetic analysis of parenteral artesunate in severe malaria.

Arm	Drug Metabolite	Volume of Distribution (Liters) (95% CI)	Clearance (Liters/Hour) (95% CI)
**Three-dose i.v.**	ARS	32.0 (22.3–41.6)	42.0 (28.9–55.1)
	DHA	12.0 (10.2–13.8)	9.9 (8.4–11.3)
	DHAG	36.6 (32.0–41.3)	10.8 (9.0–12.7)
**Three-dose or five-dose i.m**	ARS	21.1 (18.2–24.0)	33.3 (29.5–37.1)
	DHA	25.3 (22.4–28.2)	8.5 (7.4–9.5)
	DHAG	66.5 (58.2–74.8)	10.1 (8.3–11.9)

The occurrence of laboratory and clinical AEs and SAEs (Table 4) was similar in the three groups. Out of 75 SAEs, 14 (five severe anemia, six persistent fever, two vomiting, and one cough) were judged as possibly related to the study drug.

**Table 4:** Serious adverse events in the intention-to-treat population.

SAE Outcome	Arm			Total
	Three-Dose i.v.	Three-Dose i.m.	Five-Dose i.m.	
SAEs	26	28	21	75
SAEs with a possible relationship to study drug	5 (19%)	3 (11%)	6 (29%)	14
SAEs with no relationship to study drug	21 (81%)	25 (89%)	15 (71%)	51
Deaths	6	6	4	16
Neurological sequelae after study completion (day 28)	3	1		4

Data are given as *n* or *n* (percent).

During the conduct of this trial, a series of case reports described delayed hemolytic anemia in travelers after receiving artemisinins [26] and prompted an urgent evaluation of the risk of delayed hemolysis in a subset of our patients in Kumasi and Lambaréné, in whom detailed hematological monitoring after discharge was possible, as an amendment to the protocol [16]. For these patients, we defined delayed hemolysis as the coexistence of (i) low haptoglobin (<0.30 mg/dl) on day 14, (ii) any decrease in hemoglobin between days 7 and 14, and (iii) any increase in lactate dehydrogenase between days 7 and 14 leading to a lactate dehydrogenase level of over 350 U/l on day 14.

This substudy identified five out of 72 evaluable patients with anemia between days 7 and 28 [16]. In a post hoc analysis, we also included anemia detected by hemoglobin measurement on day 7 or later. This increased the total number of patients with anemia (Hb ≤ 7 g/dl) a week or more after the start of therapy to 192, although most were detected on day 7 due to sampling bias, as the last scheduled blood sampling per protocol in our study was day 7. We also investigated the AEs in all our patients (including those reported earlier [16]) by assessing anemia at passive follow-up after day 7, and detailed information is given in Table 5. This analysis includes the 72 patients from our earlier reported substudy [16].

**Table 5:** Anemia occurrences from day 7 until day 28.

Anemia Outcome	Number of Patients
Total patients with at least one late anemia episode	192 out of 885 followed up until day 28
Hb ≤ 7 g/dl on day 7	185 out of 972 followed up until day 7
Hb ≤ 7 g/dl on day 28	7
Late transfusion (> day 7)	4
Anemia on active follow-up (substudy)	5

**Fig 8:**
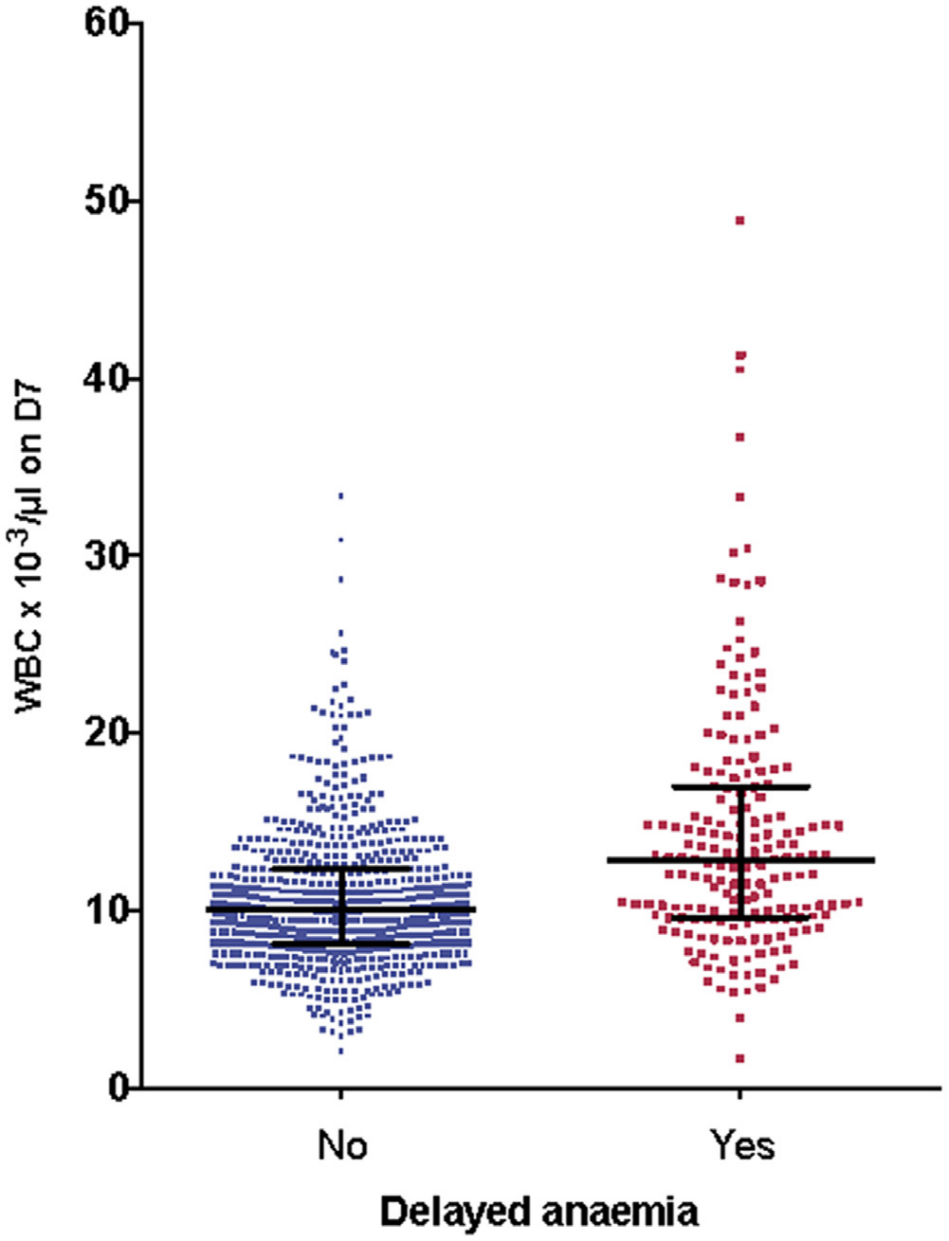
Association between delayed anemia and increased white blood cell count at day 7. Individual white cell counts (WBC) on day 7 (D7) are presented as medians and interquartile ranges, divided into those who developed delayed anemia (12.8 × 10^3^/μl, interquartile range 9.6–17, *n* = 186) and those who did not (10.1 × 10^3^/μl, interquartile range 8.1–12.4, *n* = 689). Patients with delayed anemia had a significantly higher white blood cell count at day 7 (*p <* 0.001).

We examined the relationships between other hematological variables, parasitemia, and delayed anemia, and allowed for admission values by including these in covariance analysis of the full dataset. There was no significant association between delayed anemia and admission parasitemia (*p =* 0.30) or platelet counts on day 7 (*p =* 0.11). A significant association emerged with leukocyte count (*p <* 0.001) and neutrophil count (*p <* 0.001) on day 7. Those with delayed anemia had higher leukocyte (Fig 8) and neutrophil counts than those without delayed anemia, regardless of the definition applied for delayed anemia.

G6PDH-deficient participants (A−) had more anemia on admission (odds ratio 4.3 [95% CI 2.1–9.0], *p <* 0.001) than those with G6PDH non-deficient genotypes, but this relationship did not hold for delayed anemia (odds ratio 1.4 [95% CI 0.6–3.2]). HbAC or HbAS genotype was not associated with delayed anemia, and there was no relationship between delayed anemia and PK parameters.

## Discussion

This study consolidates previous work aiming to optimize dosage regimens using parenteral ARS for severe malaria [1]. Here, simpler three-dose i.m. and i.v. regimens have been compared to the WHO-recommended five-dose i.m. regimen. This study provides new insights into ARS PK, delayed anemia, and genetic markers of delayed parasite clearance. Our studies used the pharmacodynamic endpoint of parasite clearance rather than other clinical outcome measures such as mortality because the latter requires impracticably large studies (as debated [5,6]). In comparing the same drug given in different doses and by different routes, parasite clearance kinetics should accurately reflect differences in in vivo antiparasitic activity because the mechanism of action is the same. It follows that if there are no differences in parasite clearance kinetics between treatments, then they can be considered to be of comparable efficacy. For uncomplicated malaria, for example, inadequate oral dosing with ARS is associated with prolonged parasite clearance kinetics, and in severe malaria a loading dose of quinine significantly shortened parasite clearance times compared with a non-loading-dose regimen [10,26].

From our previous study, we concluded that a simplified once daily regimen was non-inferior in efficacy to the conventional i.v. ARS dosing regimen [1]. This study establishes that the once daily i.v. is not consistently non-inferior to the three-dose and five-dose i.m. regimens. There are several corollaries to this observation. First, clearance kinetics with i.v. once daily ARS in this study (74% achieved ≥99% clearance from baseline at 24 h) is very similar to the previous result of 76% with i.v. once daily ARS. This latter result was also comparable to the five-dose WHO-recommended i.v. regimen in that smaller study and points to a preference for the i.m. route because it is associated with faster clearance kinetics (Figs 5–7). Second, these results confirm that the endpoint chosen is both robust and pharmacodynamically sensitive as a measure of ARS antimalarial efficacy. Also, a once daily simplified i.m. regimen is of comparable efficacy to the five-dose WHO-recommended i.m. regimen, a finding that was robust when analyzed using different statistical methodologies. This finding has important implications for practice. The i.m. route for administration of antimalarials is preferable to the i.v. route in small children [27]. Analysis of secondary endpoints of parasite clearance supports the results of the primary endpoint analysis, and suggests that the once daily i.m. regimen has an even faster time to 90% parasite clearance than the conventional i.m. regimen (median 12 versus 18 h; Figs 5 and 6; S1 Table).

The frequently observed ^N86Y^Pfmdr1 polymorphism has previously been associated with increased sensitivity to artemisinin in in vitro assays in Senegal [28] and decreased sensitivity to artemether in Nigeria [29], with no effect in Thai isolates [30]. Our results provide in vivo evidence for decreased sensitivity to ARS (DHA) of parasites with ^N86Y^Pfmdr1. Changes in the frequencies of polymorphisms in *pfmdr1* are clearly worth monitoring in future epidemiological studies. High unadjusted cure rates (95%) in our patients may reflect the large artemisinin (24 mg/kg total dose of ARS and artemether) component of the treatment course and efficacy of the combination partner (lumefantrine).

The results of our population PK analysis are consistent with classical and previous population PK studies on parenteral ARS [4,6], including in Tanzania [31]. A suggestion, based on modeling, that doses higher (~3 mg/kg per dose for children <10 kg) [31] than currently recommended by WHO may be needed for smaller children is obviated if our higher dose (4 mg/kg) and simpler regimen is implemented. As noted previously, there was no relationship between PK parameters and efficacy. We also present, to our knowledge for the first time, an analysis of the glucuronide derivative of DHA (Fig 7; Table 3). This is quantitatively the most important primary metabolite of DHA. Although it has poor antimalarial activity (IC_50_ of DHAG = 5.7 μM, mean of two experiments), DHAG plasma concentrations commonly peaked above 5 μM (Fig 7), rendering a contribution to parasite clearance possible. Repeated dosing with ARS, in any regimen, does not show evidence of accumulation (Fig 7). This analysis also allows us to examine other potential mechanisms for toxicity, which hitherto has not been possible.

During the conduct of our trial a series of case reports appeared about delayed anemia in travelers who had received parenteral ARS. Therefore, we amended the study protocol to address the occurrence of delayed hemolytic anemia in our ongoing study. We identified delayed anemia after ARS treatment using a small subgroup of patients from the current study [16] in whom it was possible to study anemia defined by several criteria (low haptoglobin, elevated lactate dehydrogenase level, decrease inHb, exclusion of sickle cell disease and G6PDH deficiency). Secondly, in a post hoc analysis that included the whole study population, we defined delayed anemia as Hb ≤ 7 g/dl 7 d or more after admission. We confirmed the occurrence of delayed anemia in a significant proportion (22%) of African children by using this simplified definition that may therefore have greater practical utility. This definition of anemia does not reveal any relationships with PK parameters for ARS, DHA, or indeed DHAG. Instead, a higher leucocyte (Fig 8) and neutrophil count at day 7 is associated with delayed anemia, suggesting that if ARS is the cause of anemia, the mechanism does not involve bone marrow toxicity, because ARS can cause dose-dependent neutropenia [25]. In a recent study of 60 travelers treated with ARS for severe malaria, 13/66 (22%) had delayed anemia, which compares well with our findings. In this study, pitting was significantly associated with delayed anemia [32]. Pitting is a process whereby dead early-stage parasites are removed from erythrocytes by the spleen. Pitting may contribute to the pathophysiology of delayed anemia, which is associated with markers of delayed hemolysis, although available findings from AQUAMAT do not support this [33]. In the AQUAMAT study, the incidence of post-admission severe anemia (Hb < 50 g/l) was comparable in the quinine (5.7%) and ARS (4.6%) groups [34], and both groups had identical proportions of patients (55%) receiving blood transfusions. It is unfortunate that only neurological sequelae were monitored after discharge in the AQUAMAT study, as the risks of delayed anemia with ARS compared to quinine could have been quantified in this cohort.

Weaknesses of this study are its open-label design, which may introduce biases in outcome variables, although allocation bias was minimized and the primary outcome measures of parasite clearance were assessed in a blinded way. The primary analysis at 24 h was before the full treatment regimens had been completed, which may appear as a study limitation unless all parasite clearance estimates are also considered. Additionally, delayed anemia was first described in travelers—and could therefore be addressed—only after the study was mostly complete. It was studied in detail in 72 patients [16], with the remaining analysis being performed post hoc. As there was no comparator arm using a drug other than ARS, further studies will be needed to clarify the impact of artemisinins on delayed anemia. Some colleagues outside the SMAC network may argue that mortality needs to be an endpoint in a study with severe malaria. However, our studies show that case fatality rates in severe malaria trials performed following principles of good clinical practice should not exceed 5%, but rather be between 1% and 2%, regardless of whether the WHO definition or our SMAC definition of cases is used [35]. Thus, death as an endpoint is precluded by sample size requirements.

Simplifying ARS usage with a once daily i.m. regimen in severe malaria is supported by our results, but because delayed anemia is common, patients should be monitored for this complication.

## Supporting Information

S1 Data Dataset used for the analysis. (XLSX)

S1 Table ≥99% parasite clearance 24 h after treatment initiation for the per-protocol population. (DOCX)

S2 Table Time to parasite clearance (hours) for the intention-to-treat population. (DOCX)

S3 Table Fever clearance times, 37.5°C threshold, for the per-protocol population. (DOCX)

S4 Table Fever clearance times, 37.7°C threshold, for the per-protocol population. (DOCX)

S5 Table Fever clearance times, 38.0°C threshold, for the per-protocol population. (DOCX)

S6 Table Biochemical measurements for the intention-to-treat population. (DOCX)

S7 Table Study design. (DOCX)

S1 Text Protocol. (PDF)

S2 Text CONSORT statement. (DOC)

## Abbreviations

AE: adverse event
ARS: artesunate
DHA: dihydroartemisinin
DHAG: dihydroartemisinin glucuronide
DMB: data monitoring board
G6PDH: glucose-6-phosphate dehydrogenase
Hb: hemoglobin
HR: hazard ratio
ITT: intention-to-treat
i.m.: intramuscular
i.v.: intravenous
PK: pharmacokinetics
PP: per-protocol
SAE: serious adverse event
SMAC: Severe Malaria in African Children
