# Supplementary material for: Intramuscular Artesunate for Severe Malaria in African Children: A Multicenter Randomized Controlled Trial
Source: PLoS Med. 2016 Jan 12;13(1):e1001938. doi: 10.1371/journal.pmed.1001938 (PMC4710539; doi:10.1371/journal.pmed.1001938)
Supplement: S1 Table — (DOCX) [file pmed.1001938.s002.docx]

**S1 Table 99% parasite clearance 24 hours after treatment initiation for PP population**

|  | **3 dose iv** | **3 dose im** | **5 dose im** |
| --- | --- | --- | --- |
| **PC99 ≤ 24 hs (n)** | **248** | **266** | **264** |
| **PC99 > 24 hs (n)** | **85** | **72** | **67** |
| **Fraction PC99 ≤ 24 hs** | **74%** | **79%** | **80%** |
