# Supplementary material for: Intramuscular Artesunate for Severe Malaria in African Children: A Multicenter Randomized Controlled Trial
Source: PLoS Med. 2016 Jan 12;13(1):e1001938. doi: 10.1371/journal.pmed.1001938 (PMC4710539; doi:10.1371/journal.pmed.1001938)
Supplement: S2 Table — (DOCX) [file pmed.1001938.s003.docx]

**S2 Table Time to parasite clearance (hours) for ITT population**

PC = parasite clearance
