# Supplementary material for: Intramuscular Artesunate for Severe Malaria in African Children: A Multicenter Randomized Controlled Trial
Source: PLoS Med. 2016 Jan 12;13(1):e1001938. doi: 10.1371/journal.pmed.1001938 (PMC4710539; doi:10.1371/journal.pmed.1001938)
Supplement: S3 Table — (DOCX) [file pmed.1001938.s004.docx]

**S3 Table** Fever Clearance Times, 37.5°C threshold for PP population

Shown are Fever Clearance Times (FCT) for each cohort. FCT is defined as the time from baseline until the start of the period, in which the body temperature remained below the respective threshold for at least 24 hours. Estimates are comparable across cohorts.
