# Supplementary material for: Intramuscular Artesunate for Severe Malaria in African Children: A Multicenter Randomized Controlled Trial
Source: PLoS Med. 2016 Jan 12;13(1):e1001938. doi: 10.1371/journal.pmed.1001938 (PMC4710539; doi:10.1371/journal.pmed.1001938)
Supplement: S6 Table — (DOCX) [file pmed.1001938.s007.docx]

***S6 Table:* Biochemical measurements for ITT population**

All available biochemical measurements are shown as median with corresponding interquartile range.
