# Supplementary material for: Intramuscular Artesunate for Severe Malaria in African Children: A Multicenter Randomized Controlled Trial
Source: PLoS Med. 2016 Jan 12;13(1):e1001938. doi: 10.1371/journal.pmed.1001938 (PMC4710539; doi:10.1371/journal.pmed.1001938)
Supplement: S7 Table — (DOCX) [file pmed.1001938.s008.docx]

***S7 Table:* Study design**

| **Cohorts** | Patients randomized to:  1. iv artesunate 4 mg/kg initially, and at 24 and 48 hours (12 mg/kg total)  2. im artesunate 4 mg/kg initially, and at 24 and 48 hours (12 mg/kg total)  3. im artesunate 2.4 mg/kg initially, and at 12, 24, 28 and 72 hours (12 mg/kg total) |
| --- | --- |
| **Minimum hospitalisation period post artesunate treatment** | 3 days |
| **Follow up visits at site** | Days 7, 14 and 28 |
| **Total length of minimum follow up** | 28 days |
| **Screening assessments** | Demographic information, medical history, physical examination, clinical examination, vital signs, laboratory evaluations, parasitological examination, non-invasive oto-acoustic test, LODS, genetic polymorphisms in humans and parasites linked to disease |
| **Interim assessments** | Physical examination, clinical examination, adverse events and concomitant medications, safety laboratory evaluations, hematology and biochemistry, parasitological examination (thick blood smears), blood samples for pharmacokinetic analysis.  Oto-acoustic tests, LODS, genetic polymorphisms as indicated by reappearance of parasites, in vitro drug sensitivity of re-appearing parasites. |
| **Concomitant treatment** | Other antimalarial (e.g. sulfadoxine-pyrimethamine) at discharge.  Adjunctive therapy according to SMAC standards. |
| **In case of severe reaction to artesunate or treatment failure** | Parenteral quinine. Recurrent malarial infection within 28 days treated with artemether/lumefantrine. |
| **Stopping rules** | 1. Failure to achieve at least 60% of patients for 99% parasite reduction at 24 hours (+/- 1 hour) in one arm after 100 enrolled children of this arm  2. More than 5 out of 20 enrolled children die in hospital within one arm. |
| **Safety endpoint** | Incidence of any adverse events or clinically significant changes in laboratory parameters, mortality rate, neurological sequelae in patients with cerebral malaria at inclusion, or vital signs. |
| **Primary efficacy endpoint** | The proportion of patients with parasite clearance (>/= 99% reduction from the baseline asexual parasite count) at 24 hours (+/- 1 hour) after initiation of study drug. |
| **Secondary efficacy endpoints** | Time to total clearance of asexual parasites  Time to 99% reduction of asexual parasites  Time to 90% reduction of asexual parasites  Time to 50% reduction of asexual parasites  Adequate Clinical and Parasitological Response on day 28  Parasitological cure rate on day 28  Percent reduction in asexual parasites from baseline at 24 hours (+/- 1 hour) after initiation of randomized study drug  Percent reduction in asexual parasites from baseline at 48 hours after initiation of randomized study drug |
| **Pharmacokinetic endpoints** | Population pharmacokinetic studies were performed for the parent compound artesunate and the primary metabolite, dihydroartemisinin. |
