# Supplementary material for: Intramuscular Artesunate for Severe Malaria in African Children: A Multicenter Randomized Controlled Trial
Source: PLoS Med. 2016 Jan 12;13(1):e1001938. doi: 10.1371/journal.pmed.1001938 (PMC4710539; doi:10.1371/journal.pmed.1001938)
Supplement: S1 Text — (PDF) [file pmed.1001938.s009.pdf]

1.0 Title Page

CLINICAL STUDY PROTOCOL

Comparative, Open Label, Dose and Regimen Optimization Follow-up Study of Intravenous and Intramuscular Artesunate in African Children With Severe Malaria

Investigational Product:	Artesunate
Protocol Date:	February 2011
	Status: Final (Version Number: 1.0_02Feb2011)
	Dose Optimization Study
Short Protocol Title:	SMAC Artesunate Follow-Up Study
Development Phase:	
Sponsor:	Universititätsklinikum Tübingen

This study will be conducted in compliance with the protocol, Good Clinical Practice, and all other applicable regulatory requirements, including the archiving of essential documents.

Confidentiality Statement
The confidential information in this document is provided to you as an Investigator or consultant for review by you, your staff, and the applicable Independent Ethics Committee/Institutional Review Board. Your acceptance of this document constitutes agreement that you will not disclose the information contained herein to others without written authorization from Sponsors.

2.0 Protocol Approval and Authorization

Sponsor's Representative/ Sponsor's Medical Expert:

Univ.-Prof. Dr. Peter G. Kremsner
Universitätsklinikum Tübingen, Institut für Tropenmedizin

____ ________
Signature Date

Investigator Site's Representatives:

____ ________
Signature Date

3.0 Administrative Structure

Clinical Trial Institutions

<table>
<tr><th colspan="2">Coordinating Investigator</th></tr>
<tr><td colspan="2">Peter G. Kremsner, MD
Severe Malaria in African Children Network (SMAC)
Universitätsklinikum Tübingen, Institut für Tropenmedizin,
Wilhelmstraße 27
72074 Tübingen, Germany
Tel No: +497071 2987179
Fax No: +497071 295189
E-mail: peter.kremsner@uni-tuebingen.de</td></tr>
<tr><th colspan="2">Coordinating Investigator’s Team</th></tr>
<tr><td colspan="2">Carsten Köhler, PhD, MD, Biologist, Coordinator
Severe Malaria in African Children Network (SMAC)
Universitätsklinikum Tübingen, Institut für Tropenmedizin
Wilhelmstraße 27
72074 Tübingen, Germany
Tel No: +497071 2980229
Fax No: +497071 295189
E-mail: carsten.koehler@uni-tuebingen.de

Stefanie Bolte, PhD, Biologist, Project Manager
Universitätsklinikum Tübingen, Institut für Tropenmedizin
Wilhelmstraße 27
72074 Tübingen, Germany
Tel No: +497071 2986020
Fax No: +497071 295189
E-mail: stefanie.bolte@uni-tuebingen.de</td></tr>
<tr><th>Principal Investigators</th><th>Clinical Trial Centres and Affiliation</th></tr>
<tr><td>Saadou Issifou, MD
Co-PI and site representative for Lambaréné, Gabon</td><td>Albert Schweitzer Hospital,
Lambaréné, Gabon
Tel No: +24106106256
E-mail: isaadou2002@yahoo.fr</td></tr>
<tr><td>Marielle Bouyou-Akotet ep Loembe, Pr. Ag.
Co-PI and site representative for Libreville, Gabon</td><td>Département de Parasitologie Mycologie Immunologie,
Faculté de Médecine
Université des Sciences de la Santé
Libreville- Gabon
Tel: +241 06303105/ +241 703223
Email: mariellebouyou@yahoo.fr</td></tr>
</table>

Terrie Taylor, DO<br>Co-PI and site representative for Blantyre, Malawi	Queen Elizabeth Central Hospital<br>Blantyre, Malawi<br>Tel No: +265 9 969 220<br>E-mail: taylort@msu.edu
Kalifa Bojang, MD<br>Co-PI and site representative for Banjul, The Gambia	Medical Research Council Laboratories<br>Banjul, The Gambia<br>Tel No: +220 4494 072-9/4495 442-6 ext 4004<br>E-mail: kbojang@mrc.gm
Charles Newton, MD<br>Co-PI and site representative for Kilifi, Kenya	Centre for Geographic Medicine (Coast) Kenya Medical Research Institute<br>Kilifi, Kenya<br>Tel No: Tel +254 41-7522063/7222014/7522154/7525063<br>E-mail: Cnewton@kilifi.kemri-wellcome.org
Tsiri Agbenyega, MD<br>Co-PI and site representative for Kumasi, Ghana	School of Medical Sciences at the University of Science and Technology<br>Kumasi, Ghana<br>Tel No: +233 3220 64411<br>E-mail: tsiri@ghana.com
Sanjeev Krishna, MD<br>Co-PI	St. George's University of London<br>London, UK<br>Tel No: +44 208 725 5836<br>E-mail: s.krishna@sgul.ac.uk

Clinical Trial Support Structure and Responsibilities

**Study Management**	
Clinical Monitoring and Site Management	ClinaPharm, Dr. Raouf Osseni<br>Managing Director, Cotonou-BENIN
Data Monitoring Board (DMB)	Univ.-Prof. Dr. Martin P. Grobusch<br>Dept. of Internal Medicine, Div. of Infectious Diseases, Tropical Medicine and AIDS, Amsterdam Medical Center, University of Amsterdam<br><br>Univ.-Prof. Dr. Jürgen May<br>Bernhard-Nocht-Institut für Tropenmedizin Hamburg<br><br>Univ.-Prof. Dr. Markus Müller<br>Head of Department of Clinical Pharmacology, Medical University of Vienna
Data Management	CenTrial (Prof. Dr. Christoph Gleiter)
Biostatistics	Dr. Benjamin Mordmüller
Pharmacokinetic Analysis	Dr. Sanjeev Krishna
Drug Provider	Guilin Pharmaceutical (Shanghai) Co., Ltd.

	Rm. 5-O, 780 Cailun Road<br>Zhangjiang Hi-Tech Park<br>Shanghai, 201203 China<br>Tel: +86 21 5132 0317<br>Fax: +86 21 5132 0381<br>Email: xiongyj@guilinpharma.com
Funding	European and Developing Countries Clinical Trials Partnership (EDCTP), Grant #2004.01.M.d2 and Bundesministerium für Bildung und Forschung (BMBF), Germany, 01 KA 1011

4.0 Synopsis

**Study Title:**	Comparative, Open Label, Dose and Regimen Optimization Follow-up Study of Intravenous and Intramuscular Artesunate in Children with Severe Malaria
**Study Number:**	
**Regulatory Status:**	Investigational – Dose Optimization Study
**Investigational Product and Route:**	Artesunate for intravenous and intramuscular administration
**Investigators and Study Centres:**	**Coordinating Investigator:**<br>Prof. Peter G. Kremsner, MD<br>Severe Malaria in African Children Network (SMAC)<br>Institut für Tropenmedizin, Tübingen, Germany<br>**Principal Investigators:**<br>1. Saadou Issifou, MD, Albert Schweitzer Hospital, Lambaréné, Gabon<br>2. Marielle Bouyou-Akotet ep Loembe, Pr. Ag., Université de Médecine et Science de la Santé, Libreville, Gabon<br>3. Terrie Taylor, DO, Queen Elizabeth Central Hospital, Blantyre, Malawi<br>4. Bojang Kalifa, MD, Medical Research Council Laboratories, Banjul, The Gambia<br>5. Charles Newton, MD, Centre for Geographic Medicine (Coast) Kenya Medical Research Intitute, Kilifi, Kenya<br>6. Tsiri Agbenyega, MD, School of Medical Sciences at the University of Science and Technology, Kumasi, Ghana<br>7. Sanjeev Krishna, MD, St. George's University of London, UK
**Study Timelines:**	January 2011 – October 2012
**Number of Patients and Randomization:**	1044 patients will be randomized at 5 study centres (potentially involving sister sites) in Africa. Each site will be allocated initial 100 trial subjects. Subsequent allocations of 100 subjects will follow in a competitive manner upon completion of the previous slot. It will be up to the investigative centres to open additional sister sites in order to secure enrolment according to planned targets.
**Study Objectives:**	**Primary trial objective:**<br>To assess non-inferiority of iv artesunate and im artesunate simplified dosing regimens (4 mg/kg artesunate at 0, 24 and 48 hours; 12 mg/kg total dose) to the standard im treatment dosing regimen (2.4 mg/kg artesunate at 0, 12, 24, 48, 72 hours; 12 mg/kg total dose) in clearing parasitaemia in African children with severe malaria.<br><br>**Secondary trial objectives:**<br>To compare the tolerability and safety of the 3 artesunate dosing regimens.<br>To evaluate differences in the pharmacokinetic profile of parenteral artesunate by patient age and clinical presentation (total of 300 patients to be studied).<br><br>**Exploratory analysis:**<br>To assess non-invasive oto-acoustic tests linked to disease.<br>To assess predictability of fatal malaria by means of the Lambaréné-Organ-Dysfunction Score (LODS).<br>To analyze genetic polymorphisms in humans and parasites linked to disease and treatment.<br>To assess *in vitro* drug sensitivity of clinical study isolates.

**Trial Design:**	An open label, multicenter, parallel-group, three arm follow-up study to compare the antimalarial activity and safety of 3 artesunate dosing regimens in children with severe *P. falciparum* malaria: iv artesunate 4 mg/kg initially, and at 24 and 48 (12 mg/kg total dose); im artesunate 4 mg/kg initially, and at 24 and 48 hours (12 mg/kg total dose), im artesunate 2.4 mg/kg initially, and at 12, 24, 48 and 72 hours (12 mg/kg total dose). The study will also evaluate the pharmacokinetic profile of artesunate in pediatric patients. Patients will be randomized to 1 of 3 cohorts.
**Study Drug Administration:**	• Cohort 1: iv artesunate 4 mg/kg initially, and at 24 and 48 hours (12 mg/kg total dose); or<br>• Cohort 2: im artesunate 4 mg/kg initially, and at 24 and 48 hours (12 mg/kg total dose), or<br>• Cohort 3: im artesunate 2.4 mg/kg initially, and at 12, 24, 48, and 72 hours (12 mg/kg total dose).<br>As the combination of three days of artesunate with other antimalarials substantially reduced treatment failure, recrudescence and gametocyte carriage in uncomplicated paediatric malaria (Adjuik M. et al, Lancet 363, 9-17 (2004)) treatment will be completed with another antimalarial, e.g. a single dose of sulfadoxine-pyrimethamine (25 mg/kg and 1,25 mg/kg) at discharge. Other concomitant medication and supportive therapies will be given as needed and according to published guidelines (WHO TRSTMH 94 Suppl 1: 1, 2000) and standard operating procedures (SOPs) of the sites.
**Duration of Patient Participation:**	Patient participation will be for at least 28 days following the first dose of study drug. Patients will be hospitalized for at least 3 days. The patient will return to the study site for study visits on Days 7, 14, and 28.<br><br>If adverse events reported during the study are unresolved by Day 28, patients will be followed until resolution of the event or determination that no further medical management is deemed necessary. Similarly, the Investigator will instruct the patient to return to the study site if any untoward event occurs after study completion.
**Study Duration:**	Overall, it is expected that recruitment and follow-up will take approximately 1.5 years (January 2011 to October 2012).
**Inclusion Criteria:**	1. Male or female children from 6 months to 10 years.<br>2. Clinical diagnosis of severe *P. falciparum* malaria requiring hospitalization (SMAC definition: a clinical manifestation of the patient which requires hospitalization).<br>3. Parasitaemia (≥5,000 parasites/µL on initial blood smear).<br>4. Availability of child's parent/guardian and their willingness to provide written informed consent in accordance to local practice.<br>5. Willingness and ability to comply with the study protocol for the duration of the study.<br>6. Willingness to remain in the hospital for 3 days.
**Exclusion Criteria:**	1. Known serious adverse reaction or hypersensitivity to artemisinins, including artesunate, artemether, dihydroartemisinins or co-artemether (artemether/lumefantrine).<br>2. Any underlying disease that may compromise the diagnosis and the evaluation of the response to the study medication.<br>3. Participation in any investigational drug study during the 30 days prior to screening.<br>4. Adequate (according to WHO- and country specific guidelines) anti-malarial treatment within 24 hours prior to admission.

**Concomitant Treatment:**	Artesunate treatment will be completed with another antimalarial, e.g. sulfadoxine-pyrimethamine (25 mg/kg and 1,25 mg/kg) at discharge.<br>Adjunctive therapy, including fluids, glucose, and blood, will follow SMAC standards, based on WHO guidelines for the treatment of severe malaria.<br>In case of initial treatment failure with intravenous or intramuscular artesunate or a severe drug reaction to artesunate, parenteral quinine will be given to treat severe malaria, if patients had previous quinine therapy (within 12h), continue administering 10mg quinine dihydrocloride/kg every 8 hours, if no previous quinine therapy, give loading dose of 20 mg/kg and continue with normal regimen). Recurrent malarial infection within 28 days will be treated with artemether/lumefantrine.
**Stopping rules**	The following stopping rules will be applied:<br>- Failure to achieve at least 60% of patients for 99% parasite reduction at 24 hours (± 1 hour) in one arm after 100 enrolled children of this arm. In this case this arm will be stopped.<br>- Mortality: More than 5 out of 20 enrolled children die in hospital within one arm.<br>A Data Monitoring Board (DMB) will assure continuing monitoring of data in view of safety and efficacy and appropriate implementation of the defined stopping rules.
**Endpoints:**	**Primary efficacy endpoint:**<br>The proportion of patients with parasite clearance (≥99% reduction from the baseline asexual parasite count) at 24 hours (± 1 hour) after initiation of study drug.<br><br>**Secondary efficacy endpoints**:<br>Time to total clearance of asexual parasites (PC_{100})<br>Time to 99% reduction of asexual parasites (PC_{99})<br>Time to 90% reduction of asexual parasites (PC_{90})<br>Time to 50% reduction of asexual parasites (PC_{50})<br>Adequate Clinical and Parasitological Response on day 28<br>Parasitological cure rate on day 28<br>Percent reduction in asexual parasites from baseline at 24 hours (± 1 hour) after initiation of randomized study drug<br>Percent reduction in asexual parasites from baseline at 48 hours after initiation of randomized study drug<br><br>**Safety endpoints:**<br>Safety endpoints include the incidence of any adverse events or clinically significant changes in laboratory parameters, mortality rate, neurological sequelae in patients with cerebral malaria at inclusion, or vital signs.<br><br>**Pharmacokinetic endpoints:**<br>The pharmacokinetic profile of parenteral artesunate by patient age and clinical presentation will be evaluated.<br>Population pharmacokinetic (PK) studies will be performed for the parent compound artesunate and the primary metabolite, dihydroartemisinin using established population PK techniques, refined using results made available from the first dose-optimization study. We will analyze samples from 100 patients from 3 sites with population models derived from the recently completed intravenous dose-optimization study (total 300 patients to be studied).

<table>
<tr><td></td><td>Exploratory analysis:
Exploratory analysis involve results of
• the non-invasive oto-acoustic tests linked to disease
• the Lambaréné-Organ-Dysfunction Score (LODS)
• genetic polymorphisms in humans and parasites linked to disease and treatment
• in vitro drug sensitivity of parasites</td></tr>
<tr><td>Clinical and Laboratory Assessments:</td><td>Screening assessments:
• Demographic information, medical history, and prior medications.
• Physical examination.
• Clinical signs and symptoms.
• Vital signs, including height and weight.
• Clinical safety laboratory evaluations: hematology and biochemistry. Parasitological examination: thick blood films for parasite count to confirm inclusion/exclusion criteria.
• Exploratory tests:
  • non-invasive oto-acoustic test
  • Lambaréné-Organ-Dysfunction Score (LODS)
  • genetic polymorphisms in humans and parasites linked to disease and treatment
  • in vitro drug sensitivity testing
Interim assessments:
• Physical examination, clinical signs and symptoms, and vital signs throughout the study.
• Adverse event and concomitant medications.
• Clinical safety laboratory evaluations: hematology on Days 7, and, if clinically indicated, also on Day 28 and biochemistry, if clinically indicated, on Day 2, 3, 7, and 28.
• Parasitological examination: Thick blood smears will be examined at 6-hour (± 1 hour) intervals and prior to the next dose of treatment for at least 48 hours following the first dose of study drug or until <u>3 consecutive negative smears are recorded within the last 24-hour period.</u> Thick blood films will be also examined on Days 7, 14, and 28.
• Blood samples for pharmacokinetic analysis: Three samples will be obtained from each patient at randomized sampling times between Day 0 and Day 3 depending on artesunate dosing and treatment cohort.
Exploratory tests:
  • non-invasive oto-acoustic test at baseline, 12 hours after first treatment and at recovery/discharge
  • Lambaréné-Organ-Dysfunction Score (LODS) at admission and after 24 and 48 hours
  • genetic polymorphisms in humans and parasites linked to disease and treatment at baseline and as indicated by reappearance of parasites
  • in vitro drug sensitivity of re-appearing parasites</td></tr>
</table>

**Statistical Considerations**:	**Sample Size Justification:**<br>Based on results of the prior Phase II study on the simplified iv artesunate regimen (Kremsner at al, submitted) we assume 82% of patients with at least 99% parasite reduction 24 hours (± 1 hour) after the start of treatment for the 5 dose regimen as well as for the two 3 dose regimens. We assume non-inferiority with a non-inferiority margin of 10 % between the groups with two comparisons, namely the standard 5-dose regimen to the two experimental 3 dose regimens allocating equal numbers into the groups. We specify a power of 0.8 and an alpha of 0.05 with a delta of 10% for sample size calculation using the Farrington and Manning procedure (Farrington C P et al, Stat Med 9 (12), 1447-54 (1990)) as implemented in the gsDesign package of R v2.10.1. The calculated sample size is 316 per arm when multiple comparisons between the groups are allowed for. The total estimated sample size with 10 % headroom for loss to follow-up is therefore 1044 participants.<br><br>They will be allocated in subsequent slots of 100 patients per site in a competitive manner. The study will be a randomized, open label, comparative trial.<br><br>**Statistical analysis:**<br>Data will be summarized by descriptive statistics.<br><br>**Definition of study populations:**<br>• **Safety population**: All patients who receive randomized study drug. All safety analysis will be performed with this analysis population.<br>• **Intent-to-treat (ITT) population**: All patients from the safety population who have parasitologically confirmed infection with *P. falciparum* prior to treatment.<br>• **Per Protocol (PP) population**: All patients from the ITT population who receive all doses of randomized study drug until reaching the primary endpoint and did not receive rescue treatment before. This is the primary analysis population for efficacy.<br><br>**Primary endpoint analysis:**<br>The primary endpoint analysis will be performed using the PP population. The endpoint analysis will be repeated using the ITT population as sensitivity analysis of the primary endpoint analysis.

**Statistical Considerations (continued)**:	The primary efficacy endpoint is the proportion of patients with parasite clearance (≥99% reduction from the baseline asexual parasite count) at 24 hours (± 1 hour) after initiation of randomized study drug. Fisher's exact test for one-sided equivalence (Wellek et al: ISBN: 143980818X) will be used to assess treatment group differences in parasite clearance for the PP and ITT populations.<br><br>**Secondary endpoints analysis:**<br>Parasite clearance times will be summarized using Kaplan-Meier estimates and a Cox proportional hazards model. Patients for whom PC_{100} cannot be calculated will be censored at the time of the respective event (drop out, treatment failure, other).<br><br>Within 72 hours we expect very few missing values since patients are hospitalized. Therefore parasite removal will be imputed with last observation carried forward for those patients who prematurely discontinue before the scheduled evaluation.<br><br>**Safety analysis:**<br>All patients who receive any amount of study drug will be included in the safety analysis. The analysis of adverse events, laboratory data, vital signs, physical examination findings, and other safety evaluations will be performed using summary statistics.<br><br>**Pharmacokinetic analysis:**<br>Descriptive statistics for the plasma concentration data will be calculated for the set of all patients in this study who receive the full dose of artesunate and who have plasma concentration data available. Population pharmacokinetic modeling will be carried out as for the previous study, and comparisons of pharmacokinetics and pharmacodynamics made between dosing regimens.<br><br>**Exploratory analysis:**<br>Parasite metabolites in the blood will be measured to assess whole body parasite burden and the sensitivity of parasite isolates the study medication and a set of comparator drugs will be assessed on admission and in case of re-appearance.

5.0 Table of Contents

1.0 Title Page 1
2.0 Protocol Approval and Authorization 2
3.0 Administrative Structure 3
4.0 Synopsis 6
5.0 Table of Contents 12
6.0 Introduction 15
6.1 Severe Malaria 15
6.2 Treatment of Severe Malaria 15
6.3 Artesunate for Injection 17
7.0 Study Objectives 19
8.0 Investigational Plan 21
8.1 Overall Study Design and Plan: Description 21
8.2 Selection of Study Population 23
8.2.1 Inclusion Criteria 23
8.2.2 Exclusion Criteria 23
8.2.3 Prior and Concomitant Therapy 23
8.3 Antimalarial, Safety, and Pharmacokinetic Assessments/Variables 25
8.3.1 Antimalarial, Safety, and Pharmacokinetic Measurements Assessed and Flow Chart 25
8.3.2 Drug Concentration Measurement 35
8.3.3 Endpoints 35
8.3.4 Safety Endpoints 36
8.3.5 Pharmacokinetic Endpoints 36
8.3.6 Exploratory Analyzes 36
8.4 Removal of Patients from Therapy or Assessment 37
8.5 Treatments 38
8.5.1 Treatments Administered 38
8.5.2 Identity of Investigational Product 39
8.5.3 Method of Assigning Patients to Treatment Groups 40
8.5.4 Selection and Timing of Dose for Each Patient 40
8.5.5 Treatment Compliance 40
8.5.6 Drug Accountability 41
8.6 Discussion and Justification of Study Design 41
8.6.1 Discussion of Study Design and Choice of Control Groups 41

8.6.2 Appropriateness of Measurements 42
8.6.3 Selection of Doses in the Study 42
9.0 Adverse Events 42
9.1 Definitions 43
9.1.1 Adverse Event 43
9.1.2 Serious Adverse Events 44
9.2 Adverse Event Severity 45
9.3 Relationship to Study Drug 45
9.4 Action Taken and Outcome of Event 47
9.5 Adverse Event Collection Period 47
9.6 Adverse Event Reporting 48
9.7 Data Monitoring Board 50
9.7.1 Review of Serious Adverse Events and Deaths 51
9.7.2 Cohort Stopping Rules: 51
9.7.3 Study Termination Criteria: 51
10.0 Statistical Methods and Determination of Sample Size 51
10.1 General Considerations 51
10.2 Analysis Populations 52
10.3 Baseline Characteristics and Disposition 53
10.4 Efficacy Endpoints and Analyzes 53
10.4.1 Primary Efficacy Endpoint 53
10.4.2 Secondary Efficacy Endpoints 54
10.4.3 Exploratory Efficacy Endpoints 55
10.5 Exposure 56
10.6 Safety Endpoints and Analyzes 57
10.6.1 Adverse Events 57
10.6.2 Clinical Laboratory Tests 57
10.6.3 Vital Signs, Physical Findings and Other Safety Evaluations 58
10.7 Interim Analyzes 58
10.8 Determination of Sample Size 58
11.0 Ethics 59
11.1 Independent Ethics Committee (IEC) or Institutional Review Board (IRB) 59
11.2 Ethical Conduct of the Study 59
11.3 Patient Information and Consent 60
12.0 Source Documents and E-Case Report Form Completion 61

12.1 The Investigator's study file 62
12.2 Source Documents 63
12.3 E-Case Report Forms 63
12.4 Patient Confidentiality 63
13.0 Data Quality Assurance 64
14.0 Use of Information and Publication 65
15.0 Investigator's Agreement 65
16.0 Reference List 67

List of Appendices

Appendix A: List of Abbreviations and Definition of Terms
Appendix B: Severe Malaria Criteria
Appendix C: WHO Guidelines for the Treatment of Severe Malaria
Appendix D: WHO Toxicity Grading Scales
Appendix E: Declaration of Helsinki
Appendix F: Informed Consent
Appendix G: Summary of Product Character
Appendix H: Investigator Agreement

Appendix J: PK-Sampling Instructions

6.0 Introduction

6.1 Severe Malaria

Worldwide, there are an estimated 300 million malaria cases each year, which result in at least 1 million deaths annually (Breman J G et al, *Am J trop Med Hyg*. 71, 1-15 (2004)). Any patient who is unable to swallow antimalarial medications, has evidence of a vital organ dysfunction, or carries a high parasite count is at risk of dying (WHO Technical Report Series, World Health Organ Tech Rep Ser. 892, 1-74 (2000)). Severe malaria is a general term to describe advanced stages of *P. falciparum* malaria, which carries an overall mortality of approximately 15-20% (Newton C W et al, *Pharmacol Ther*. 79, 1-53 (1998)). This mortality occurs primarily in persons with minimal immunity from lack of previous infections, which in heavily endemic areas like Sub-Saharan Africa are mostly young children. The death toll due to malaria in the non-pregnant population in sub-Saharan Africa is approximately 1 million per year, and is comprised primarily of children less than 5 years of age. Among the children who survive severe malaria, approximately 10% will have easily detectable permanent developmental impairments/neurological sequelae.

Severe malaria is initially treated with parenteral medications to decrease parasitaemia rapidly to a non-life-threatening level and potentially reverse microvascular obstruction due to sequestered parasites as well as ongoing erythrocyte destruction. Rapidly decreasing peripheral parasitaemia may also reverse organ dysfunction. However, since some end-organ damage may already have occurred, and traditional antimalarial "schizonticides" do not in fact kill early schizonts in sequestered erythrocytes, rapid decreases in peripheral parasitaemia may not translate into complete reversal of organ damage. When the patient has decreased parasitaemia and is able to tolerate oral medications, parenteral therapy is stopped and remaining parasites are eliminated with oral antimalarials to achieve cure. Oral medications typically used include quinine, mefloquine, sulfadoxine/pyrimethamine, atovaquone/proguanil or oral artemisinins.

6.2 Treatment of Severe Malaria

Historically, the available parenteral agents for this use worldwide and in the US are quinine or quinidine, respectively. Quinine/quinidine kill the late ring, trophozoite, and early schizont stages of the parasite and, hence, prevents the maturation of late rings to early schizonts.

However, in spite of adequate quinine levels, *P. falciparum* will mature from early rings to late rings (which increase the sequestered parasite burden), and late schizonts will complete schizogony and liberate merozoites to infect new erythrocytes (Ter Kuile F O et al, *Experimental Parasitology* 76, 85-95 (1993)). Hence, parasitaemia may rise and symptoms worsen within the first 24 hours of therapy. Both of these agents are effective, with resultant mortality rates in severe malaria of about 20%. In Southeast Asia, the efficacy of quinine is declining. Since 1988, 78% of cerebral malaria patients were unconscious for greater than 72 hours, whereas between 1981 and 1987, 41% of patients were unconscious for that period of time (SEAQUAMAT Group, *Lancet* 366, 717-25 (2005)). Similarly, in the early 1990s, 33% of patients had parasite clearance times greater than 96 hours compared to 14% of patients in the 1980s (Pukrittayakamee S et a, *Trans R Soc Trop Med Hyg*. 88, 324-7 (1994)).

Cinchonism (tinnitus, headache, nausea, blurred vision) is the most common side effect of quinine or quinidine therapy. More severe complications of quinine therapy include blackwater fever (massive hemolysis leading to jaundice and hemoglobinuria), hyperinsulinemic hypoglycemia, and cardiotoxicity manifested by QTc prolongation or ventricular arrhythmias (Newton C W et al, *Pharmacol Ther*. 79, 1-53 (1998), Krishna S. et al, *Clinical Pharmacokinetics* 30, 263-99 (2000)). Intramuscular quinine is effective in severe malaria, but injections can cause local toxicity (Krishna S et al, *Antimicrob Agents Chemother*. 45, 1803-9 (2001)).

Artemisinin, which was part of traditional Chinese herbal medicine for centuries, was re-discovered and isolated in 1972 by Chinese scientists seeking new treatments for malaria, and reported in the literature in 1979 (Qinghaosu Antim. Coord. Group, *Chin Med J* 92, 811-6 (1979)). Dihydroartemisinin (DHA) or dihydroqinghaosu (DQHS in older literature) is the most active compound in the class used clinically, but is poorly water-soluble. Artemether and arteether (also called artemotil) are the methyl and ethyl ethers of DHA. They are oil soluble and have been marketed as intramuscular injections. Artesunate is the hemisuccinate ester of DHA, and it is water-soluble and can be administered intravenously, intramuscularly, intrarectally, or orally. The biochemical mechanism of the artemisinins has been hypothesized to involve inhibition of a parasite-encoded calcium transporter (PfATP6) and depends upon a peroxide moeity in the molecule (Eckstein-Ludwig U et al, *Nature* 424, 957-61 (2003); Haynes R K et al, *Microbes Infect*. 6, 1339-47 (2004); Krishna S et al, *Trends Mol Med*. 12, 200-5 (2006); Golenser J et al, *Int J Parasitol*. 36, 1427-41 (2006)). Artesunate's efficacy is associated with the ability to rapidly lower parasitaemia (faster than parenteral

quinine or quinidine), and it is assumed therefore to reverse more rapidly the microcirculatory defects induced by sequestered *P. falciparum* malaria and the resultant organ dysfunction (Udomsangpetch R et al, *J Infect Dis*. 173 (3), 691-8 (1996)).

6.3 Artesunate for Injection

Artesunate for injection, an initially non-International Conference on Harmonisation (ICH) Good Manufacturing Practice (GMP) parenteral (intravenous or intramuscular) formulation, has been developed and marketed by Guilin Pharmaceutical (Guangxi, China) and used in Southeast Asia and Africa over the past 10 years. After improvement of the manufacturing process this product received the WHO-prequalification by November 2010. Artesunate administered intravenously has been shown to effectively clear parasites at or above 1 mg/kg administered daily or twice on the first day. Regimens using at least 2.4 mg/kg clear parasites and resolve fever more quickly than intravenous quinine with fewer side effects. Comparative studies with parenteral quinine demonstrated statistically significant reductions in parasite clearance times, yet most studies have not shown significant differences in coma resolution times. Clinical side effects in these efficacy trials have been rare, but include rash (hives or a red rash), and possibly a higher incidence of blackwater fever. Minor side effects and laboratory abnormalities are often impossible to distinguish from the panoply of patho-physiologic effects of severe malaria. There remain insufficient data on safety and optimal dosing in children.

Intravenous artesunate has recently been associated with improved mortality outcomes in Asia as compared to the current standard-of-care, intravenous quinine. The 30% reduction in mortality was statistically significant in the adult population and subset analysis of the limited number of pediatric patients showed a non-statistically significant trend in mortality reduction with artesunate. The South East Asian Quinine Artesunate Malaria Trial (SEAQUAMAT) regimen, requiring twice daily artesunate dosing on the first day, is now the recommended dosing regimen for adults (SEAQUAMAT Group, *Lancet* 366, 717-25 (2005)), but has not been tested against regimens that are easier to administer. Additional information on the safety and pharmacokinetics of intravenous artesunate in children with severe malaria were clearly needed. In this context a large mortality study by the AQUAMAT group comparing quinine with artesunate in severe malaria in African children has also recently published. In that study a 5 dose artesunate regimen compared favorably with quinine by significantly

reducing mortality from 11% in the quinine group to 9% in the artesunate group (Dondorp A et al, *Lancet* e-pub (6 Nov 2010)).

A recent randomized, double-blind, placebo-controlled phase II trial in 197 African children, funded by European and Developing Countries Clinical Trials Partnership (EDCTP) and the Medicines for Malaria Venture (MMV), comparing the efficacy of the 5-dose regimen of intravenous artesunate (given at 0, 12, 24, 48 and 72 h) with a simplified 3-dose regimen (given at 0, 24 and 48 h), was recently performed in SMAC centres in Gabon and Malawi and focused on dose finding, simplifying dose regimens, safety, tolerability, pharmacokinetics and pharmacodynamic markers of efficacy such as parasite clearance. The primary endpoint was the proportion of children who had cleared at least 99% of their parasitaemia at 24 h compared with their baseline value. Safety data and secondary efficacy endpoints were also analyzed. The results show that both tested regimens were equally effective in parasite elimination, suggesting that the simplified once daily regimen can be considered equivalent in efficacy and safety to the conventional one. This phase II study suggests that the simplified 3-dose regimen using a cGMP-manufactured artesunate should be further studied and developed to licensure for treating severe malaria in children. Such studies should also include the intramuscular route of administration (Kremsner at al, submitted).

This underlying new trial submitted by the SMAC group builds on the results of the recently performed phase II study (Kremsner at al, submitted) will attempt to start the change in treatment practices for severe malaria in African children by the use of a simplified artesunate regimen (3-dose regimen) administered intravenously or intramuscularly.

The intramuscular route is simpler and cheaper to use, requires less skilled staff to administer and is quicker to implement than the intravenous route to manage severe malaria in children (Nealon C et al, *Antimicrob Agents Chemother*. 46, 3933-9 (2002). Indeed, our collaborative group has established the value of intramuscular quinine in severe malaria in African children to the extent that all the SMAC representatives from African centres who attended a recent meeting to review the artesunate study now ensure that im quinine is used at each of their sites (Krishna S et al, *Antimicrob Agents Chemother*. 45, 1803-9 (2001)). It has also shown in a small study that im artesunate is efficacious compared with the intravenous route and is pharmacokinetically acceptable (Nealon C et al, *Antimicrob Agents Chemother*. 46, 3933-9 (2002).

The SMAC group proposes an open three arm follow-up study to compare iv artesunate with im artesunate using the newly established regimen and comparing the two simplified regimen arms (im and iv) with the standard im treatment to generate additional data to help register and position the new GMP-formulated parenteral artesunate with the newly established regimen for iv and im treatment of severe malaria in African children within the guidelines of WHO and regulatory authorities.

Indeed many rural African settings prefer to use im versus iv administration of drugs. Therefore artesunate, whenever it will be available, will be used via im injections in practice at most sites where severe malaria is being managed. Thus it is pivotal to study this route of administration before it becomes *de facto* accepted practice to confirm its value under the rigorous conditions of a clinical trial.

1044 patients will be equally randomized in five countries in Africa, which are part of the Severe Malaria in African Children (SMAC) network. The Principal Investigator of the study is Professor Dr. Peter G. Kremsner and the Project Coordinator is Dr. Dr. Carsten Köhler from Universititätsklinikum Tübingen. The drug donor is Guilin Pharmaceutical (Shanghai) Co., Ltd. who obtained manufacturing pre-qualification from WHO on 5th November 2010 (Ref # MA051, published at http://apps.who.int/prequal)

7.0 Study Objectives

The primary objective of the study is to evaluate the efficacy of iv artesunate and im artesunate simplified dosing regimens (4 mg/kg artesunate at 0, 24, 48 hours, 12 mg/kg total dose) and the standard im treatment dosing regimen (2.4 mg/kg artesunate at 0, 12, 24, 48, 72 hours, 12 mg/kg total dose (WHO Guidelines for Treatment of Malaria, WHO, 2010) in clearing *P. falciparum* parasites in children with severe malaria through measuring the proportion of patients with parasite clearance (≥99 % reduction from the baseline asexual parasite count) at 24 hours (± 1 hour) after initiation of study drug.

Secondary efficacy endpoints include:

- Time to total clearance of asexual parasites (PC_{100})
- Time to 99% reduction of asexual parasites (PC_{99})
- Time to 90% reduction of asexual parasites (PC_{90})
- Time to 50% reduction of asexual parasites (PC_{50})

- Adequate Clinical and Parasitological Response on day 28
- Parasitological cure rate on day 28
- Percent reduction in asexual parasites from baseline at 48 hours after initiation of randomized study drug

Safety endpoints:
Safety endpoints include the incidence of any adverse events or clinically significant changes in laboratory parameters, mortality rate, neurological sequelae in patients with cerebral malaria at inclusion, or vital signs.

Pharmacokinetic endpoints:
The pharmacokinetic profile of parenteral artesunate by patient age and clinical presentation will be evaluated.
Population pharmacokinetic (PK) studies will be performed for the parent compound artesunate and the primary metabolite, dihydroartemisinin using established population PK techniques, refined using results made available from the first dose-optimization study. We will analyze samples from 300 patients from Gabon, Ghana and Kenya with population models derived from the recently completed intravenous dose-optimization study (total 300 patients to be studied).

Exploratory analysis:
Exploratory analysis involve results of

- the non-invasive oto-acoustic tests linked to disease
- the Lambaréné-Organ-Dysfunction Score (LODS)
- genetic polymorphisms in humans and parasites linked to disease and treatment
- *in vitro* drug sensitivity of parasites

8.0 Investigational Plan

8.1 Overall Study Design and Plan: Description

This is a comparative, open label, multicenter, randomized, parallel-group dose and regimen optimization follow-up study of the intravenous and intramuscular antimalarial activity and safety of 3 artesunate regimens in children with severe *P. falciparum* malaria. It will compare the efficacy, safety and tolerability of 3 dosing regimens: iv artesunate and im artesunate simplified dosing regimens (4 mg/kg artesunate at 0, 24 and 48 hours; 12 mg/kg total dose) and the standard im treatment dosing regimen (2.4 mg/kg artesunate at 0, 12, 24, 48 and 72 hours; 12 mg/kg total dose). Prior to study initiation, the protocol will be approved by the Independent Ethics Committee/Institutional Review Board(s) (IEC/IRB) of each participating site and the national regulatory authority of each study site (where applicable).

1044 patients will be randomized at 5 study centres in Africa, which are part of the Severe Malaria in African Children (SMAC) network. Patients will be randomized to 1 of 3 treatment cohorts:

Cohort 1: iv artesunate 4 mg/kg initially, and at 24 and 48 hours (12 mg/kg total dose); or

Cohort 2: im artesunate 4 mg/kg initially, and at 24 and 48 hours (12 mg/kg total dose); or

Cohort 3: im artesunate 2.4 mg/kg initially, and at 12, 24, 48, and 72 hours (12 mg/kg total dose).

The study is divided into 3 main periods including the Pre-Treatment Period (Screening/Day 0), the Treatment Period (Days 0 through 3; Day 0 is the first day of study drug dosing), and the Post-Treatment Period (including evaluations on Days 7, 14, and 28). Children presenting to the study hospitals with signs/symptoms of severe malaria will be screened for study enrollment. Those with presumed severe malaria will be identified and informed consent for participation from parents/guardians will be obtained while confirmation of malaria is determined by microscopic analysis of a thick blood smear. Patients who meet study inclusion criteria and none of the exclusion criteria will be randomized and promptly treated with 1 of the 3 artesunate regimens, while hospitalized for at least 3 days (Days 0, 1, and 2). Adjunctive therapy, including fluids, glucose, and blood, will follow SMAC standards, based

on WHO guidelines for the treatment of severe malaria (Appendix C). As soon as the patient is able to receive oral medication and no signs and symptoms of severe malaria are present, but not before the last pharmacokinetic sample is taken (approximately 74 hours after the start of therapy), treatment will be completed with another antimalarial (e.g. single dose of sulfadoxine/pyrimethamine) at discharge to ensure parasitological cure.

If the parasitaemia is controlled and the safety tests from Day 2 indicate no clinical concern warranting prolonged hospitalization, the patient may be discharged at the discretion of the Investigator. If a patient is discharged from the hospital on Day 2, he/she will return to the study site on Day 7 for evaluation. If the patient is still parasitemic and unable to tolerate oral liquids or food within 6-24 hours after the last dose of artesunate, the patient will continue to be hospitalized and treated with parenteral therapy until he/she is able to resume oral intake or a total of 7 days of therapy have been completed. All patients will return to the study site for evaluation on Days 14 and 28 to assess resolution of clinical complications and monitor for safety of therapy.

Efficacy will be assessed by various parasite clearance parameters. Safety evaluations including physical examinations, vital signs, hematology and chemistry laboratory parameters and monitoring of adverse events will be performed throughout the study. Pharmacokinetic assessments will be performed at 3 different time points during the study for 300 patients. The exploratory tests will be done as follows: oto-acoustic tests in the sites of Lambaréné, Gabon, Kumasi, Ghana and Kilifi, Kenya in a total of 200 patients without prior hearing impairment and 50 healthy children as control group at baseline, after 12 hours and at recovery/discharge; in vitro sensitivity will only be performed in Gabon at baseline; LODS at baseline and after 24 and 48 hours in all sites; genetic polymorphisms will be analyzed from the cell pellets of all PK-patients.

If adverse events reported during the study are unresolved by Day 28, patients will be followed until resolution of the event or determination that no further medical management is deemed necessary. Similarly, the Investigator will instruct the parents/guardians to return the patient to the study site if any untoward event occurs after study completion.

Patients who are withdrawn for any reason will not be replaced.

8.2 Selection of Study Population

Patients must meet all of the inclusion criteria outlined in Section 8.2.1 and none of the exclusion criteria outlined in Section 8.2.2.

8.2.1 Inclusion Criteria

1. Male or female children from 6 months up to 10 years of age
2. Clinical diagnosis of severe *P. falciparum* malaria requiring hospitalization (SMAC definition: a clinical manifestation of the patient which requires hospitalization)
3. Parasitaemia (≥5,000 parasites/µL on initial blood smear)
4. Availability of child's parent/guardian and their willingness to provide written informed consent in accordance to local practice
5. Willingness and ability to comply with the study protocol for the duration of the study
6. Willingness to remain in the hospital for 3 days

8.2.2 Exclusion Criteria

1. Known serious adverse reaction or hypersensitivity to artemisinins, including artesunate, artemether, dihydroartemisinins or co-artemether (artemether/lumefantrine)
2. Any underlying disease that may compromise the diagnosis and the evaluation of the response to the study medication
3. Participation in any investigational drug study during the 30 days prior to Screening
4. Adequate (according to WHO- and country-specific guidelines) anti-malarial treatment within 24 hours prior to admission

8.2.3 Prior and Concomitant Therapy

Any medication taken within 1 week prior to administration of study drug or during the study should be reported on the concomitant medication page of the patient's e-case report form. Relevant information, including the medication name, dates of administration, total daily dose, and route of administration as well as the indication for use, should be noted in the e-case report form.

8.2.3.1 Prohibited Medications

Patients should not have received treatment with a therapeutic dose of an antimalarial such as oral, intramuscular, or rectal artemisinin drugs or quinine within 24 hours prior to admission or during the study, or other investigational drugs within 30 days of study enrollment.

8.2.3.2 Concomitant Therapy

Concomitant medication may be used at the discretion of the patient's physician. The actual time, dose, and indication for therapy of all concomitant treatments are to be recorded in the e-case report form. Medication may be purchased locally, but medications meeting ICH cGMP standards are strongly encouraged. Patients and caregivers should be advised to avoid herbal preparations and medications. Multivitamins and/or iron may be offered at the follow-up visits. The actual time, dose, and indication for therapy of all concomitant treatments are to be recorded in the e-case report form.

The compatibility of artesunate with intravenous fluids (e.g., normal saline, 5% dextrose in water) for administration has been demonstrated.

8.2.3.3 Post-Treatment Therapy

Previous experience with artesunate alone has suggested a cure rate of approximately 50% (clearance without recrudescence of parasites by 28 days) after 72 hours of therapy, despite effective and often complete clearance of parasites from the bloodstream as detected by microscopy. This is likely due to the extremely short half-life of artesunate. Therefore, to ensure cure, an oral therapy with antimalarials (e.g. sulfadoxine/pyrimethamine; see Section 8.5.1) is administered when patients are feeling much improved and able to take oral medications.

8.2.3.4 Rescue Therapy

In case of initial treatment failure with intravenous or intramuscular artesunate or a severe drug reaction to the artesunate, parenteral quinine will be administered to treat the severe malaria. Patients with parasitologic resolution by the end of Day 3, but who are still unable to take oral therapy, will be treated immediately with appropriate follow-up therapy.

Quinine will be obtained from the hospital pharmacy and administration will follow the WHO Guidelines on the Treatment of Severe Malaria (Appendix C). The quinine dihydrochloride should be given as 10 mg/kg every 8 hours. If patients had previous quinine therapy (within

12h), continue administering of quinine dihydrochloride 10mg/kg every 8h, if no previous quinine was administered, give loading dose of 20 mg/kg and continue with normal regimen). The solution may be mixed in saline or dextrose solutions and given as a slow controlled infusion over 2-4 hours.

If malaria recurs after initial clearance it may be due to a recrudescence (incomplete cure) or re-infection with *P. falciparum*. Recurrent infection within 28 days will be treated with artemether/lumefantrine (Co-Artem) according to the manufacturer's instructions.

8.3 Antimalarial, Safety, and Pharmacokinetic Assessments/Variables

8.3.1 Antimalarial, Safety, and Pharmacokinetic Measurements Assessed and Flow Chart

A summary of study activities is presented in Table 1.

Table 1. Study Activities

Activity	Screening/ Day 0 0-24 hrs	Day 1 24-48 hrs	Day 2 48-72 hrs	Day 3 72hr+ (applies to 5-dose regimen only)	Day 7	Day 14	Day 28	Day 28+
Written Informed Consent	X							
Demographic Information and Medical History	X[a]							
Vital Signs	X[b]	X[b]	X[c]	X[c]	X	X	X	
Assessment of Clinical Signs and Symptoms	X[d]	X[d]	X[d]	X[d]	X	X	X	
Complete Physical Examination	X[a]							
Limited Physical Examination	X[e]	X[e]	X[e]	X[e]	X	X	X	
Hematology	X				X		X[f]	
Biochemistry	X		X[f]	X[f]	X[f]		X[f]	
**Parasitological Assessments**								
Blood Smears	X[g]	X[g]	X[g]	X[g]	X	X	X	
**Exploratory Analysis**								
Oto-acoustic tests[k]	X[k]	X		X				
LODS	X[l]	X	X					
Genetic polymorphisms	X[m]	X[m]	X[m]					
In vitro sensitivity	X							
Blood Sampling for Pharmacokinetic Analysis[h]	X[h]	X[h]	X[h]					
Admission to Hospital	X							
Review Inclusion/Exclusion Criteria	X							
Randomization	X							
Study Drug Administration	X	X	X	X[n]				
Administer Oral Follow-up Drug[i, j]				X				
Monitor Adverse Events	X	X	X	X	X	X	X	X
Recording of Prior and Concomitant Medication	X	X	X	X	X	X	X	
Discharge from Hospital				X				

a. Noncritical parts of the demographic/medical history information and the physical examination could be completed after therapy is started.

b. Vital signs (pulse rate, blood pressure, respiration rate, and temperature) will be assessed four-times daily and immediately prior to and approximately 15 minutes after dosing.

c. Vital signs (pulse rate, blood pressure, respiration rate, and temperature) will be assessed twice daily and immediately prior to and approximately 15 minutes after dosing.

d. Clinical signs and symptoms will be assessed twice daily.

e. Limited physical examinations will be assessed every 12 hours after the patient's first dose of study drug.

f. If clinically indicated.

g. Thick smears will be collected at 6-hour (± 1 hour) intervals after treatment and prior to the next dose of treatment for at least 48 hours following the first dose of study drug or until 3 consecutive negative smears.

h. 3 samples to be collected per patient between Day0 and Day3 depending on the cohort treatment and artesunat dosing as well as in accordance to the PK-sampling instruction in the PK-sampling appendix.

i. If patient is unable to tolerate oral medication by the end of the Day 3 and is still parasitemic, they will continue parenteral therapy until able to resume oral intake of drugs.

j. As soon as the patient is able to receive oral medication and no signs and symptoms of severe malaria are present, but not before the last pharmacokinetic sample is taken and not before parenteral artesunate therapy is completed, a single dose of sulfadoxine/pyrimethamine or another antimalarial follow-up therapy will be administered to ensure parasitological cure.

k. Oto-acoustic tests are done at baseline, 12 hours after first treatment and at recovery/discharge in 3 selected centres: Lambaréné/Gabon, Kumasi/Ghana and Kilifi/Kenya.

l. LODS is assessed at baseline after 24 and 48 hours.

m. Material for the determination of the host&parasite genetic polymorphism will be taken from all pellets of the PK-analysis.

n. Only applicable for the standard 5-dose regimen arm

8.3.1.1 Study Procedures

Written Informed Consent

Written informed consent will be obtained from the patient's parent/legally accepted representative/guardian in accordance with local practices or regulations. Additional information regarding the collection of informed consent is presented in Section 11.3.

Demographic Information and Medical History

Demographic information including age, race, and gender and a medical history will be obtained at Screening. As severe malaria is a medical emergency and the goal is to initiate therapy as fast as possible, non-critical parts of the medical history can be completed after therapy has started. The medical history will include an assessment of the duration of the clinical signs and symptoms of the current infection, whether the patient has received any prior medical treatment (all medications and supplements) for the current infection within the previous week, and any underlying condition or other clinically significant medical conditions.

Physical Examination

A physical examination will be performed at Screening; however, non-critical parts of the physical examination can be completed after therapy has started. Critical elements of the physical examination that must be completed prior to dosing include assessment of cardiovascular and respiratory status, peripheral assessment of volume status, evaluation for meningism, and assessment of the LODS. After drug administration, the physical examination should be finished.

Limited physical examinations defined by a study-specific e-case report form will be performed every 12 hours after the patient's first dose of study drug on Days 0 through 2. Limited physical examinations will also be performed on Days 7, 14, and 28. Any significant changes from Screening/Day 0 noted on physical examination will be recorded as an adverse event.

Clinical Signs and Symptoms

Investigators will be required to assess the severity of malaria twice daily on the basis of clinical signs and symptoms obtained at Screening/Day 0 through 2. Clinical signs and symptoms will also be assessed on Days 7, 14, and 28.

Vital Signs

Vital signs (pulse rate, blood pressure, respiration rate, temperature) will be assessed using the same method of collection four-times daily on Days 0 and 1 and twice daily on Days 2. Vital signs should also be measured prior to dosing and approximately 15 minutes after dosing. Significant deterioration of pulse, blood pressure and respiratory rate (in the judgment of the Investigators) should be repeated within 5 minutes to ensure reliability. The more normal of the 2 values should be reported on the e-case report form

Vital signs will also be assessed on Days 7, 14, and 28. Height and weight will be collected at Screening/Day 0.

Clinical Safety Laboratory Evaluations

Blood will be collected for clinical safety laboratory evaluations per standard operating procedures. Clinical safety laboratory evaluations (hematology and biochemistry) will be performed at Screening/Day 0 prior to dosing. Additional laboratory tests may be ordered as part of the clinical care of the patient, but will not be included in analysis except as part of adverse event monitoring.

Hematology safety laboratory evaluations will also be repeated on Day 7, and thereafter only if clinically indicated. Biochemistry safety laboratory evaluations will be repeated only if clinically indicated. If abnormal biochemistry results continue after the hospitalization period, they are to be repeated at weekly intervals until resolution or an alternative explanation to drug effect is provided. Abnormal biochemistry results are defined as values that exceed the upper limit of normal of the laboratory reference range if the patient's pre-treatment baseline value was normal, or if the pre-treatment baseline was abnormal, the upper limit of normal is defined as that pre-treatment baseline value.

An authorized laboratory technician at each study site will perform clinical safety laboratory evaluations. Laboratory reports will be reviewed and verified by the Investigator or co-Investigator. Each study site will provide a current list of normal reference laboratory values. The clinical laboratory evaluations that will be performed during the study are presented in Table 2.

Table 2. Clinical Laboratory Evaluations

Hematology	Biochemistry
Hemoglobin<br>Hematocrit<br>White blood cell count<br>White blood cell differential count<br>Red blood cell count<br>Platelet count<br>Mean corpuscular volume<br>Reticulocytes	Glucose<br>Creatinine<br>Alanine transaminase<br>Total bilirubin

Parasitological Assessments

Blood samples for parasitological assessments are to be performed using venous sampling, but malaria blood films may be collected by fingerprick, if venepuncture is not planned at a given time point. On Days 0, 1, and 2, thick smears are to be collected at 6-hour (± 1 hour) intervals after treatment and prior to the next dose of treatment for at least 48 hours following the first dose of study drug or until 3 consecutive negative smears are recorded within the last 24-hour (from time point 24 to 48 hours) period (± 1 hour).

Two smears will be made at each time interval in case one is accidentally broken or stained incorrectly. Smears should be prepared and stained with Giemsa as per the site's standard operating procedure. The slides will be stained and examined by the microscopist onsite. The main slide will then be transferred together with the backup slide (stained in a separate batch) for further evaluation and re-examination, as needed.

Microscopy Reading Paradigm

Slides will be assessed for the quality of slide preparation and staining prior to slide interpretation. Poorly prepared slides will not be read, and back-up slide will be used for interpretation. Parasite densities will be calculated based on the Lambaréné method or a count of parasites per 200 white blood cells (thick film) or per 1000 red blood cells (thin film) as per the site's standard operating procedure. A total of 200 oil immersion fields will be examined before

a blood smear is considered negative. Each slide has to be evaluated and counted by 2 microscopists onsite.

Non-concordance among microscopy interpretations is defined as disagreement about any of the following:

- Presence of asexual forms of Plasmodia on smear;
- Species of Plasmodium present on smear;
- Density of parasitaemia within a factor of 2, if either microscopist determines parasitaemia to be >100 asexual parasites/µL. If both microscopists agree as to presence and species of parasitaemia, with both counts <100/µL, the results will be considered concordant even if the reported parasitaemias are not within 2-fold of each other; or
- Parasitaemia within an absolute range of 50,000 parasites/µL. For example, a count by Microscopist A of 200,000/µL and a count by Microscopist B of 300,000/µL would not be considered concordant.

Discordant interpretation for any of the above criteria will be referred to Microscopist C (referee expert microscopist) for final slide determination. He/she will review both Smear 1 and Smear 2 to the above parameters (density will be the arithmetic mean of the 2 smears), and determine a final interpretation. For slide interpretation deemed concordant by the reviewing microscopists, the final parasite density will be the calculated arithmetic means of parasite density. The presence of only *P. falciparum* gametocytes will be considered a negative smear for asexual parasites, but gametocytes will be documented in the analysis.

For quality assurance purposes, 2% of the concordant results between Microscopist A & B will also be re-examined by the referee expert, Microscopist C. These results will not be recorded on the e-case report forms. Upon sponsor request, an external QC of a certain percentage of slides could be initiated due to which slides have to be stored clearly labeled until further notice from the sponsor.

Other TESTS for exploratory analysis (oto-acoustic emission/audiometric tests, genetic tests, LODS)

a) **Oto-acoustic emission/audiometric tests**

Reported case studies of amblyacousia (hearing loss) after cerebral malaria (Chukuezi A, Afr Health 17, 18-9 (1995)) have primarily been interpreted as related to treatment (antimalarial chemotherapeutics) (Carrara V I et al, *Malar J* 7, 233, (2008); Claessen et al, *Trop Med Int Health* 3, 482-9 (1998); Toovey S, *Travel Med Infect Dis.* 4, 71-6 (2005); Toovey S, *Trans R Soc Trop Med Hyg.* 98, 261-7 (2004)). Language disorders have been observed in African children after cerebral malaria (Carter J A et al, De*velopmental Medicine and Child Neurology* 48, 51-7 (2006); Idro R et al, Arch Dis Child. 91, 142-8 (2006); Kihara M et al, *Trop Med Internat Health.* 11 (4), 386-97 (2006)).

The observed long-term neurological sequelae of cerebral malaria (Molyneux M E et al, *Q J Med* 71, 441-59 (1989); Schmutzhard E et al, *Trans R Soc Trop Med Hyg* 78, 351-353 (1984)) can also be explained by the direct cerebral pathologies (Beare N A et al, *J Infect Dis* 199, 263-71 (2009); White V A et al, *PLoS One* 4, (ePub in 2009); Potchen M J et al, *Eur J Radiol* 74 (1), 262-8 (2010)).

Recently a high percentage of hearing impairment and inner ear pathology have been observed in a prospectively and longitudinally examined cohort of mice with severe malaria (Schmutzhard J, Malar J 9, 159-63 (2010)). In view of the prospective investigation, two different oto-acoustic tests (oto-acoustic emissions, audiometric test) will be performed at the time of malaria (Baseline, 12h after first treatment) and after recovery/discharge from hospital at 3 selected centres: Lambaréné/Gabon, Kumasi/Ghana and Kilifi/Kenya where 200 of the enrolled patients will undergo these auto acoustic tests. The 200 patients should not have any prior hearing impairment. An additional 50 healthy children will be enrolled to the oto-acoustic testing as a control group.

b) LODS (Lambaréné Organ Dysfunction Score)

The Lambaréné Organ Dysfunction Score is a simple clinical score to predict fatal malaria in African children and has been retrospectively evaluated on 23.890 African children with severe Plasmodium falciparum malaria. Clinical assessment for the LODS includes coma (BCS of ≤ 2), prostration and deep breathing. The objective is to prospectively evaluate the prognostic significance of the LODS to predict patients' outcome.

The clinical assessment will be performed on admission, after 24 and 48 hours including prostration (y/n), BCS/Coma (y/n), deep breathing (y/n).

Outcome at hospital discharge and D28 will be assessed by death, sequelae or cured (Helbok R et al, J Infect Dis. 200 (12), 1834-41 (2009)).

c)

LODS

Prostration	(0/1)
Coma	(0/1)
Deep breathing	(0/1)
Total score = sum	0-3

d) **Genetic polymorphisms**

Systematic analysis for known and novel genetic polymorphisms in humans.

We will analyze the frequency of the most important polymorphisms in human genes that have been described to influence drug metabolism. Phenotypic outliers, where an association with demethylation or glucuronidation pathways is sound (e.g. particular low/high drug/metabolite levels or drug:metabolite ratios) and which are not explained by known factors (e.g. allelic variants, compliance, dosing error) will be screened for novel variants.

Analysis of plasmodial candidate genes/markers for drug resistant malaria:

In this part we will analyze genes very likely involved in the development of drug response to artemisinins. From patients having an unusual response profile towards the administered drug or show no response at all we analyze gene alterations and the transcriptional activity in these parasites (Aragon L M, *J Antimicrob Chemother.* 57, 825-831 (2006); Daily J P et al, *Nature* 450, 1091-5 (2007); Olson K C et al, *Drug Metab Dispos.* 37, 1999-2007 (2009); Verra F et al, *Parasite Immunol.* 31, 234-53 (2009)).

Material for the determination of the host&parasite genetic polymorphism will be taken from all pellets of the PK-analysis. The sponsor will give shipment instructions.

***In vitro* sensitivity**

In vitro sensitivity against antimalarials will be assessed by HRP2-ELISA after parasite growth in the presence of ascending concentration of the respective drug. Chloroquine, artesunate and dihydroartemisinin will be included in all assays. A laboratory strain is included in the assays to serve as an internal standard. Sensitivity testing will be done on cryopreserved parasites according to standard operating procedures. The sponsor will give shipment instructions.

8.3.2 Drug Concentration Measurement

Three pharmacokinetic samples will be obtained from 300 patients from Ghana/Gabon/Kenya at randomized sampling times between Day 0 and Day 3 in dependence of treatment cohort and artesunate dosing (please refer to PK-sampling appendix J) in order to generate a population pharmacokinetic curve for artesunate and its primary metabolite, dihydroartemisinin.

Pharmacokinetic sampling times will be generated and included as part of the randomization packet for each patient. For details on PK-sample collection, processing and storage, please refer to PK-sampling appendix J. Shipment instructions will be given by the sponsor.

8.3.3 Endpoints

8.3.3.1 Primary Efficacy Endpoint

The primary efficacy endpoint is the proportion of patients with parasite clearance (≥99% reduction from the baseline asexual parasite count) at 24 hours (± 1 hour) after initiation of study drug.

8.3.3.2 Secondary Efficacy Endpoints

Secondary efficacy endpoints include:

- Time to total clearance of asexual parasites (PC_{100})
- Time to 99% reduction of asexual parasites (PC_{99})
- Time to 90% reduction of asexual parasites (PC_{90})
- Time to 50% reduction of asexual parasites (PC_{50})
- Adequate Clinical and Parasitological Response on day 28
- Parasitological cure rate on day 28
- Percent reduction in asexual parasites from baseline at 24 hours (± 1 hour) after initiation of randomized study drug

- Percent reduction in asexual parasites from baseline at 48 hours after initiation of randomized study drug

8.3.4 Safety Endpoints

Safety endpoints include the incidence of any adverse events or clinically significant changes in laboratory parameters, mortality rate, neurological sequelae in patients with cerebral malaria at inclusion, or vital signs.

8.3.5 Pharmacokinetic Endpoints

The pharmacokinetic profile of parenteral artesunate by patient age and clinical presentation will be evaluated.

Population pharmacokinetic (PK) studies will be performed for the parent compound artesunate and the primary metabolite, dihydroartemisinin using established population PK techniques, refined using results made available from the first dose-optimization study (Kremsner at al, submitted). Samples will be obtained from 100 consecutively enrolled patients from 3 selected trial centres and population models will be derived based on analysis of the recently completed intravenous dose-optimization study. A total of 300 patients will be studied, and results may also be combined with those from the previous dose optimization study to generate a larger dataset. A key aspect of pharmacokinetic analysis will be a comparison of results from intramuscular and intravenous routes of administration, in order to establish if these routes of administration yield comparable exposures to artesunate and dihydroartemisinin. Pharmacokinetic results will also be used in analysis of adverse events and pharmacodynamic endpoints such as parasite clearance kinetics and oto-acoustic endpoints. Pharmacokinetic analysis will also validate compliance assessments.

8.3.6 Exploratory Analysis

- LODS
- Genetic tests
- Oto-acoustic emission and audiometric tests

8.4 Removal of Patients from Therapy or Assessment

According to the Declaration of Helsinki (2008), patients may voluntarily withdraw from a clinical study at any given time, without prejudice to any future care or treatment (see Appendix E).

In case of early withdrawal, the Investigator will make every attempt to complete the assessments required on Day 28. As possible, assessments shall be performed prior to the administration of an alternative treatment. Reasons that can lead to premature withdrawal are listed below. Reasons for early withdrawal must be clearly documented in the patient's source documentation and transcribed into the e-case report form:

- Any severe or serious adverse event such as:

  Serious toxic or allergic reaction

  Intercurrent disease

  Aggravation of malaria or a concomitant disease (will remain in the analysis as treatment failures)

  Serious laboratory abnormality based on the WHO toxicity scale for grading adverse reaction (see Appendix D)

- Serious protocol violations.

Other reasons for early withdrawal include:

- Lack of efficacy
- Request of the patient's parent/guardian
- Investigator opinion that continuation in the study would be detrimental to the well-being of the patient
- Sponsor request

This study will be terminated if, in the opinion of the Investigator and the Sponsor, significant safety concerns arise during the conduct of the study.

8.5 Treatments

8.5.1 Treatments Administered

Patients will be randomized to 1 of 3 treatment cohorts:

Cohort 1: iv artesunate 4 mg/kg initially, and at 24 and 48 hours (12 mg/kg total dose);

or

Cohort 2: im artesunate 4 mg/kg initially, and at 24 and 48 hours (12 mg/kg total dose);

or

Cohort 3: im artesunate 2,4 mg/kg initially, and at 12, 24, 48, and 72 hours (12 mg/kg total dose).

The artesunate study medication for injection will be produced and provided by Guilin Pharmaceutical (Shanghai) Co., Ltd.; a WHO pre-qualification was obtained on 5th November 2010.

Intravenous and intramuscular artesunate will be administered as per instructions detailed in the Summary of Product Character (Appendix G).

As soon as the patient has received all doses of artesunate and is able to receive oral medication and no signs and symptoms of severe malaria are present, but not before the last pharmacokinetic sample is taken (approximately 74 hours after the start of therapy in dependence of cohort), treatment will be completed with another antimalarial, e.g. single dose of sulfadoxine/pyrimethamine at discharge (500 mg/25 mg tablets) to ensure parasitological cure. Patients are dosed by weight as presented in Table 3.

Table 3. Dosing Nomogram for Sulfadoxine/Pyrimethamine

Weight	Number of Tablets Taken as Single Dose	Dose (mg)
>45 kg	3 tablets	1500 mg/75 mg
31-45 kg	2 tablets	1000 mg/50 mg
21-30 kg	1.5 tablets	750 mg/37.5 mg
11-20 kg	1 tablet	500 mg/25 mg
5-10 kg	0.5 tablet	250 mg/12.5 mg

The dose of sulfadoxine/pyrimethamine tablets should be swallowed whole (not chewed) with plenty of liquids after a meal. Subjects should be observed for at least 1 hour after administration of the dose to ensure that it is not vomited.

8.5.2 Identity of Investigational Product

Artesunate for injection will be manufactured and released by the duly authorized pharmaceutical manufacturer Guilin Pharmaceutical (Shanghai) Co., Ltd. for use in clinical trials. Guilin's artesunate for injection obtained WHO pre-qualification on 5th November 2010.

8.5.2.1 Packaging and Labeling

Artesunate will be provided in dry-filled vials. Each vial of artesunate for injection contains 60mg artesunate sterile powder; assembled solvent contains 1 ml 5% sodium bicarbonate solution per ampoule. The vials will be labeled in accordance with applicable guidelines and regulations.

Once reconstituted, the amount of drug required for dosing will be withdrawn from the vial into a new sterile syringe, pre-labeled with the patient's identification code. A clinic staff member will record the exact time study drug was reconstituted. (see Appendix G)

8.5.2.2 Storage and Disposition of Study Drug

All drug supplies must be stored in accordance with the manufacturer's instructions. Until dispensed, the study drug vials are to be stored in a securely locked area, only accessible to authorized personnel. The dry-filled vials will be stored in a monitored refrigerator away from light sources.

The Sponsor will keep the Investigators informed of any changes to the acceptable storage temperatures, conditions and times to be observed for the investigational product (see Appendix G).

8.5.3 Method of Assigning Patients to Treatment Groups

All patients will be randomized to 1 of the 3 dosing regimens. Randomization will be balanced at each study site in a 1:1:1 ratio for each artesunate regimen. Randomization will be performed by the Sponsor as per a written randomization plan.

8.5.4 Selection and Timing of Dose for Each Patient

Each randomized patient will be administered intravenous or intramuscular artesunate according to 1 of the 3 regimens outlined below:

2.4 mg/kg im artesunate initially and at 12, 24, 48, and 72 hours

4.0 mg/kg im artesunate initially and at 24 and 48 hours

4.0 mg/kg iv artesunate initially and at 24 and 48 hours

As patients will present to the study site with life-threatening symptoms, treatment will be initiated within 1 hour after screening. The time of each dose administration will be recorded on the e-case report form. The patient's initials, the randomization number and the date dispensed will be entered on the patient's e-case report form as well as on the Drug Accountability Form. A study number must not be re-used for another patient.

8.5.5 Treatment Compliance

Study drug will be reconstituted by a pharmacy technician or trained, registered nurse, then double verified by a second study staff for proper dose calculation. Authorized study site staff will administer each dose of study drug only. The amount of drug administered will be recorded on the e-case report form.

8.5.6 Drug Accountability

Study drugs will originally be shipped from the manufacturer with a certificate of analysis and a statement of the expiration or re-test date. The manufacturer will provide the investigational product packaged to prevent contamination or deterioration during transport and storage. An import permit from the host nation will be obtained by the Investigator, if required, and sent to the Sponsor prior to drug shipment.

The Investigator must maintain all supplies used to conduct this study under adequate security. Study drug sent to the study center will be verified by the Investigator or designee; the amount sent and that the supplies were received intact will be documented by signing and dating the appropriate shipping documents. Study drug will be dispensed as per randomization list to each patient who meets the enrollment criteria. The Investigator or designee will record the patient's number, patient's initials, and date dispensed on the Drug Accountability Form.

The Investigator must maintain an accurate running accountability of study drug. At the conclusion of the study, all unused test article and unused vials can be kept at site if not expired and if drug is already registered in affected country. In case drug is not released on the market of affected country, regulatory/ethics committee approval has to be retrieved and drug is then to be used in accordance to WHO recommendations (package label description to be considered).

The Investigator agrees not to supply study drug to any patient who is not enrolled in this study or to any person not named as a co-Investigator of this study.

8.6 Discussion and Justification of Study Design

8.6.1 Discussion of Study Design and Choice of Control Groups

A randomized, three-arm parallel-group design is considered to be the most suitable study design to achieve the stated objectives for the proposed dose regimen optimization study. No placebo control was included due to ethical considerations for this vulnerable patient population. Additionally, an oral antimalarial is administered to ensure parasitological cure.

The duration of parenteral dosing selected is appropriate based on previous studies of artesunate. The 28-day follow-up period selected is commensurate with industry standards in trials for the treatment of malaria.

The endpoints and schedule of assessments selected generally follow the current WHO guidelines for surveillance of antimalarial drug efficacy. Safety assessments are according to standard industry practice and regulatory guidelines for this phase of drug development.

8.6.2 Appropriateness of Measurements

All clinical, parasitological, and laboratory assessments that will be used in this study are standard and generally accepted.

8.6.3 Selection of Doses in the Study

The doses selected for this study are considered to be safe and well tolerated based on safety and pharmacokinetic information obtained during studies of prior artesunate intravenous (and intramuscular) formulations. Artesunate is produced under standards of WHO pre-qualification and is well tolerated at doses up to 6 mg/kg.

One of the regimens selected for this study, which requires twice daily artesunate dosing on the first day, is currently the WHO recommended dosing regimen for adults. However, this regimen has not been tested against regimens that are easier to administer. Therefore, this study will compare the results of the WHO regimen to one that administers artesunate only once on the first day at a dose that is approximately double that of the WHO regimen.

9.0 Adverse Events

The Investigator will monitor each patient for clinical and laboratory evidence of adverse events on a routine basis throughout the study. The Investigator will assess and record any adverse event in detail including the date of onset, description, severity, time course, duration, seriousness and outcome, relationship of the adverse event to study drug, and any action(s) taken. Adverse events, whether in response to a query, observed by site personnel, or reported spontaneously by the patient, will be recorded.

If adverse events reported during the study are unresolved by Day 28, patients will be followed until resolution of the event, resolution with sequelae, death or unknown (only in case of Lost-to-Follow-Ups). Similarly, the Investigator will instruct the patient to return to the study site if any untoward event occurs after Day 28.

9.1 Definitions

9.1.1 Adverse Event

An adverse event is defined as any untoward medical occurrence in a patient or clinical investigation subject administered a pharmaceutical product and which does not necessarily have a causal relationship with this treatment. An adverse event can therefore be any unfavorable and unintended sign (including an abnormal laboratory finding), symptom, or disease temporally associated with the use of a medicinal (investigational) product, whether or not the event is considered causally related to the use of the product.

AE-definitions:

- Baseline Adverse Event: Any unfavourable and unintended sign, symptom, or disease occurring in the time between informed consent and dosing of the investigational medicinal product (IMP) will be regarded as baseline adverse event and documented accordingly.
- Adverse Reaction (AR): All noxious and unintended responses to a medicinal product related to any dose should be considered adverse drug reactions. The phrase responses to a medicinal product means that a causal relationship between a medicinal product and an adverse event is at least a reasonable possibility, i.e. the relationship cannot be ruled out.
- Unexpected Adverse Reaction: An adverse reaction, the nature or severity of which is not consistent with the applicable product information (e.g.: Investigator's Brochure for an unapproved investigational product or package insert/summary of product characteristics for an approved product).

- Suspected Unexpected Serious Adverse Reaction (SUSAR): A suspected adverse reaction related to an IMP (the tested IMP and comparators) which occurs in the clinical trial, and which is both unexpected and serious.

9.1.2 Serious Adverse Events

**Death of Patient**	An event that results in the death of a patient.
**Life-Threatening**	An event that, in the opinion of the Investigator, would have resulted in immediate fatality if medical intervention had not been taken. This does not include an event that would have been fatal if it had occurred in a more severe form.
**Hospitalization or Clinic Admission**	An event that results in an admission to the hospital/clinic for any length of time. This does not include an emergency room visit or admission to an outpatient facility.
**Prolongation of Hospitalization/Clinical Admission**	An event that occurs while the study patient is in the hospital/clinic and prolongs the patient's hospital/clinic stay.
**Congenital Anomaly**	An anomaly detected at or after birth, or any anomaly that results in fetal loss.
**Persistent or Significant Disability/Incapacity**	An event that results in a condition that substantially interferes with the activities of daily living of a study patient.

**Important Medical Event Requiring Medical or Surgical Intervention to Prevent Serious Outcome**	An important medical event that may not be immediately life-threatening or result in death or hospitalization, but based on medical judgment may jeopardize the patient and may require medical or surgical intervention to prevent any of the outcomes listed above (i.e., death of patient, life-threatening, hospitalization, prolongation of hospitalization, congenital anomaly, or persistent or significant disability/incapacity).

9.2 Adverse Event Severity

The Investigator will use the following definitions to rate the severity of each adverse event:

**Mild**	The adverse event is usually transient in nature and generally does not interfere with normal activities.
**Moderate**	The adverse event is sufficiently discomforting to interfere with normal activities.
**Severe**	The adverse event prevents normal activities.

9.3 Relationship to Study Drug

The relationship of adverse events to the investigational drug treatment is assessed according to the WHO Collaborating Centre for International Drug Monitoring:

Certain: A clinical event, including laboratory test abnormality, which occurs in a plausible time relationship to drug administration, and which cannot be explained by concurrent disease or other drugs or chemicals. The response to withdrawal of the drug (dechallenge) should be clinically plausible. The

event must be definite pharmacologically or phenomenologically, using a satisfactory rechallenge procedure if necessary.

Probable/Likely:

A clinical event, including laboratory test abnormality, with a reasonable time sequence to the administration of the drug, unlikely to be attributed to concurrent disease or other drugs or chemicals, and which follows a clinically reasonable response on withdrawal (dechallenge). Rechallenge information is not required to fulfil this definition.

Possible:

A clinical event, including laboratory test abnormality, with a reasonable time sequence to administration of the drug, but which could also be explained by concurrent disease or other drugs or chemicals. Information on drug withdrawal may be lacking or unclear.

Unlikely:

A clinical event, including laboratory test abnormality, with a temporal relationship to drug administration which makes a causal relationship improbable, and in which other drugs, chemicals or underlying disease provide plausible explanations.

Conditional/Unclassified:

A clinical event, including laboratory test abnormality, reported as an adverse reaction, about which more data are essential for a proper assessment, or the additional data are under examination.

Unassessable/Unclassifiable:

A report suggesting an adverse reaction, which cannot be judged because information is insufficient or contradictory, and which cannot be supplemented or verified.

9.4 Action Taken and Outcome of Event

The Investigator will use the following to describe the action(s) taken in response to an adverse event:

- No action taken
- Treatment discontinued permanently
- Reduction of the dose of the investigational drug
- Remedial drug therapy (which needs to be specified on the concomitant medication page of the e-case report form)
- Other actions taken, which need to be specified

The Investigator will follow-up on the (S)AEs until:

- Resolution
- Resolution with sequelae
- Death
- Unknown (only in case of Lost- to-Follow ups)

9.5 Adverse Event Collection Period

All adverse events reported from the time of study drug administration, whether elicited or spontaneously reported by the patient, will be collected and followed until resolution, resolution with sequelae, death or unknown (only in case of lost-to-Follow ups). In addition, serious adverse events will be collected from the time the patient signed the study-specific informed consent and followed as described above.

Adverse events occurring after discontinuation of study drug should also be recorded and monitored until resolution, resolution with sequelae, death or unknown (only in case of lost-to-Follow ups).

9.6 Adverse Event Reporting

If an adverse event meets any of the criteria from section 9.1.2, please also refer to GCP-guidelines (Guidelines for GCD, CPMP/ICH/135/95), it is to be reported to CenTrial GmbH as a serious adverse event immediately once the site has being made aware of the serious adverse event.

For all adverse events, including serious adverse events, the following must be assessed and recorded on the relevant page of the e-case report form: the date of onset, description, severity, time course, duration, seriousness and outcome, relationship of the adverse event to study drug, and any action(s) taken.

The Investigator will advise all patients participating in the study that any sign, symptom, or event that occurs either during the study or after Day 28 must be reported to the Investigator and/or other professional personnel in attendance as soon as possible so that appropriate action can be taken. Any death that occurs during the study or after discontinuation from the study must be reported as a serious adverse event. The date and cause of death will be recorded on the patient's e-case report form. A copy of the death certificate and autopsy report should be submitted when available.

In the event of a serious adverse event, the Investigator or other physician in attendance will administer therapy as indicated. Due to the need to report expedited serious adverse events to health authorities in a timely manner, the Investigator must report any serious adverse event immediately, even if the Investigator does not consider the event to be clinically significant or drug-related. Immediately after occurrence, or immediately after notice to the Investigator, all serious adverse events must be reported to the CenTrial GmbH. The immediate reporting of SAEs by the investigators is carried out by fax to:

CenTrial GmbH

Fax-number for SAE-reporting:

+49 70 71 29 45 55

For each SAE the investigator also documents an AE in the eCRF. The Investigator must notify the IEC/IRB of a serious adverse event in writing as soon as possible and in accordance with international and local laws and regulations.

The CenTrial GmbH forwards each SAE within 24 hours after receipt via fax to the sponsor (Tübingen). The sponsor as described in the safety manual will assess each SAE.

In case of a SUSAR the sponsor forwards the SAE to the sponsor's representatives in the participating country who is responsible for the SUSAR reporting according to the national legislation to the competent authority/ies, the respective leading ethics committee and the participating investigators. Details are provided in the Safety Manual.

The definitions and reporting requirements of the ICH guidelines for clinical safety data management, definitions, and standards for expedited reporting will be adhered to. Medical and scientific judgment should be exercised in deciding whether expedited reporting to the Sponsor is appropriate in other situations, such as important medical events that may not be immediately life-threatening or result in death or hospitalization but may jeopardize the patient or may require intervention to prevent one of the outcomes listed in the definitions above. These situations should usually also be considered serious.

Safety contacts:

Fax-number for SAE-reporting:

CenTrial GmbH

+49 70 71 29 45 55

Sponsor:

Prof. Dr. Peter G. Kremsner
Universitätsklinikum Tübingen, Institut für Tropenmedizin

Wilhelmstraße 27
72074 Tübingen, Germany
Tel: +49 7071 2987179
Fax: +49 7071 295189
Email: peter.kremsner@uni-tuebingen.de

Details on reporting are also provided in the Safety Manual.

9.7 Data Monitoring Board

An independent Data Monitoring Board (DMB) will be convened to review safety data from the study. The specific composition and functions of the DMB will be outlined in its charter but at a minimum will include:

- A routine review of all serious adverse events and deaths after 50 patients have completed at least 72 hours of the trial.
- Ad hoc reviews if the death rate in either cohort in this trial substantially exceeds the proscribed mortality threshold or clinically significant adverse events warrant review of specific safety data.
- After evaluation, a DMB report will be issued to the sponsor, the IRBs and the Investigators. Specific issues to be addressed include continuation of the trial enrollment or suspension of either cohort.

The DMB will receive administrative support from the sponsor.

9.7.1 Review of Serious Adverse Events and Deaths

Safety reports and enrollment figures will be collected by the Sponsor and distributed to the president of the DMB, who will monitor for death rates among patients. The anticipated death rate in children with severe malaria in these study sites is 4%, based on historical data with intravenous quinine. If the death rate in either cohort in this trial exceeds the proscribed threshold, the DMB will put on hold further enrolment pending a thorough review of serious adverse events. The DMB may wish to discuss via teleconference with the Sponsor's Medical Expert, other members of the Sponsor team and Investigator team, as appropriate. Any formal recommendation to the Sponsor to halt the trial or close enrollment in 1 of the 3 cohorts would be transmitted in accordance with the DMB Charter.

9.7.2 Cohort Stopping Rules:

The following stopping rules will be applied:

> Failure to achieve at least 60% of patients for 99% parasite reduction at 24 hours (± 1 hour) in one arm after 100 enrolled children of this arm. In this case this arm will be stopped.
>
> Mortality: More than 5 out of 20 enrolled children die in hospital within one arm.

9.7.3 Study Termination Criteria:

A Data Monitoring Board (DMB) will assure continuing monitoring of data in view of safety and efficacy and appropriate implementation of the defined stopping rules. The termination of the study will be a collaborative decision by the sponsor and the institutional review boards, based on recommendations from the DMB in their report.

10.0 Statistical Methods and Determination of Sample Size

10.1 General Considerations

This summary of statistical methods describes the general approach to analysis. A more detailed description of statistical methods will be provided by the statistical analysis. Any deviation from

the planned analysis as described here or in the statistical analysis plan will be explained in the final study report.

Non-inferiority will be tested using a 10% non-inferiority margin using an exact test procedure for one-sided equivalence of two binomial distributions with an α of 0.05. Both experimental arms (3 day regimes) are compared to the standard arm (5 day regimen). Multiple testing is accounted for by the method of Bonferroni. Comparison of the two experimental arms against each other is done only when non-inferiority is shown in the comparison against the 5-dose arm (hierarchical testing). Details are given in the statistical analysis plan.

Continuous variables will be summarized by number of non-missing observations, mean, standard deviation, minimum, median, maximum. Categorical data will be summarized by frequency and percentage of patients in each category. Percentages will be based on non-missing observations.

Unless indicated otherwise, summary statistics will be reported for observed data only. Baseline is defined as the last non-missing value prior to administration of the first dose of study drug.

10.2 Analysis Populations

Three analysis populations will be defined:

- Safety population: All patients who receive randomized study drug. All safety analysis will be performed with this analysis population.
- Intent-to-treat (ITT) population: All patients from the safety population who have parasitologically confirmed infection with *P. falciparum* prior to treatment.
- Per Protocol (PP) population: All patients from the ITT population who receive all doses of randomized study drug until reaching the primary endpoint and who did not receive rescue treatment before. This is the primary analysis population for efficacy.

10.3 Baseline Characteristics and Disposition

The number of patients randomized will be presented by treatment group, as will be the number of patients included in each analysis population. The number and percent of patients who prematurely discontinue (overall and by reason) will be summarized. Fisher's exact test will assess treatment group differences in the proportion of patients who complete the study.

Major protocol violations will be listed per patient. A major violation is any deviation that may affect the outcome of the patient.

Demographic and other baseline characteristics will be summarized descriptively for the treatment groups.

10.4 Efficacy Endpoints and Analysis

10.4.1 Primary Efficacy Endpoint

The primary efficacy endpoint is the proportion of patients with PC_{99} (≥99% reduction from the baseline asexual parasite count) at 24 hours (± 1 hour) after initiation of randomized study drug. Fisher's exact test will be used to assess treatment group differences in PC_{99} for the PP and ITT populations. 95% confidence intervals will be provided for the estimated PC_{99} rates in each treatment group and for the difference between treatment groups. The confidence intervals will be based on the continuity-corrected normal approximation to the binomial probability distribution.

For the ITT population, patients who discontinue randomized study drug and/or the study before the 24-hour evaluation will be considered to have not attained PC_{99}.

10.4.2 Secondary Efficacy Endpoints

Secondary efficacy endpoints include:

- Time to total clearance of asexual parasites (PC_{100})
- Time to 99% reduction of asexual parasites (PC_{99})
- Time to 90% reduction of asexual parasites (PC_{90})
- Time to 50% reduction of asexual parasites (PC_{50})
- Proportion of patient is genotype-uncorrected Adequate Clinical and Parasitological Response on day 28
- Proportion of patients with genotype-corrected Adequate Clinical and Parasitological Response (ACPR) on Day 28. Treatment success (ACPR) or failure will be classified according to the WHO 2005 guidelines corresponding to low- to mid-transmission areas. Genotype-corrected ACPR is defined as patients who have absence of parasitaemia on Day 28, irrespective of axillary temperature, without meeting any of the criteria of early treatment failure, late clinical failure or late parasitological failure.
- Percent reduction in asexual parasites from baseline at 24 hours (± 1 hour) after initiation of randomized study drug
- Percent reduction in asexual parasites from baseline at 48 hours after initiation of randomized study drug

Parasite clearance times will be summarized using Kaplan-Meier estimates and a Cox proportional hazards model. Patients for whom PC_{100} cannot be calculated will be censored at the time of the respective event (drop out, treatment failure, other).

The PC_{100} is defined as no asexual parasites for 3 consecutive negative readings ≥6 hours apart. Time to PC_{100} is defined as the time from the first dose of randomized study drug to the first negative reading (provided it meets the definition above).

Patients for whom PC_{100} cannot be calculated because they do not have parasite clearance by 72 hours after randomization or who are withdrawn for treatment failure before 72 hours will be

noted as having a censored PC_{100} of 72 hours. Patients who terminate early without achieving parasite clearance for reasons other than treatment failure (e.g., adverse event, patient request, intercurrent illness) will be censored at the last available parasite count.

PC_{100} will be summarized using Kaplan-Meier estimates. Treatment groups will be compared with respect to time to PC_{100} using a life-table approach. The PC_{99}, PC_{90} and PC_{50} will be similarly defined and analyzed.

Treatment group comparisons for percent reduction in asexual parasites and gametocytes from baseline at 24 hours (± 1 hour) and 48 hours (± 1 hour) after initiation of randomized study drug will be made with the Wilcoxon rank sum test. Percent reduction will be imputed with last observation carried forward for those patients who prematurely discontinue before the scheduled evaluation.

10.4.3 Exploratory Efficacy Endpoints

Exploratory efficacy endpoints of the study may include:

- Fever clearance time: The time to fever clearance time from first dosing to first normal reading of temperature (<37.5 °C) for 3 consecutive normal temperature readings ≥6 hours apart and a confirmed normal temperature 24 hours after the first normal body temperature reading.
- Proportion of patients with parasitological cure rate on Day 28.
- Selected endpoints summarized by age of the patient.
- Relationship of reduction in asexual parasites to artesunate and dihydroartemisinin plasma concentrations.

Details of the statistical analysis will be described in a statistical analysis plan.

Classification of treatment outcomes according to WHO 2005 guideline

Early Treatment Failure:

Early treatment failure is defined as severe aggravation of severe malaria on Days 1, 2, or 3, in the presence of parasitaemia and the clinician decides to give rescue treatment.

Late Clinical Failure:

- Danger signs or severe malaria in the presence of parasitaemia on any day between Day 4 and Day 28, without the subject previously meeting any of the criteria of early treatment failure;
- Axillary temperature ≥37.5 °C in the presence of parasitaemia on any day between Day 4 and Day 28, without the subject previously meeting any of the criteria of early treatment failure.

Late Parasitological Failure:

- Presence of parasitaemia between Day 4 and Day 28 with temperature <37.5 °C, without the patient previously meeting any of the criteria of early treatment failure or late clinical failure.

Adequate Clinical and Parasitological Response:

- Absence of parasitaemia on Day 28, irrespective of axillary temperature, without the patient meeting any of the criteria of early treatment failure, late clinical failure or late parasitological failure.

10.5 Exposure

The number and percent of patients will be presented by number of randomized study drug doses received. The pharmacokinetic parameters will be summarized for each treatment group using standard descriptive statistics.

10.6 Safety Endpoints and Analysis

10.6.1 Adverse Events

Adverse events will be coded using the Medical Dictionary for Regulatory Activities (MedDRA).

Incidence rates of treatment-emergent adverse events (events that started or worsened in severity after the administration of the study drug) will be summarized per treatment group as preferred term within each MedDRA system organ class. Fisher's exact test will be used to assess treatment group differences.

The incidence rate of treatment-emergent adverse events in each treatment group will be summarized by relationship to study drug (not related versus related) and severity (mild, moderate, severe). Incidence rates will be presented by time (onset ≤72 hours and onset >72 hours) if appropriate. Incidence rates will be presented by patient age if indicated.

The incidence of adverse events resulting in study drug discontinuation will be summarized. Serious adverse events will be identified and discussed as appropriate.

10.6.2 Clinical Laboratory Tests

Mean changes from baseline to Day 7, and Day 28 in biochemistry (if indicated) and hematology variables will be summarized for each treatment group. Shifts in laboratory values from baseline will be tabulated. For each laboratory parameter, each patient's baseline value will be categorized as:

- Below the lower limit of normal,
- Within the laboratory normal range, or
- Above the upper limit of normal.

Each patient's value after first dose will be similarly categorized. A cross-tabulation of patients based on these categories will be provided for each treatment group and post-randomization

evaluation day. Possibly clinically significant laboratory values will be summarized as well. No statistical testing will be performed for clinical laboratory data.

10.6.3 Vital Signs, Physical Findings and Other Safety Evaluations

Mean changes from baseline to minimum, maximum, and final vital sign values after randomization will be summarized for each treatment group. Possibly clinically significant vital signs will be summarized as well. No statistical testing will be performed for vital signs.

10.7 Interim Analysis

Periodic safety and efficacy analysis are planned with a data monitoring board performing data review unless pre-specified safety criteria are met for a review. Given that the study will be prematurely discontinued only for safety concerns, no adjustment to Type I error will be made for efficacy analysis.

10.8 Determination of Sample Size

1044 patients will be randomized in a 1:1:1 ratio to the 3 treatment groups. This sample size will provide an appropriately narrow confidence interval for the treatment difference in PC_{99}. In a previous study, 82% of children receiving WRAIR artesunate achieved PC_{99} immediately prior to the 24-hour dose of study drug.

Sample Size Justification:

Based on results of the prior Phase II study on the simplified iv artesunate regimen (Kremsner at al, submitted) we assume 82% of patients with at least 99% parasite reduction 24 hours (± 1 hour) after the start of treatment for the 5 dose regiment as well as for the two 3 dose regimens. We assume non-inferiority with a non-inferiority margin of 10 % between the groups with two comparisons, namely the standard 5 dose regimen to the two experimental 3 dose regimens allocating equal numbers into the groups. We specify a power of 0.8 and an alpha of 0.05 with a delta of 10% for sample size calculation using the Farrington and Manning[41] procedure as

implemented in the gsDesign package of R. The calculated sample size is 316 per arm when multiple comparisons between the groups are allowed for. The total estimated sample size with 10 % headroom for loss to follow-up of % therefore is 1044 participants.

This led to 295 patients per group and to allow for about 10% of patients as being unevaluable per protocol, we will include 1044 patients in total. They will be allocated to the study centres in a competitive manner in subsequent slots of 100 patients. The study will be a randomized, open label, comparative trial.

11.0 Ethics

11.1 Independent Ethics Committee (IEC) or Institutional Review Board (IRB)

The Investigator will supply documentation to the Sponsor related to the review and approval of the protocol by the IEC/IRB, and any approvals of revisions to the informed consent document or amendments to the protocol.

The Investigator will report promptly to the IEC/IRB any new information that may adversely affect the safety of patients or the conduct of the study. Similarly, the Investigator will submit written summaries of the study status to the IEC/IRB annually, or more frequently, if requested by the IEC/IRB. Upon completion of the study, the Investigator will provide the IEC/IRB with a brief report of the outcome of the study, if required.

11.2 Ethical Conduct of the Study

This study will be conducted in compliance with the study protocol. The patient's informed consent will be obtained according to the ethical principles stated in the Declaration of Helsinki (see Appendix E), the applicable guidelines for ICH Good Clinical Practice (GCP), the EU Clinical Trials Directives and the applicable laws and regulations of the participating countries.

11.3 Patient Information and Consent

Children recruited will have the diagnosis of severe malaria or moderately severe malaria. Since the patients will be presenting to the study site with life-threatening symptoms, treatment will ensue in a timely fashion in order to provide minimal risk to the patients. Due to the short time frame for the parent/guardian to make a decision to participate in this trial, the study team will carefully explain the study and the need for treatment in as unobtrusive way as possible, while initial evaluation of malaria is underway. For example:

- Assuring the subject that participation is voluntary and that if they do not want to volunteer for the study, medical treatment will NOT be withheld or delayed in any way.
- Conducting the consent process in an area with minimal interruption

Because of the life threatening nature of the studied disease the parent/guardian will initially receive an oral and simple description of the study, its requirements and risks and discomforts to their child from study personnel (nurse/physician fluent in the subject's dialect). Based on this information the parent/guardian will be asked if they are agreeing by signature in having their child participate in the study.

After start of the anti-malarial, study personnel (nurse/physician fluent in the subject's dialect) will review the informed consent document with the parent/guardian in more detail allowing time for them to ask more questions. The parent/guardian will then be allowed to review the written informed consent document in their local language and will document on this form their continued consent.

The parent/guardian will receive a copy of the signed informed consent document, and a copy will become part of the patient record.

A parent/guardian who cannot read or write will have the informed consent document explained verbally by someone who:

- Can speak the parent/guardian's dialect

- Can assess whether the parent/guardian understands what is being communicated
- Can communicate well with study staff so that questions can be answered

A witness, who is NOT a member of the study staff, will observe the verbal consent and sign that the informed consent process occurred on the informed consent document. The illiterate subject will acknowledge informed consent by fingerprint on the informed consent document.

The Investigator is responsible for ensuring that informed consent is obtained from each parent/guardian and for obtaining the appropriate signatures and dates on the informed consent document prior to the administration of study drug. Pertinent dosing information, scheduled visit dates, and instructions for contacting the Investigator will be on the consent form. Elements of the informed consent are available in Appendix F.

Appropriate forms for obtaining written informed consent and the patient information sheet will be provided by the Investigator or by the Sponsor designee.

The e-case report forms for this study will contain a section for documenting the date of informed consent and this must be completed appropriately. If new safety information results in significant changes in the risk/benefit assessment, the consent form and patient information sheet should be reviewed and updated if necessary. All parents/guardians (including those whose children are already being treated) should be informed of the new information, given a copy of the revised consent form and give their consent to continue the study.

12.0 Source Documents and E-Case Report Form Completion

The Investigator must maintain adequate and accurate records to enable the conduct of the study to be fully documented and the study data to be subsequently verified. These documents should be classified into 3 separate categories:

- The Investigator’s study file
- The patient’s clinical source documents

- e-Case report forms (electronic)

The Investigator must keep these 3 categories of documents on file for at least 15 years after completion or discontinuation of the study unless local laws require differently. After that period of time the documents may be destroyed, in accordance with local laws and regulations. Should the Investigator wish to assign the study records to another party or move them to another location, the Sponsor must be notified in advance.

If the Investigator cannot guarantee the archiving requirement at the investigational center for any or all of the documents, special arrangements must be made between the Investigator and the Sponsor to store these in a sealed container(s) away from the center so that they can be returned sealed to the Investigator in case of a regulatory audit. Where source documents are required for the continued care of the patients, appropriate copies should be made prior to storing away from the center.

The Sponsor reserves the right to terminate the study at any time. Should this be necessary, both the Sponsor and the Investigator will arrange the procedures on an individual study basis after review and consultation. In terminating the study, the Sponsor and the Investigator will assure that adequate consideration is given to the protection of the patient’s interests.

12.1 The Investigator’s study file

The Investigator’s study file (site master file) will contain the protocol, amendments if any, Investigator’s Brochure, sample of printed e-case report form and query form, IEC/IRB and governmental approval together with the relevant correspondence, patient informed consent form and patient information sheet (final version which is approved by IEC/IRB), drug records, staff *curricula vitae* and authorization forms (delegation of task), monitoring log, and other appropriate documents or correspondence.

12.2 Source Documents

Patient clinical source documents (usually defined by the project in advance to record key parameters independent of the e-case report forms) would include patient hospital/clinic or clinic records, physician's and nurse's notes, appointment book, original laboratory reports, electrocardiograms, pathology and special assessment reports, signed informed consent forms, consultant letters, and patient screening and enrollment logs.

12.3 E-Case Report Forms

Electronic case report forms will be used to transmit information collected during the study to the Sponsor and regulatory authorities, as applicable. Case report forms will be provided electronically. Patients will be identified by the patient identification number and the study identification number on all study documents.

All data on the e-case report form must be entered in accordance with the source data maintained at the site. Specific instructions on how to complete the e-case report form and how to answer the on-line queries and/or perform any changes in data already submitted will be provided by the Sponsor.

The principal Investigator will review the e-case report forms for completeness and accuracy and sign and date each set of the printed e-case report forms where indicated. The e-case report forms will be reviewed periodically for completeness, legibility and acceptability by the Sponsor (or their representatives). The Sponsor (or their representatives) will be allowed access to all source documents in order to verify e-case report form entries.

12.4 Patient Confidentiality

The first page of the e-case report form as well as all reports and communications relating to patients in the study will identify each patient only by the patient's initials (first, middle, last) and by the patient identification number. The Investigator will maintain a current confidential Patient Identification Code List of names of all patients allocated to patient identification

numbers in this study. This list will allow the Investigator to reveal the identity of the patients in the event that they need to be contacted for safety reasons. This information will be held in the strictest confidence and will only be used for emergency purposes, if needed.

13.0 Data Quality Assurance

The Sponsor, in accordance with the Guidelines for GCP (Committee for Proprietary Medicinal Products [CPMP]/ICH/135/95), is responsible for implementing and maintaining quality-assurance and quality-control systems with written standard operating procedures. Quality control will be applied to each stage of data handling.

The Sponsor's designated monitor will evaluate and document the competence of the study site (pre-study visit) and will inform the Sponsor of any problems with regards to facilities, technical equipment or medical staff. During the course of the study, the study monitor(s) will verify that informed consent was obtained from all patients, that the data are recorded correctly and completely, and that the Investigators comply with the protocol.

The study monitors are also entitled to compare the completion of the patient's e-case report form with source data and to inform the Investigator of errors or omissions. The Investigator will provide direct access to source data or documents for verification. The designated study monitor(s) will report, either in writing or by telephone contact, to the Sponsor following a study center visit.

The Sponsor will ensure supply with the investigational products and control storage conditions to Investigators. In addition, the Sponsor or its designees will provide the following:

- Instructional material to the study center, as appropriate.
- A start-up training session to instruct the Investigators and study coordinators as to the correct use of investigational products and protocol procedures. This session will provide instruction on the protocol, the completion of the e-case report form, and of the study.

In addition, a Sponsor's representative for quality assurance may audit the study. The Investigator will be informed in writing that an audit is to take place and will be given sufficient notice before the date of center-inspection.

14.0 Use of Information and Publication

All information concerning WHO pre-qualified injectable artesunate and the Sponsor's operations which is not previously published is considered confidential information.

The information developed during the conduct of this clinical study is also considered confidential and will be used by the Sponsor in connection with the study. This information may be disclosed as deemed necessary by the Sponsor. To allow for the use of the information derived from this clinical study and to ensure complete and thorough analysis, the Investigator is obligated to provide the Sponsor with complete test results and all data developed in this study.

This confidential information shall remain the sole property of the Sponsor, shall not be disclosed to others without the written consent of the Sponsor, and shall not be used except in the performance of this study.

Should the Investigator choose to publish the results of this study, a copy of the manuscript will be provided to the Sponsor at least 30 days before the date of submission to the intended publisher and permission for publication obtained in writing from the Sponsor.

15.0 Investigator's Agreement

This study is to be conducted in accordance with the standards stipulated in this protocol. The Sponsors will indemnify the Investigator and co-Investigators from all and any claims arising from this study, with the exception of claims resulting from their negligence or malpractice.

Patients are authorized all necessary medical care for injury or disease that is the result of participation in this study. Other than medical care that may be provided, patients will not

receive any additional compensation for participation in this study. This does not constitute a waiver or release of the patient's legal rights in case of study-related injury.

After reviewing the protocol, the Investigator will sign 2 protocol signature pages and return 1 of the signed pages to the Sponsor (see Appendix H).

16.0 Reference List

Adjuik M, Babiker A, Garner P, Olliaro P, Taylor W, White N. Artesunate combinations for treatment of malaria: meta-analysis. *Lancet.* 2004 Jan 3; 363 (9402): 9-17

Aragon L M, Navarro F, Heiser V, Garrigo M, Espanol M, Coll P. Rapid detection of specific gene mutations associated with isoniazid or rifampicin resistance in Mycobacterium tuberculosis clinical isolates using non-fluorescent low-density DNA microarrays. *J Antimicrob Chemother.* 2006; 57, 825-831

Beare N A, Harding S P, Taylor T E, Lewallen S, Molyneux M E. Perfusion abnormalities in children with cerebral malaria and malarial retinopathy. *J Infect Dis.* 2009; 199:263-71

Bloland P, Ringwald P, Snow R W. Assessment and monitoring of antimalarial drug efficacy for the treatment of uncomplicated P. falciparum malaria. World Health Organization. 2005

Breman J G, Alilio M S, Mills A. Conquering the intolerable burden of malaria: what's new, what's needed: a summary. *Am J Trop Med Hyg.* 2004; 71: 1-15

Carrara V I, Phyo AP, Nwee P, Soe M, Htoo H, Arunkamomkiri J, Singhasivanon P, Nosten F. Auditory assessment of patients with acute uncomplicated Plasmodium falciparum malaria treated with threee-day mefloquine-artesunate on north-western border of Thailand. *Malar J.* 2008; (6) 7:233

Carter J A, Lees JA, Gona JK, Murira G, Rimba K, Neville BG, Newton CR. Severe falciparum malaria and acquired childhood language disorder. *Developmental Medicine and Child Neurology.* 2006; 48:51-7

Chukuezi A. Hearing loss: a possible consequence of malaria. *Afr Health.* 1995; 17:18-9

Claessen F A, van Boxtel C J, Perenboom R M, Tange R A, Wetsteijn J C, Kager P A. Quinine pharmacokinetics: ototoxic and cardiotoxic effects in healthy Caucasian subjects and in patients with falciparum malaria. *Trop Med Int Health.* 1998; 3:482-9

Dondorp A. et al. AQUAMAT: an open randomised comparison of artesunate versus quinine in the treatment of severe falciparum malaria in African children. *Lancet; e-pub.* 6 Nov 2010

Daily J P, Scanfeld D, Pochet N, Le Roch K, Plouffe D, Kamal M. Distinct physiological states of Plasmodium falciparum in malaria-infected patients. *Nature.* 2007; 450:1091-5

Directive 2001/20/EC of 4 April 2001, of the European Parliament and of the Council on the approximation of the laws, regulations and administrative provisions of the Member States relating to implementation of good clinical practice in the conduct of clinical trials on medicinal products for human use

Eckstein-Ludwig U, Webb R J, Van Goethem I D, East J M, Lee A G, Kimura M, O'Neill P M, Bray P G, Ward S A, Krishna. S. Artemisinins target the SERCA of *Plasmodium falciparum*. *Nature.* 2003; 424:957–61

Farrington C P, Manning G. Test statistics and sample size formulae for binominal trials with null hypothesis of non-zero risk difference or non-unity relative risk. *Stat Med.* 1990; 9(12):1447-54

Guidelines for GCP (Committee for Proprietary Medicinal Products [CPMP]/ICH/135/95)

Golenser J, Waknine J H, Krugliak M, Hunt N H, Grau G H. Current perspectives on the mechanism of action of artemisinins. *Int J Parasitol.* 2006; 36:1427-41

Haynes R K, Krishna S. Artemisinins: activities and actions. *Microbes Infect.* 2004; 6:1339-46

Helbok R, Kendjo E, Issifou S, Lackner P, Newton C R, Kombila M, Agbenyega T, Bojang K, Dietz K, Schmutzhard E, Kremsner P G. The Lambaréné Organ Dysfunction Score (LODS) is a simple clinical predictor of fatal malaria in African children. *J Infect Dis.* 2009; 200(12): 1834-41

Idro R, Carter JA, Fegan G, Neville BG, Newton CR. Risk factors for persisting neurological and cognitive impairments following cerebral malaria. *Arch Dis Child.* 2006; 91:142-8

Kihara M, Carter J A, Newton C R. The effect of Plasmodium falciparum on cognition: a systematic review". *Trop Med Internat Health.* 2006; 11 (4): 386-97

Krishna S, White N J. Pharmacokinetics of quinine, chloroquinine and amodiaquine. Clinical implications. *Clinical Pharmacokinetics.* 1996; 30: 263-99

Krishna S, Woodrow C J, Staines H M, Haynes R K, Mercereau-Puijalon, O. Re-evaluation of how artemisinins work in light of emerging evidence of in vitro resistance. *Trends Mol Med.* 2006; 12:200-5

Krishna S, Nagaraja N V, Planche T, Agbenyega T, Bedo-Addo G, Ansong D, Owusu-Ofori A, Shroads A L, Henderson G, Hutson A, Derendorf H, Stacpoole P W. Population pharmacokinetics of intramuscular quinine in children with severe malaria. *Antimicrob Agents Chemother.* 2001; 45: 1803-9

Lackner P, Beer R, Heussler V, Goebel G, Rudzki D, Helbok R, Tannich E, Schmutzhard E. Behavioural and histopathological alterations in mice with cerebral malaria). *Neuropathol Appl Neurobiol.* 2006; 32:177-88

Molyneux M E, Taylor T E, Wirima J J, Borgstein A. Clinical features and prognostic indicatious in paeditatric cerebral malaria: a study of 131 comatose Malawia children. *Q J Med.* 1989; 71:441-59

Nealon C, Dzeing A, Muller-Romer U, Planche T, Sinou V, Kombila M, Kremsner P G, Parzy D, and Krishna S. Intramuscular Bioavailability and Clinical Efficacy of Artesunate in Gabonese Children with Severe Malaria. *Antimicrob Agents Chemother.* 2002; 46:3933-9

Newton C W, Krishna S. Severe falciparum malaria in children: current understanding of its pathophysiology and supportive treatment. *Pharmacol Ther.* 1998; 79:1-53

Olson K C, Dellinger R W, Zhong Q, Sun D, Amin S, Spratt T E et al. Functional Characterization of Missense Polymorphisms in the UDP-Glucuronosyltransferase 1A9 Gene. *Drug Metab Dispos.* 2009; 37(10) 1999-2007

Potchen M J, Birbeck G L, Demarco J K, Kampondeni S D, Beare N, Molyneux M E, Taylor T E. Neuroimaging findings in children with retinopathy confirmed cerebral malaria. *Eur J Radiol.* 2010; 74(1):262-8.

Pukrittayakamee S, Supanaranond W, Looareesuwan S, Vanijanonta S, White N J. Quinine in severe falciparum malaria: evidence of declining efficacy in Thailand. *Trans R Soc Trop Med Hyg.* 1994; 88: 324-7

Pukrittayakamee S, Chantra A, Simpson J A, Vanijanonta S, Clemens R, Looareesuwan S, White N J. Therapeutic responses to different antimalarial drugs in vivax malaria. *Antimicrob Agents Chemother.* 2000; 44(6):1680-5

Qinghaosu Antimalaria Coordinating Group. Antimalarial studies on Qinghaosu. *Chin Med J (Engl).* 1979; 92: 811-6

Schmutzhard E, Gerstenbrand F. Cerebral Malaria in Tanzania. Epidemiology, Clinical Symptoms and Neurological longterm sequelae in the light of 66 cases. *Trans R Soc Trop Med Hyg*. 1984; 78:351-353

Schmutzhard J, Kositz CH, Lackner P, Dietmann A, Fischer M, Glueckert R, Reindl M, Stephan K, Riechelmann H, Schrott-Fischer A, Schmutzhard E. Murine malaria is associated with significant hearing impairment. *Malar J.* 2010; 9:159-63.

South East Asian Quinine Artesunate Malaria Trial (SEAQUAMAT) Group. Artesunate versus quinine for treatment of severe falciparum malaria: a randomized trial. Lancet. 2005; 366:717-25

Ter Kuile F O, White N J, P. Holloway P A, Pasvol G, Krishna S. *Plasmodium falciparum*: *In vitro* studies of the pharmacodynamic properties of antimalarials used for the treatment of severe malaria. *Experimental Parasitology.* 1993; 76:85-95

Toovey S. Effects of weight, age, and time on artemether-lumefantrine associated ototoxicity and evidence of irreversibility. *Travel Med Infect Dis 2005.* 4:71-6

Toovey S, Jamieson A. Audiometric changes associated with the treatment of uncomplicated falciparum malaria with co-arthemeter. *Trans R Soc Trop Med Hyg.* 2004; 98:261-7

Udomsangpetch R, Pipitaporn B, Krishna S, Angus B, Pukrittayakamee S, Bates I, Suputtamongkol Y, Kyle D E, White N J. Antimalarial drugs reduce cytoadherence and rosetting Plasmodium falciparum. *J Infect Dis.* 1996 Mar; 173(3): 691-8

Verra F, Mangano V D, Modiano D. Genetics of susceptibility to Plasmodium falciparum: from classical malaria resistance genes towards genome-wide association studies. *Parasite Immunol.* 2009; 31:234-53

WHO Guidelines for the Treatment of Malaria. World Health Organization. 2010

White V A, Lewallen S, Beare N A, Molyneux M E, Taylor T E. Retinal pathology of pediatric cerebral malaria in Malawi. *PLoS One.* 2009; 4 (1)e4317 [Epub 2009 Jan29]

World Health Organization Technical Report Series. WHO Expert Committee on Malaria. *World Health Organ Tech Rep Ser.* 2000; 892: i–v, 1–74

WHO pre-qualification of Guilin Pharmaceutical Co., Ltd.'s Artesunate Powder for Injection (60 mg), Reference Number MA051 approved on 5 November 2010. Published at http://apps.who.int/prequal/

Appendix A. List of Abbreviations and Definition of Terms

Abbreviations

ACPR	Adequate clinical and parasitological response
CFR	Code of Federal Regulations
CPMP	Committee for Proprietary Medicinal Products
DHA	Dihydroartemisinin
DMB	Data Monitoring Board
FDA	Food and Drug Administration
GCP	Good Clinical Practice
GMP	Good Manufacturing Practice
ICH	International Conference on Harmonisation
IEC	Independent Ethics Committee
IM	Intra Muscular
IMP	Investigational Medicinal Product
IRB	Institutional Review Board
ITT	Intent-to-Treat
IV	Intra Venuous
MedDRA	Medical Dictionary for Regulatory Activities
PC_{50}	Time to 50% parasite clearance
PC_{90}	Time to 90% parasite clearance
PC_{99}	Time to 99% parasite clearance
PC_{100}	Time to 100% parasite clearance
PP	Per Protocol
SEAQUAMAT	South East Asian Quinine Artesunate Malaria Trial
SMAC	Severe Malaria in African Children
USP	United States Pharmacopeia
WHO	World Health Organization

Definition of Terms

ITT Population	All patients from the safety population who have parasitologically confirmed infection with *P. falciparum* prior to treatment.

PP Population	All patients from the ITT population who receive all doses of randomized study drug and have efficacy evaluations on Days 7 and 28 (or have reached an efficacy endpoint such as non-response, relapse, etc.). No other anti-malarial agent other than the protocol-specified treatment regimens is administered during the period from 72 hours prior to start of randomized study drug until after Day 28 unless the subject is considered a study treatment failure.
Safety Population	All patients who received randomized study drug.

Appendix B. Severe Malaria Criteria

Definition of Severe Malaria (Modified from World Health Organization 2000)

Cerebral malaria – impaired consciousness is defined as either:

- Glascow Coma Scale <11/15 (see Table 1 of Appendix B)
- Blantyre Come Scale <4/5 in pre-verbal children (see Table 2 of Appendix B) which is not attributable to any other cause (seizure within 30 minutes, hypoglycemia or sedative drugs or non-malarial cause) in a patient with *P. falciparum* malaria. Lumbar puncture is encouraged to rule out bacterial meningitis, if suspected.

Unarousable coma (the inability to localize a painful stimulus) is defined as:

- Glascow Coma Scale 9/15 or less (see Table 1 of Appendix B)
- Blantyre Come Scale 2/5 or less in pre-verbal children (see Table 2 of Appendix B)

Repeated generalized convulsions - more than 2 seizures (generalized or focal) in 24 hours despite cooling, in the presence of parasitaemia. The length, nature and number of convulsions should be recorded.

Severe anemia - anemia with a hematocrit of <15% or hemoglobin <5 g/dL with a parasite density of >10,000/µL. MCV should be recorded if at all possible.

Acidemia – arterial pH <7.35 or acidosis defined as a plasma bicarbonate level of <15 mmol/L.

Hyperlactatemia – a laboratory value of >5 mmol/L.

Circulatory collapse or shock – systolic blood pressure <50 mmHg in children or <80 mmHg in adults with clinical evidence of poor peripheral perfusion (cold, clammy, cyanotic skin).

Hyperparasitaemia – the degree of parasitaemia as an independent variable for malaria severity is dependent on age and underlying immunity. For these purposes, it is defined as:

- ≥4% parasitaemia of red blood cells (RBCs) in non-immunes living in regions of unstable endemicity
- ≥10% parasitaemia of red blood cells (RBCs) in individuals of stable endemicity

Hypoglycemia – whole blood or plasma glucose <40 mg/dL (2.2 mmol/L).

Jaundice – visible jaundice noted from examination of the sclerae and/or the mucosal surfaces of the mouth **or** with total serum bilirubin ≥3.0 mg/dL.

Acute renal failure – adults: serum creatinine ≥3.0 mg/dL (265 μmol/L) persisting after rehydration with a urine output <400 mL/24 hours; children: urine output <12 mL/kg/24 hours.

Hemoglobinuria – urine that is dark red or black with a dipstick that is positive for hemoglobin/myoglobin.

Respiratory distress – adult: visible central cyanosis or a sustained respiratory rate >32/min in an undisturbed adult; child: respiratory distress will be assessed in a child not crying or otherwise disturbed as sustained nasal flaring, indrawing of the bony structure of the lower chest wall (not just intercostals), and deep breathing (Kussmaul breathing).

Pulmonary edema – either a fluid overload syndrome with elevated central venous pressures or an adult respiratory distress syndrome; chest x-ray should be obtained if possible.

Spontaneous bleeding - bleeding from gums, nose, or gastrointestinal tract.

Disseminated intravascular coagulopathy - evidence of a consumptive coagulopathy generally associated with hemolysis as evidenced by an abnormal PT/aPTT/Fibrinogen/Thrombin Time, a haptoglobin and a D-dimer, if available.

Severe vomiting - as to impair ability to take oral antimalarials. (This is an '*impending severe malaria' indication*, and will likely constitute the most common proposed use of the drug.)

Prostration - the inability to sit in an adult or a child who is normally able to do so; usually manifested as extreme weakness, but children particularly should be encouraged to attempt to sit (in children too young to sit, it is defined as the inability to drink).

Appendix B: Table 1 Glasgow Coma Scale

		Score
Eyes open:	spontaneously<br>to speech<br>to pain<br>never	4<br>3<br>2<br>1
Best verbal response:	oriented<br>confused<br>inappropriate words<br>incomprehensible sounds<br>none	5<br>4<br>3<br>2<br>1
Best motor response:	obeys commands<br>localizes pain<br>flexion to pain<br>extension to pain<br>none	5<br>4<br>3<br>2<br>1
	Total	3–14

A state of unarousable coma is reached at a score of <10. This scale can be used repeatedly to assess improvement or deterioration.

The Blantyre Coma Scale, which was modified from the widely used Glasgow Coma Scale (1974), is applicable to children, including those who have not yet learned to speak.

Appendix B: Table 2 Blantyre Coma Scale

		Score
Best motor response:	localizes painful stimulus [a]	2
	withdraws limb from pain [b]	1
	nonspecific or absent response	0
Verbal response:	appropriate cry	2
	moan or inappropriate cry	1
	none	0
Eye movements:	directed (e.g., follows mother's face)	1
	not directed	0
Total		0–5

a Rub knuckles on patient's sternum.
b Firm pressure on thumbnail bed with horizontal pencil.

A state of unarousable coma is reached at a score of <3. This scale can be used repeatedly to assess improvement or deterioration.

Appendix C. WHO Guidelines for the Treatment of Severe Malaria:

Please follow link:

http://whqlibdoc.who.int/publications/2010/9789241547925_eng.pdf

Appendix D. WHO Toxicity Grading Scales

WHO grading as given in the attached pages:

EVALUATION OF TOXICITY

MODIFIED WHO RECOMMENDATIONS FOR GRADING OF ACUTE AND SUBACUTE TOXICITIES (COMMON TOXICITY CRITERIA)

	Grade 0	Grade 1	Grade 2	Grade 3	Grade 4
**HAEMATOLOGICAL**					
**WBC**	> 4.0	3.0 - 3.9	2.0 - 2.9	1.0 - 1.9	< 1
**PLT (1000/mm^3)**	WNL	75.0 - normal	50.0 - 74.9	25.0 - 49.9	< 25.0
**Haemoglobin** (g/100 ml)	WNL	10.0 - normal	8.0 - 10.0	6.5 - 7.9	< 6.5
(g/l)	WNL	100 - normal	80 - 100	65 - 79	< 65
(mmol/l)	WNL	6.2 - normal	4.95 - 6.2	4.0 - 4.9	< 4.0
**Granulocytes/bands** (1000/mm^3)	≥ 2.0	1.5 - 1.9	1.0 - 1.4	0.5 - 0.9	< 0.5
**Haematologic - other**	none	mild	moderate	severe	life-threatening
**HAEMORRHAGE** (clinical)	none	mild, no transfusion	gross, 1 - 2 U per episode	gross, 3 - 4 U per episode	massive, > 4 U per episode
**INFECTION**	none	mild, no active treatment	moderate, PO antibiotic	severe, IV antibiotic, anti-fungal or hospitalization	life-threatening
**GASTROINTESTINAL**					
**Nausea**	none	able to eat reasonable intake	intake significantly decreased but can eat	no significant intake	- -
**Vomiting**	none	once in 24 hrs	2-5 x in 24 hrs	6 - 10 x in 24 hrs	> 10 x in 24 hrs or requiring IV support
**Diarrhoea**	none	increase of 2 - 3 stools/day over pre-Rx	increase of 4 - 6 stools/ day, or nocturnal stools, or moderate cramping	increase of 7 - 9 stools/ day, or incontinence, or severe cramping	increase of ≥ 10 stools/ day or grossly bloody diarrhoea, or need for parenteral support
**Stomatitis**	none	painless ulcers, erythema, or mild soreness	painful erythema, oedema, or ulcers but can eat	painful erythema, oedema, or ulcers and cannot eat	requires parenteral or enteral support
**Oesophagitis/ dysphagia**	none	painless ulcers erythema, mild soreness or dysphagia	painful erythema, oedema, or ulcers or moderate dysphagia but can eat without narcotics	cannot eat solids or requires narcotics to eat	requires parenteral or enteral support or complete obstruction or perforation
**Anorexia**	none	mild	moderate	severe	life-threatening
**Gastritis/ulcer**	no	antacid	requires vigorous medical management or nonsurgical treatment	uncontrolled by medical management; requires surgery	perforation or bleeding
**Small bowel obstruction**	no	- -	intermittent, no intervention	requires intervention	requires operation
**Intestinal fistula**	no	- -	- -	yes	- -
**GI - other**	none	mild	moderate	severe	life-threatening
**OTHER MUCOSAL**	none	erythema, or mild pain not requiring treatment	patchy and serosanguinous discharge or non-narcotic for pain	confluent fibrinous mucositis or ulceration or narcotic for pain	necrosis

EVALUATION OF TOXICITY

(continued 2/5)

	Grade 0	Grade 1	Grade 2	Grade 3	Grade 4
**LIVER**					
**Bilirubin**	WNL	- -	< 1.5 x N	1.5 - 3.0 x N	> 3.0 x N
**Transaminases (SGOT/AST ; SGPT/ALT)**	WNL	≤ 2.5 x N	2.6 - 5.0 x N	5.1 - 20.0 x N	> 20.0 x N
**Alk phosphatase or 5'nucleotidase**	WNL	≤ 2.5 x N	2.6 - 5.0 x N	5.1 - 20.0 x N	> 20.0 x N
**Liver - clinical**	no change from baseline	- -	- -	precoma	hepatic coma
**Liver - other**	- -	mild	moderate	severe	life-threatening
**RENAL & BLADDER**					
**Creatinine**	WNL	<1.5 x N	1.5 - 3.0 x N	3.1 - 6.0 x N	> 6.0 x N
**Proteinuria**	no change	1+ or <0.3 g% or <3 g/l	2 - 3 + or 0.3 - 1.0 g% or 3 - 10 g/l	4 + or > 1.0 g% or > 10 g/l	nephrotic syndrome
**Haematuria**	negative	micro only	gross, no clots	gross + clots	requires transfusion
**BUN** (mg%)<br>(mmol/l)	WNL, < 20<br>WNL, < 7.5	21 - 30<br>7.6 - 10.9	31 - 50<br>11 - 18	> 50<br>> 18	- -<br>- -
**Haemorrhagic cystitis**	none	blood on microscopic examination	frank blood, no treatment required	bladder irrigation required	requires cystectomy or transfusion
**Renal failure**	- -	- -	- -	- -	dialysis required
**Incontinence**	normal	with coughing, sneezing, etc	spontaneous, some control	no control	- -
**Dysuria**	none	mild pain	painful or burning urination controlled by pyridium	not controlled by pyridium	- -
**Urinary retention**	none	residue > 100ml or occasional catheter or difficulty initiating stream	self-catheter required for voiding	surgery required (IUR or dilatation)	- -
**Increased frequency/ urgency**	no change	increase in frequency or nocturia up to 2 x normal	increase > 2 x normal but < hourly	with urgency and hourly or more or requires catheter	- -
**Bladder cramps**	none	- -	yes	- -	- -
**Ureteral obstruction**	none	unilateral, no surgery required	bilateral, no surgery required	incomplete bilateral, but stents,nephrostomy tubes or surgery needed	complete bilateral obstruction
**GU fistula**	none	- -	- -	yes	- -
**Kidney/bladder - other**	- -	mild	moderate	severe	life-threatening
**ALOPECIA**	no loss	mild hair loss	pronounced or total hair loss	- -	- -
**PULMONARY**					
**Dyspnoea**	none or no change	asymptomatic, with abnormality in PFTs	dyspnoea on significant exertion	dyspnoea at normal level of activity	dyspnoea at rest
**pO_2 / pCO_2**	no change or pO_2 > 85 and pCO_2 ≤ 40	pO_2 71-85<br>pCO_2 41-50	pO_2 61-70<br>pCO_2 51-60	pO_2 51-60<br>pCO_2 61-70	pO_2 ≤ 50 or<br>pCO_2 ≥ 71
**DLCO**	> 90% of pretreatment	76 - 90% of pretreatment	51 - 75% of pretreatment	26 - 50% of pretreatment	≤ 25% of pretreatment
**Pulmonary fibrosis**	none	radiographic changes, asymptomatic	- -	changes with symptoms	- -
**Pulmonary oedema**	none	- -	- -	radiographic changes and diuretic needed	requires intubation

EVALUATION OF TOXICITY

(continued 3/5)

	Grade 0	Grade 1	Grade 2	Grade 3	Grade 4
**PULMONARY (continued)**					
**Pneumonia (non-infectious)**	none	radiographic changes, no steroids needed	steroids required	oxygen required	assisted ventilation required
**Pleural effusion**	none	present	- -	- -	- -
**ARDS**	none	mild	moderate	severe	life-threatening
**Cough**	no change	mild, relieved by OTC medications	requires narcotic antitussive	uncontrolled cough	- -
**Pulmonary - other**	- -	mild	moderate	severe	life-threatening
**ALLERGY**	none	transient rash	urticaria, drug fever ≥ 38°C,<br>drug fever < 38°C	serum sickness,<br>mild bronchospasm<br>parenteral medication	anaphylaxis<br>bronchospasm,
**CARDIAC**					
**Cardiac dysrhythmias**	none	asymptomatic, transient, no therapy required	recurrent or persistent, no therapy required	requires treatment	requires monitoring; or hypotension or ventricular tachycardia or fibrillation
**Cardiac function**	none	asymptomatic, decline of resting LVEF ≤ 20% of baseline	asymptomatic decline of resting LVEF > 20% of baseline	mild CHF, responsive to therapy	severe or refractory CHF
**Cardiac ischaemia**	none	non-specific T wave flattening	asymptomatic ST and T wave changes for ischaemia	angina without evidence for infarction	acute myocardial infarction
**Cardiac-pericardial**	none	asymptomatic effusion, no intervention	pericarditis (rub, chest pain, ECG changes)	symptomatic effusion; drainage	tamponade; drainage urgently required
**Cardiac - other**		mild	moderate	severe	life-threatening
**Hypertension**	none or no change	asymptomatic, transient increase by > 20mm Hg (D) or to > 150/100 if previously WNL. No treatment	recurrent or persistent increase by > 20 mm Hg (D) or to >150/100 if previously WNL. No treatment	requires therapy	hypertensive crisis
**Hypotension**	none or no change	changes not requiring therapy (including transient orthostatic hypotension)	requires fluid replacement or other therapy but not hospitalisation	requires therapy and hospitalisation; resolves within 48 hrs of stopping the agent	requires therapy and hospitalisation for > 48 hrs after stopping the agent
**Phlebitis/thrombosis** (cerebral/hepatic/ **embolism** or	- -	- -	superficial phlebitis<br>(not local)	deep vein thrombosis	major event<br>pulmonary/other infarction)<br>pulmonary embolism
**Oedema**	none	1+ or dependent in evening only	2+ or dependent throughout day	3+	4+, generalized anasarca
**NEUROLOGIC**					
**Neurosensory**	none or no change	mild paraesthesias loss of deep tendon reflexes	mild or moderate objective loss; moderate paraesthesias	severe objective sensory loss or paraesthesias that interfere with function	- -
**Neuromotor**	none or no change	subjective weakness; no objective findings	mild objective weakness but no significant impairment of function	objective weakness with impairment of function	paralysis
**Neurocortical**	none	mild somnolence or agitation	moderate somnolence or agitation	severe somnolence, agitation, confusion, disorientation, hallucinations	coma, seizures, toxic psychosis
**Neurocerebellar**	none	slight incoordination dysdiadochokinesis	intention tremour, dysmetria slurred speech, nystagmus	locomotor ataxia	cerebellar necrosis
**Neuromood**	no change	mild anxiety or depression	moderate anxiety or depression	severe anxiety or depression	suicidal ideation

EVALUATION OF TOXICITY

(continued 4/5)

	Grade 0	Grade 1	Grade 2	Grade 3	Grade 4
**NEUROLOGIC (continued)**					
**Neuroheadache**	none	mild	moderate or severe but transient	unrelenting and severe	- -
**Neuroconstipation**	none or no change	mild	moderate	severe	ileus > 96 hours
**Neurohearing**	none or no change	asymptomatic, hearing loss on audiometry only	tinnitus	hearing loss interfering with function, correctable with hearing aid	deafness not correctable
**Neurovision**	none or no change	- -	- -	symptomatic subtotal loss of vision	blindness
**Pain**	none	mild	moderate	severe	intolerable
**Behavioural change**	none	change, not disruptive to patient or family	disruptive to patient or family	harmful to others or self	psychotic behavior
**Dizziness/vertigo**	none	non-disabling	- -	disabling	- -
**Taste**	normal	slightly altered taste, metallic taste	markedly altered taste	- -	- -
**Insomnia**	normal	occasional difficulty sleeping, may need pills	- -	difficulty sleeping despite medication	- -
**Neurologic (e.g. fatigue)**	mild	moderate	severe	life-threatening	
**DERMATOLOGIC**					
**Skin**	none or no change	scattered macular or papular eruption or erythema that is asymptomatic	scattered macular or macular or papular eruption or erythema with pruritus or other associated symptoms	generalized symptomatic macular, papular, or vesicular eruption	exfoliative dermatitis or ulcerating dermatitis
**Local**	none	pain	pain and swelling with inflammation or phlebitis	ulceration	plastic surgery indicated
**FLU-LIKE SYMPTOMS**					
**Fever in absence of infection**	none	37.1 - 38.0°C<br>98.7 - 100.4°F	38.1 - 40.0°C<br>100.5 - 104.0°F	> 40.0°C (104.0°F)<br>for < 24 hrs	> 40.0°C (104.0°F) for<br>> 24 hrs or with hypotension
**Chills**	none	mild or brief	pronounced or prolonged	- -	- -
**Myalgia/arthralgia**	normal	mild	decrease in ability to move	disabled	. .
**Sweats**	normal	mild and occasional	frequent or drenching	- -	- -
**Malaise**	none	mild, able to continue normal activities	impaired normal daily activity or bedrest <50% of waking hours	in bed or chair > 50% of waking hours	bed ridden or unable to care for self
**Flu-like symptoms**	- -	mild	moderate	severe	life-threatening
**WEIGHT GAIN**	< 5%	5.0 - 9.9%	10.0 - 19.9%	≥ 20%	--
**WEIGHT LOSS**	< 5%	5.0 - 9.9%	10.0 - 19.9%	≥ 20%	--
**METABOLIC**					
**Hyperglycaemia**	< 116 mg/dl<br>< 6.2 mmol/l	116 - 160<br>6.2 - 8.9	161 - 250<br>9.0 - 13.9	251 - 500<br>14.0 - 27.8	> 500 or ketoacidosis<br>> 27.8 or ketoacidosis
**Hypoglycaemia**	> 64 mg/dl<br>> 3.6 mmol/l	55 - 64<br>3.1 - 3.6	40 - 54<br>2.2 - 3.0	30 - 39<br>1.7 - 2.1	< 30<br>< 1.7
**Amylase**	WNL	< 1.5 x N	1.5 - 2.0 x N	2.1 - 5.0 x N	> 5.1 x N
**Hypercalcaemia**	< 10.6 mg/dl<br>< 2.65 mmol/l	10.6 - 11.5<br>2.65 - 2.87	11.6 - 12.5<br>2.88 - 3.12	12.6 - 13.5<br>3.13 - 3.37	≥ 13.5<br>≥ 3.37

EVALUATION OF TOXICITY

(continued 5/5)

METABOLIC (continued)

**Hypocalcaemia**	> 8.4 mg/dl<br>> 2.1 mmol/l	8.4 - 7.8<br>2.1 - 1.95	7.7 - 7.0<br>1.94 - 1.75	6.9 - 6.1<br>1.74 - 1.51	≤ 6.0<br>≤ 1.50
**Hypomagnaesia**	> 1.4 mmol/l	1.4 - 1.2	1.1 - 0.9	0.8 - 0.6	≤ 0.5
**Hyponatraemia**	WNL or > 135	131 - 135	126 - 130	121 - 125	≤ 120
**Hypokalaemia**	WNL or > 3.5	3.1 - 3.5	2.6 - 3.0	2.1 - 2.5	≤ 2.0
**Metabolic - other**	- -	mild	moderate	severe	life-threatening

COAGULATION

**Fibrinogen**	WNL	0.99 - 0.75 x N	0.74 - 0.50 x N	0.49 - 0.25 x N	≤ 0.24
**Prothrombin time**	WNL	1.01 - 1.25 x N	1.26 - 1.50 x N	1.51 - 2.00 x N	> 2.00 x N
**Partial thromboplastin time**	WNL	1.01 - 1.66 x N	1.67 - 2.33 x N	2.34 - 3.00 x N	> 3.00 x N
**Coagulation - other**		mild	moderate	severe	life-threatening

ENDOCRINE

**Impotence/libido**	normal	decrease in normal function	- -	absence of function	- -
**Sterility**	- -		yes	- -	- -
**Amenorrhoea**	no	yes	- -	- -	- -
**Gynaecomastia**	normal	mild	pronounced or painful	- -	- -
**Hot flushes**	none	mild or < 1/day	moderate and ≥ 1/day	frequent and interferes with normal function	- -
**Cushingoid**	normal	mild	pronounced	- -	- -
**Endocrine - other**	- -	mild	moderate	severe	life-threatening

EYE

**Conjunctivitis/keratitis**	none	erythema or chemosis, no steroids or antibiotics	steroids or antibiotics required	corneal ulceration or visible opacification	- -
**Dry eye**	normal	- -	requires artificial tears	- -	requires enucleation
**Glaucoma**	no change	- -	- -	yes	- -
**Eye - other**		mild	moderate	severe	life-threatening

WMA Declaration of Helsinki - Ethical Principles for Medical Research Involving Human Subjects

Adopted by the 18th WMA General Assembly, Helsinki, Finland, June 1964, and amended by the:
29th WMA General Assembly, Tokyo, Japan, October 1975
35th WMA General Assembly, Venice, Italy, October 1983
41st WMA General Assembly, Hong Kong, September 1989
48th WMA General Assembly, Somerset West, Republic of South Africa, October 1996
52nd WMA General Assembly, Edinburgh, Scotland, October 2000
53rd WMA General Assembly, Washington 2002 (Note of Clarification on paragraph 29 added)
55th WMA General Assembly, Tokyo 2004 (Note of Clarification on Paragraph 30 added)
59th WMA General Assembly, Seoul, October 2008

A. INTRODUCTION

1. The World Medical Association (WMA) has developed the Declaration of Helsinki as a statement of ethical principles for medical research involving human subjects, including research on identifiable human material and data.
The Declaration is intended to be read as a whole and each of its constituent paragraphs should not be applied without consideration of all other relevant paragraphs.

2. Although the Declaration is addressed primarily to physicians, the WMA encourages other participants in medical research involving human subjects to adopt these principles.

3. It is the duty of the physician to promote and safeguard the health of patients, including those who are involved in medical research. The physician's knowledge and conscience are dedicated to the fulfilment of this duty.

4. The Declaration of Geneva of the WMA binds the physician with the words, "The health of my patient will be my first consideration," and the International Code of Medical Ethics declares that, "A physician shall act in the patient's best interest when providing medical care."

5. Medical progress is based on research that ultimately must include studies involving human subjects. Populations that are underrepresented in medical research should be provided appropriate access to participation in research.

6. In medical research involving human subjects, the well-being of the individual research subject must take precedence over all other interests.

7. The primary purpose of medical research involving human subjects is to understand the causes, development and effects of diseases and improve preventive, diagnostic and therapeutic interventions (methods, procedures and treatments). Even the best current interventions must be evaluated continually through research for their safety, effectiveness, efficiency, accessibility and quality.

8. In medical practice and in medical research, most interventions involve risks and burdens.

9. Medical research is subject to ethical standards that promote respect for all human subjects and protect their health and rights. Some research populations are particularly vulnerable and need special protection. These include those who cannot give or refuse consent for themselves and those who may be vulnerable to coercion or undue influence.

10. Physicians should consider the ethical, legal and regulatory norms and standards for

research involving human subjects in their own countries as well as applicable international norms and standards. No national or international ethical, legal or regulatory requirement should reduce or eliminate any of the protections for research subjects set forth in this Declaration.

B. BASIC PRINCIPLES FOR ALL MEDICAL RESEARCH

11. It is the duty of physicians who participate in medical research to protect the life, health, dignity, integrity, right to self-determination, privacy, and confidentiality of personal information of research subjects.

12. Medical research involving human subjects must conform to generally accepted scientific principles, be based on a thorough knowledge of the scientific literature, other relevant sources of information, and adequate laboratory and, as appropriate, animal experimentation. The welfare of animals used for research must be respected.

13. Appropriate caution must be exercised in the conduct of medical research that may harm the environment.

14. The design and performance of each research study involving human subjects must be clearly described in a research protocol. The protocol should contain a statement of the ethical considerations involved and should indicate how the principles in this Declaration have been addressed. The protocol should include information regarding funding, sponsors, institutional affiliations, other potential conflicts of interest, incentives for subjects and provisions for treating and/or compensating subjects who are harmed as a consequence of participation in the research study. The protocol should describe arrangements for post-study access by study subjects to interventions identified as beneficial in the study or access to other appropriate care or benefits.

15. The research protocol must be submitted for consideration, comment, guidance and approval to a research ethics committee before the study begins. This committee must be independent of the researcher, the sponsor and any other undue influence. It must take into consideration the laws and regulations of the country or countries in which the research is to be performed as well as applicable international norms and standards but these must not be allowed to reduce or eliminate any of the protections for research subjects set forth in this Declaration. The committee must have the right to monitor ongoing studies. The researcher must provide monitoring information to the committee, especially information about any serious adverse events. No change to the protocol may be made without consideration and approval by the committee.

16. Medical research involving human subjects must be conducted only by individuals with the appropriate scientific training and qualifications. Research on patients or healthy volunteers requires the supervision of a competent and appropriately qualified physician or other health care professional. The responsibility for the protection of research subjects must always rest with the physician or other health care professional and never the research subjects, even though they have given consent.

17. Medical research involving a disadvantaged or vulnerable population or community is only justified if the research is responsive to the health needs and priorities of this population or community and if there is a reasonable likelihood that this population or community stands to benefit from the results of the research.

18. Every medical research study involving human subjects must be preceded by careful assessment of predictable risks and burdens to the individuals and communities involved in the research in comparison with foreseeable benefits to them and to other individuals or communities affected by the condition under investigation.

19. Every clinical trial must be registered in a publicly accessible database before recruitment of the first subject.

20. Physicians may not participate in a research study involving human subjects unless they are confident that the risks involved have been adequately assessed and can be satisfactorily managed. Physicians must immediately stop a study when the risks are found to outweigh the potential benefits or when there is conclusive proof of positive and beneficial results.

21. Medical research involving human subjects may only be conducted if the importance of the objective outweighs the inherent risks and burdens to the research subjects.

22. Participation by competent individuals as subjects in medical research must be voluntary. Although it may be appropriate to consult family members or community leaders, no competent individual may be enrolled in a research study unless he or she freely agrees.

23. Every precaution must be taken to protect the privacy of research subjects and the confidentiality of their personal information and to minimize the impact of the study on their physical, mental and social integrity.

24. In medical research involving competent human subjects, each potential subject must be adequately informed of the aims, methods, sources of funding, any possible conflicts of interest, institutional affiliations of the researcher, the anticipated benefits and potential risks of the study and the discomfort it may entail, and any other relevant aspects of the study. The potential subject must be informed of the right to refuse to participate in the study or to withdraw consent to participate at any time without reprisal. Special attention should be given to the specific information needs of individual potential subjects as well as to the methods used to deliver the information. After ensuring that the potential subject has understood the information, the physician or another appropriately qualified individual must then seek the potential subject's freely-given informed consent, preferably in writing. If the consent cannot be expressed in writing, the non-written consent must be formally documented and witnessed.

25. For medical research using identifiable human material or data, physicians must normally seek consent for the collection, analysis, storage and/or reuse. There may be situations where consent would be impossible or impractical to obtain for such research or would pose a threat to the validity of the research. In such situations the research may be done only after consideration and approval of a research ethics committee.

26. When seeking informed consent for participation in a research study the physician should be particularly cautious if the potential subject is in a dependent relationship with the physician or may consent under duress. In such situations the informed consent should be sought by an appropriately qualified individual who is completely independent of this relationship.

27. For a potential research subject who is incompetent, the physician must seek informed consent from the legally authorized representative. These individuals must not be included in a research study that has no likelihood of benefit for them unless it is intended to promote the health of the population represented by the potential subject, the research cannot instead be performed with competent persons, and the research entails only minimal risk and minimal burden.

28. When a potential research subject who is deemed incompetent is able to give assent to decisions about participation in research, the physician must seek that assent in addition to the consent of the legally authorized representative. The potential subject's dissent should be respected.

29. Research involving subjects who are physically or mentally incapable of giving consent, for example, unconscious patients, may be done only if the physical or mental condition that prevents giving informed consent is a necessary characteristic of the research population. In such circumstances the physician should seek informed consent from the legally authorized representative. If no such representative is available and if the research cannot be delayed, the study may proceed without informed consent provided that the specific reasons for involving subjects with a condition that renders them unable to give informed consent have been stated in the research protocol and the study has been approved by a research ethics committee. Consent to remain in the research should be obtained as soon as possible from the subject or a legally authorized representative.

30. Authors, editors and publishers all have ethical obligations with regard to the publication of the results of research. Authors have a duty to make publicly available the results of their research on human subjects and are accountable for the completeness and accuracy of their reports. They should adhere to accepted guidelines for ethical reporting. Negative and inconclusive as well as positive results should be published or otherwise made publicly available. Sources of funding, institutional affiliations and conflicts of interest should be declared in the publication. Reports of research not in accordance with the principles of this Declaration should not be accepted for publication.

C. ADDITIONAL PRINCIPLES FOR MEDICAL RESEARCH COMBINED WITH MEDICAL CARE

31. The physician may combine medical research with medical care only to the extent that the research is justified by its potential preventive, diagnostic or therapeutic value and if the physician has good reason to believe that participation in the research study will not adversely affect the health of the patients who serve as research subjects.

32. The benefits, risks, burdens and effectiveness of a new intervention must be tested against those of the best current proven intervention, except in the following circumstances:
The use of placebo, or no treatment, is acceptable in studies where no current proven intervention exists; or
Where for compelling and scientifically sound methodological reasons the use of placebo is necessary to determine the efficacy or safety of an intervention and the patients who receive placebo or no treatment will not be subject to any risk of serious or irreversible harm. Extreme care must be taken to avoid abuse of this option.

33. At the conclusion of the study, patients entered into the study are entitled to be informed about the outcome of the study and to share any benefits that result from it, for example, access to interventions identified as beneficial in the study or to other appropriate care or benefits.

34. The physician must fully inform the patient which aspects of the care are related to the research. The refusal of a patient to participate in a study or the patient's decision to withdraw from the study must never interfere with the patient-physician relationship.

35. In the treatment of a patient, where proven interventions do not exist or have been ineffective, the physician, after seeking expert advice, with informed consent from the patient or a legally authorized representative, may use an unproven intervention if in the physician's judgement it offers hope of saving life, re-establishing health or alleviating suffering. Where possible, this intervention should be made the object of research, designed to evaluate its safety and efficacy. In all cases, new information should be recorded and, where appropriate, made publicly available.

22.10.2008

WMA Declaration of Helsinki - Ethical Principles for Medical Research Involving

http://www.wma.net/en/30publications/10policies/b3/index.html 10.01.2011

Appendix F. Informed Consent:

INITIAL INFORMED CONSENT FORM

Title of the Study

SMAC-Artesunate Follow Up Study:

Comparative, Open Label, Dose and Regimen Optimization Follow-up Study of Intravenous and Intramuscular Artesunate in African Children With Severe Malaria

INITIAL CONSENT TO PARTICIPATE

Because of the life-threatening character of the Malaria disease and in order to allow for potential immediate treatment of my severe ill child, I consent to the participation of my child in the above mentioned clinical study based upon information provided verbally to me. I have been asked that my child participates in this study to evaluate a novel dosing scheme and novel form of administration of the effective and well tolerated Artesunate treatment of severe malaria.
The participation of my child in this study is entirely voluntary and refusal to participate will involve no penalty. In addition, my child may withdraw from the study at any time without penalty. If I (parent or legal guardian) or the sponsor chose to stop my child's participation I will be referred back to our initial examining clinician who will treat my child with any other available malaria therapy if required.

I confirm that I have also received the complete written information (FINAL CONSENT TO PARTICIPATE FORM) and that I will have the opportunity to ask questions and that all questions raised by me will be answered to my satisfaction. After receiving this information I can either reconfirm my consent to the continuing participation of my child in the study or decide at any time to stop my child's participation in the study without any penalty.

Parents/guardian's Signature____________________________

Printed Name: ________________________________

Date:____________

Thumbprint of subject if unable to sign

Witness's Signature______________________________

Printed Name: __________________________________

Date:______________________

____ ____________________

Signature of Person Giving the Consent Explanation Date

INVESTIGATOR'S SIGNATURE ________________________________

Printed Name: _______________________________ **Date:** _____________

FINAL CONSENT TO PARTICIPATE FORM

Title of the Study

<u>SMAC-Artesunate Follow Up Study:</u>

Comparative, Open Label, Dose and Regimen Optimization Follow-up Study of Intravenous and Intramuscular Artesunate in African Children With Severe Malaria

What organizations are involved?

This is a research project funded by the European and Developing Countries Clinical Trial Partnership (EDCTP) and the German Bundesministerium für Bildung und Forschung (BMBF). The Universitätsklinikum Tübingen fulfills the role of the sponsor according to ICH GCP. The investigative site responsible for the potential participation of my child in the underlying study is SITE.

Who is the Doctor in charge?

Here in SITE, the doctor in charge is NAME, title.

You are asked for participation of your child in a clinical research study to evaluate a novel dosing scheme and administration route of the effective and well-tolerated Artesunate treatment of severe malaria. The purpose of this informational document is to explain this research study and to obtain consent for the participation of your child in this study. Please take time to read this document carefully. Please request that all your questions will be answered to your satisfaction before you consent by signing the consent form.

Purpose of the Study

The purpose of the study is to find the best way of administration of the anti-malaria drug Artesunate. In the course of the study, we will compare the effect and the tolerability of 3 possible ways of giving a total dose of 12 mg/kg Artesunate:

- Five consecutive intramuscular doses of 2,4 mg/kg Artesunate
- Three consecutive intravenous doses of 4 mg/kg Artesunate
- Three consecutive intramuscular doses of 4 mg/kg Artesunate

By this comparison we want to find out whether the intramuscular administration of Artesunate is as safe and effective as the intravenous administration as well as whether three doses of 4 mg/kg Artesunate are as safe and effective as five doses of 2,4 mg/kg.

Your child will be randomly allocated to one of the three possible treatment arms as described above.

The investigational product

Artesunate is a powerful drug used to treat severe malaria, a disease that affects many people in sub-Saharan Africa. The effect and safety of Artesunate for the treatment of uncomplicated as well as severe malaria have been demonstrated in a series of clinical studies conducted in several parts of the world, including USA. Artesunate has already obtained market authorization for intravenous and intramuscular treatment of severe malaria in several African countries and has been recommended as standard therapy by WHO.

How many volunteers are going to be in the study?

A total of 1044 children with severe malaria will be enrolled.

How long does the participation of my child in the study last?

The participation of your child in the study will last for at least 28 days. During the first at least three days, your child will be hospitalized. At least three more follow-up visits to the hospital will be required thereafter.

What are the procedures, which your child will have to undergo during the study?

After the first (at least) three days of hospitalization, you must bring your child back to the clinic for follow-up examinations at least three times (on days 7, 14, and 28 after the initial dose of Artesunate). On the first day when you bring your child to the clinic (screening visit) your child will receive a physical examination and a laboratory evaluation. Based on the results of these examinations, it will be decided whether your child fulfills the criteria to be enrolled in the study.

If enrolled, your child will be required to spend at least three days as an inpatient. While in the hospital, your child will receive Artesunate at a defined dose either intravenously or intramuscularly. After completion of the defined doses of Artesunate, your child will be treated with another anti-malarial drug to ensure that all malaria parasites are cleared. Your child will then have three additional follow up visits at days 7, 14 and 28 after receiving the initial Artesunate dose.

Attached to this consent form is a schedule of study events. Please review this schedule before signing the consent form to make sure that you and your child will be available to participate for the entire duration of the study and that you will be able to bring your child to the clinic on the days required.

Study Procedures and Tests

If you choose to have your child taking part in this study you must first read this information form, discuss any questions with the clinicians and then sign the consent form. After signing this form the following steps will be taken:

- Your child's vital signs (respiratory rate, pulse, blood pressure and temperature) will be measured and recorded.
- Your child will also have a full physical examination and his/her medical history will be recorded.
- A non-invasive acoustic test will be performed on your child (only for 200 patients in Ghana, Gabon and Kenya).
- A non-invasive Lambaréné-Organ-Dysfunction Score (LODS) test will be performed on your child
- Your child will have approximately 2 ml of blood sample (about 1 teaspoon) taken that will be used for the examination of:
  - Hematology (Day 0/later only if clinically significant)
  - Biochemistry (Day 0/later only if clinically significant)
  - *In vitro* drug sensitivity (Day 0)
  - Pharmacokinetics (only for subgroup of 300 patients)
  - Genetic Polymorphisms (only for subgroup of 300 patients)
- Your child will have a few drops of blood taken from the finger to perform blood smear for the evaluation of the parasite count (consecutive blood smears every 6hs for a least 48h or until 3 parasite-negative smears)

- Your child will be randomly assigned to receive one of the three possible Artesunate treatment schemes through either an intravenous (IV) catheter placed in his/her arm or an intramuscular (IM) injection in the upper leg.

To be able to take part in the study your child must…

- be between 6 months and 10 years of age.
- have severe malaria.
- have a positive malaria blood test (only *Plasmodium falciparum*) of at least 5000 parasites per microliter.
- be willing and able to comply with the study protocol for the duration of the study.
- stay for at least 3 days in the hospital.

Your child will not be eligible for the study if he/she…

- has received any investigational drug or vaccine in the period of 30 days before entry into the study.
- has had an adequate anti-malarial treatment within 24hs prior to the admission of Artesunate.
- has any clinically significant abnormality at the screening examination that, in the opinion of the principle investigator, would place your child at increased risk when participating in this trial.
- has had serious adverse reaction or hypersensitivity to any anti-malaria drug, particularly Artemisinins.
- has another infection or neurological disease interfering with the evaluation of the study outcome.

If the results of screening show that your child cannot participate in this study, he/she and you will be informed and advised on appropriate referral to another treatment.

Medical care during the study

Your child is encouraged to return to the study clinic for evaluation at any time he/she feels sick during the study. The study sponsor will cover all medical care and pharmacy costs associated with this care.

Risks / Inconveniences

Your child will be required to travel to the clinic on several occasions for assessments.

As with any experimental drug, there is the possibility that complications and side effects, which are unknown at this time, could occur. You will be informed of any new risk(s), which may become known during the study, and to which your child may be exposed or any changes in the way the study will be done.

In case your child will receive intravenous drug application, an intravenous catheter will be placed into one of your child's veins on the day of admission. An intravenous catheter is like a flexible needle that can stay in the blood vessel for a long period of time. It would be used for blood draws and to give the Artesunate. Placing the intravenous catheter will reduce the number of times your child will be pricked with a needle. The intravenous catheter will stay in your child's vessel for at least three days he/she will be in the hospital. The risks of needle or catheter placement and blood draws may include temporary local pain, fainting, bleeding of the tissues and very rarely infection. To reduce this risk, experienced study personnel will draw blood and carry out infusions.

The main risks of Artesunate in human studies are weight loss, dizziness, nausea and diarrhea which usually disappear within 1 to 2 days.

Other side effects possibly attributed to Artesunate include rash, itching and decrease in blood counts (red and white blood cells) in some patients. A decrease in blood counts may potentially affect your child's immune system. These other side effects resolve within 7 to 14 days.

In case your child experiences any of the above side effects, adequate medical care will be provided by experienced clinical study staff. Additional care can also be received as a referral to an appropriate medical facility.

Benefits

1. All study subjects will directly benefit from a rapid diagnosis of malaria conducted by experienced clinicians.
2. Treatment with a medicine that has proven to cure severe *Plasmodium falciparum* malaria in many instances.
3. Comprehensive medical care and monitoring in an inpatient setting while undergoing malaria treatment.

Costs to Subjects

1. There are no costs to you or your child for taking part in this study. All tests and examinations and medical care are provided to your child free of charge as part of the study. The sponsor offers no other compensation.
2. You and your child will have to make yourselves available for the required study procedures. This may include time away from work to visit the clinic during the scheduled days or for further evaluation when you have abnormal labs during the study period.
3. You/your child will be refunded for the cost of travelling to the study doctor and for expenses that you/your child may have during the visit to the study site. You/your child will receive no other payment for taking part.

Voluntary Participation

Your decision to accept the participation of your child in this study is entirely voluntary and refusal to participate will involve no penalty.

In addition, your child may withdraw from the study at any time without penalty. Withdrawal from the study may mean your child will have had an incomplete course of malaria therapy. Your child will be referred back to your initial examining clinician who will treat him/her with any other available malaria therapy if required. If you choose to terminate study participation of your child, please inform the study doctor or any study personnel immediately.

The alternative to participation in the study is to continue with the health care plan developed by the hospital clinician prior to being directed to the study. The clinician's health care plan will be in accordance with the local treatment guidelines.

Precautions

If your child has experienced a significant allergic reaction to any anti-malarial medications in the past, such as skin rashes, swelling of the face and mouth and difficulty breathing, it may not be able to take part in this study. If your child has experienced any of these types of reactions, please discuss this with the study Investigator.

Investigator Responsibilities

The investigators are responsible for ensuring that the study is conducted according to accepted international Good Clinical Practice (GCP) standards, and for ensuring that the well being of study participants is always considered over all other considerations. Additionally, they are required to advise you and your child in a timely manner should any other information become available that may be relevant to your willingness to participate in the study.

Your Responsibilities
Should you agree to the participation of your child in this study, you must be prepared for him/her to undertake all study required procedures as well as all tests and follow-up visits. Should your child experience any medical problems, including suspected side effects related to the study drug, you must report these promptly to your study investigator.

Termination of Participation
Your child's participation in the study may be stopped for any of the following reasons:
1. You and/or your child decide to withdraw from the study. No reason needs to be indicated.
2. The investigator decides it is in your child's best interest to discontinue.
3. The sponsor stops the study for other reasons not currently known.

Who will have access to my files?

Information about your child's participation in this study will remain confidential. Only the hospital's staff can have access to study files and will keep all files in locked cabinets when they are not in use. Representatives of the Universitätsklinikum Tübingen, EDCTP or BMBF as part of their responsibility to oversee this research may review the information we collect. Your name and address, the name of the study and the dates of your participation will be kept in the records to make sure that you are adequately informed of risks and new information about the drug as it becomes available. The information we collect will be stored securely for a minimum of 15 years. Any report from this study will refer to you only by a study identification number and not by a name.

What will happen to your child's samples?

Your child's samples are to be used in the current study only. The samples will not be stored for future use.

Whom should you contact for information or answers to questions concerning your/your child's rights?

If during the course of this study, you have any questions about the study or need to report any injury that, you believe is related to the study, please contact the Principal Investigator NAME (Tel….). You can also contact the doctor on duty at the SITE and s/he will relay your concerns to Dr. NAME. You may contact the Principle Investigator and/or clinic at any time.

If you have any questions regarding your role and rights as a study participant, or would like to register a complaint about this study you may contact, anonymously if you wish, one or both of the following:

Ethics Committee: Local Contact to be added

Sponsor:

Universitätsklinikum Tübingen
Coordinating Investigator: Prof. Peter Kremsner (Peter.kremsner@uni-tuebingen.de)
Project Manager: Dr. Stefanie Bolte (Stefanie.bolte@uni-tuebingen.de)
Tübingen, Germany

CONSENT TO PARTICIPATE

I confirm that I have been read and have been explained the information provided in this informed consent form.

I confirm that I have had the opportunity to ask questions and all questions raised by me have been answered to my satisfaction.

I confirm that I have had time to consider the information given to me and to discuss it with others before deciding whether or not to take part in the study.

I have been given a copy of this signed Consent Form. It has been explained to me that I am free to withdraw from the study at any time without incurring any disadvantage to me in the future.

I consent to my child's participation in the study.

Parents/guardian's Signature____________________________

Printed Name: _________________________________

Date:____________

Thumbprint of subject if unable to sign

Witness's Signature_____________________________

Printed Name: __________________________________

Date:______________________

____ ____________________

Signature of Person Giving the Consent Explanation Date

INVESTIGATOR'S SIGNATURE ________________________________

Printed Name: _______________________________ **Date:** _____________

SCHEDULE OF STUDY EVENTS

VISIT	PROCEDURES
**Screening/Day 0**	▪ Take your child's temperature, heartbeat, blood pressure and breathing<br>▪ Inject Artesunate in your child's arm via catheter or intramuscular in the upper leg<br>▪ Take your child's blood from arm<br>▪ Perform non-invasive oto-acoustic test (0hs&12hs, only in Ghana, Gabon and Kenya)<br>▪ Perform non-invasive LODS<br>▪ Take a few drops of your child's blood from the finger*
**Day 1**	▪ Inject Artesunate in your child's arm via catheter or intramuscular in the upper leg<br>▪ Take your child's temperature, heartbeat, blood pressure and breathing.<br>▪ Perform non-invasive LODS<br>▪ Take a few drops of your child's blood from the finger*<br>▪ If clinically indicated, take blood from your child's arm
**Day 2**	▪ Inject Artesunate in your child's arm via catheter or intramuscular in the upper leg<br>▪ Take your child's temperature, heartbeat, blood pressure and breathing.<br>▪ Perform non-invasive LODS<br>▪ Take a few drops of your child's blood from the finger*<br>▪ If clinically indicated, take blood from your child's arm
**Day 3**	▪ Inject Artesunate intramuscular in your child's upper leg (only applicable for cohort 3 with 5 doses of 2,4 mg/kg Artesunate)<br>▪ Take oral anti-malaria therapy (only applicable to cohorts 1 and 2)<br>▪ Take your child's temperature, heartbeat, blood pressure and breathing.<br>▪ Perform a non-invasive oto-acoustic test (only certain countries: Ghana, Gabon and Kenya)<br>▪ Take a few drops of your child's blood from the finger*<br>▪ If clinically indicated, take blood from your child's arm<br>▪ Discharge from hospital and make appointment for follow up visits
**Day 7**	▪ Take your child's temperature, heartbeat, blood pressure and breathing.<br>▪ Take a few drops of your child's blood from the finger<br>▪ If clinically indicated, take blood from your child's arm
**Day 14**	▪ Take a few drops of your child's blood from the finger<br>▪ Take your child's temperature, heartbeat, blood pressure and breathing.
**Day 28/last day**	▪ Take a few drops of your child's blood from the finger<br>▪ Take your child's temperature, heartbeat, blood pressure and breathing.<br>▪ If clinically indicated, take blood from your child's arm

*Blood smears to be taken every 6 hours for at least 48hs or until 3 consecutive negative smears.

Appendix G. Summary of Product Character

Summary of Product Character

1. Name of the finished pharmaceutical product

Artesunate for Injection

2. Qualitative and quantitative composition

Each vial of Artesunate for Injection contains 60mg Artesunate sterile powder; assembled solvent contains 1 ml 5% sodium bicarbonate solution per ampoule.

3. Pharmaceutical form

Powder injection

4. Clinical particulars

4.1 Therapeutic indications

Used in the treatment of severe malaria and emergency treatment of critical cases.

4.2 Posology and method of administration

The product can be administered intravenously or by intramuscular injection after dilution with 5% dextrose or physiological saline for injection (0.9% sodium chloride) to the anterior thigh.

Dose

2.4 mg/kg at 0, 12, and 24 hours then daily until oral treatment can be substituted.

Preparation

Inject the attached 1ml 5% Sodium Bicarbonate solution into the vial of Artesunate for Injection by syringe, shake until completely dissolved. A clear solution should be obtained (otherwise should discard the prepared solution). Insert the syringe needle in the vial to get rid of gas, dilute as directed below.

For IV injection: add approximately 5 ml of 5% glucose or physiological saline for injection (0.9% sodium chloride) to the solution in the vial to create a solution containing 10 mg Artesunate per ml (total volume is 6 ml), mixed well. Withdraw the necessary amount of Artesunate solution from the vial into a syringe and inject slowly Speed of IV: 3~4 ml/min.

For IM injection: add approximately 2 ml of 5% glucose injection or physiological saline (0.9% sodium chloride solution) to create a solution containing 20 mg of Artesunate per ml (total volume is 3 ml) , mixed well. Withdraw the necessary amount of Artesunate solution from the vial into a syringe and inject.

A clear solution should be obtained when added 5% Glucose or 0.9% Sodium Chloride injection (5ml or 2ml), otherwise should discard the prepared solution when the clear solution could not be obtained.

4.3 Contraindications

Artesunate for Injection is contraindicated in Patients with known hypersensitivity to the active ingredient.

4.4 Special warnings and special precautions for use:

Use this medicine according to the packing insert.

- Inject this product after dissolved immediately. It must not be used if opacity occurred.
- This product should not be used for intravenous drip.

4.5 Interaction with other medicinal products and other forms of interaction

No obvious injurious interaction has been reported.

4.6 Pregnancy and lactation

Rat embryo toxicity was discovered in toxicity study, using the product in organic growing period may cause embryo absorption, so early (within 3 months) pregnant women should use the drug carefully.

4.7 Effects on ability to drive and use machines

Nonexistence or can be ignored.

4.8 Undesirable effects

Blood and lymphatic systems disorders

Uncommon: Neutropenia and anaemia (both occasionally severe), thrombocytopenia

Very rare: Pure red cell aplasia

Nervous system disorders

Common: Headache, insomnia, tinnitus (with or without decrease in auditory function)

Very rare: cases of peripheral neuropathy (or paraesthesiae) have been reported

Common: Cough, nasal symptoms

Gastrointestinal disorders

Common: Nausea, vomiting, abdominal pain or cramps, diarrhoea

Rare: Rises in serum amylase. Cases of pancreatitis have been reported

Hepatobiliary disorders

Uncommon: Transient rises in liver enzymes (AST, ALT)

Rare: Hepatitis

Skin and subcutaneous tissue disorders

Common: Rash, alopecia

Musculoskeletal and connective tissue disorders

Common: Arthralgia, muscle disorders

General disorders and administration site conditions

Common: Fatigue, malaise, fever

In clinical use type 1 hypersensitivity reactions have been reported (estimated incidence 1:3000).

4.9 Overdose

Transient reticulocytopenia may occurs when dose more than 3.75mg/kg is given.

5. Pharmacological properties

5.1 Pharmacodynamic properties:

Artesunate affects the ultrastructure of rodent intraerythrocytic plasmodium, thereby changing the membrane structure of plasmodium. The substance effect on food vacuole, pellicle and mitochondria first, then nuclear membrane, endoplasmic reticulum, and nuclear chromatin as well. It indicates that the main mode of action of Artesunate is interfering the function of pellicle-mitochondria. It may be the cause that Artesunate effect on food vacuole, blocking the nutritive intake at early stage, amino acid hunger appearing fast, forming autophagic vacuole quickly and then ejected from plasmodium. So plasmodium dies of losing a large of cytoplasm. The intake results of falciparum in vitro to tritrum-labeled isoleucine also shows that the mode of action of Artesunate may he inhibiting the protein synthesis of plasmodium.

5.2 Pharmacokinetic properties

After iv. injecting, the drug serum concentration declines quickly, $T_{1/2}$ is about 30 min. The distribution of the product is very wide and the levels in intestine, liver and kidney are relatively high. The drug mainly metabolite and transform in vivo, only a little unchanged is excreted form urine and faeces. The radioimmune assay method was used to determine Artesunate in biological specimen. It's sensibility was 0.3ng/ml. i.v. injection of Artesunate to human, the drug serum level versus time curve shown to fit two compartment open model (biexponential function) decrease and the last phase $t_{1/2}$ was 38 minutes. The rate of urine drug excretion reached maximum 30-60 minute after treatment. The curve of excretion versus time was in accordance with the curve of the drug serum level versus time, both showing biexponential function decrease, but in the last phase it was slower ($t_{1/2}$=70minutes).

5.3 Preclinical safety data:

- Acute toxicity

Animals	Route of administration	LD_{50} mg/kg	
		male	female
mice	i.p.	690	700
mice	i.v.	769±69.7	
rats	i.v.	553.1±26.5	

i.v. Artesunate, the chemotherapeutic index was 792.8, and safety index is 79.9. Single i.v. for dog the highest tolerance dose is 70mg/kg , safe bound dose is 33mg/kg.

- Long-term toxicity

Dog was once per day i.v. treated by Artesunate for successive 14 days, the dose was 11.25mg/kg/day, no distinct toxicity. Dose doubled, lightly toxicosis occurs. The most sensitive index of Artesunate acute toxicosis is the reticulocytopenia.

- Special toxicology

Mutagenicity: the Ames test, V79 chromosome aberration test and murine bone marrow polychromatic erythrocyte micronucleus test, gave negative results, indicating that the drug has no mutagenic effect.
Toxicity on reproductive system: Artesunate has no influence on the reproductive function in male rats after Artesunate s.c. injetion, but if pregnant rats were s.c. injected, some embryo-toxic effects were observed.

6. Pharmaceutical particulars

6.1 List of excipients

No excipients are included in the finished products.
Ethanol is used as the solvent during the manufacturing of Artesunate Sterile Powder, so the limit of residual Ethanol in the finished product of Artesunate for Injection is 5000ppm.

6.2 Incompatibilities

In the absence of compatibility study, this pharmaceutical product must not be mixed with other pharmaceutical products.

6.3 Shelf life

3 years

6.4 Special precaution for storage

Stored below 30℃,protected from light.

6.5 Nature and contents of container

Glass vials and ampoule.

6.6 Instructions for use and handling <and disposal>

No special requirement.

7. MARKETING AUTHORISATION HOLDER

Guilin Pharmaceutical Co., Ltd.

Address: No.17 Shanghai Road, Guilin, China

Postcode：541002

Tel: 0086-773-3833116

Fax: 0086-773-3832783

Web：http://www.guilinpharma.com

8. MARKETING AUTHORISATION NUMBER(S)

Guilin Pharmaceutical Co., Ltd.

Address: No.17 Shanghai Road, Guilin, China

Postcode：541002

Tel: 0086-773-3833116

Fax: 0086-773-3832783

Web：http://www.guilinpharma.com

Manufacturing site: No.43 Qilidian Road, Guilin, China

9. DATE OF FIRST AUTHORISATION/RENEWAL OF THE AUTHORISATION

6th April 1987/ 20th February 2003

State medicine approved No. H 10930195 (China)

10. DATE OF REVISION OF THE TEXT

Date of submission: 28th June 2007

Appendix H. Investigator's Agreement: *To be added*

Appendix J. PK-Sampling Instructions

Pharmacokinetic measurements

Determination of drug concentration in biological samples

Artesunate and dihydroartemisinin will be analyzed by IKP-Stuttgart using a highly sensitive liquid chromatography-mass spectrometry (LC-MS-MS). The methods used will be referred to in the clinical study report.

Collection of blood samples

Samples of 400 µL EDTA blood will be collected and subsequently plasma separated

(approx. 200 µl) and stored at - 20°C at the study site until shipping to the analytical

laboratory. Plasma should be pipetted in tubes with a screw-cap only!

All samples need a correct labeling to avoid mix up of plasma samples. The labeling on the

tube is "tube reference number", "date" and "time of collection".

The date and time of collection and tube reference number will be recorded on the appropriate CRF.

Sample processing and shipping

Samples will be stored at ≤ -20°C and transported to the IKP-Stuttgart within 12 months of collection and must remain frozen at all times. Where possible samples should be shipped in batches and shipment should be coordinated with the receiving site to ensure that samples arrive within working hours. A requisition sheet, detailing the protocol study number, subject enrolment number and date of sample collection, should accompany the shipment.

Blood sampling time schedule

Three blood samples (ea. 400 µl) are requested from each participant.
The time points for blood withdrawal are defined in relation to treatment cohort and artesunate dosing: (Table)
Depending on the last digit of the subject number, each subject is assigned to a destinct selection of 3 out of the below listed time points.
The exact date and time point of the two blood samples will be recorded in the appropriate CRF.

Table: Blood sampling times according to artesunate regimen and dosing time

Cohorts 1 and 2:

		initial dose							24h dose							48h dose						
		Blood sampling times																				
**ID#**	t [m	5	10	30	60	120	240	360	5	10	30	60	120	240	360	5	10	30	60	120	240	360
Xxxx0		x										x						x				
Xxxx1			x										x			x						
Xxxx2				x											x		x					
Xxxx3					x									x		x						
Xxxx4						x					x						x					
Xxxx5							x			x								x				
Xxxx6								x	x											x		
Xxxx7		x											x									x
Xxxx8			x									x									x	
Xxxx9							x				x								x			

Cohort 3:

		initial dose							12h dose							24h dose							48h dose							72h dose						
		Blood sampling times																																		
**ID#**	t [min]	5	10	30	60	120	240	360	5	10	30	60	120	240	360	5	10	30	60	120	240	360	5	10	30	60	120	240	360	5	10	30	60	120	240	360
Xxxx0		x																							x							x				
Xxxx1			x																				x							x						
Xxxx2				x											x		x																			
Xxxx3					x									x		x																				
Xxxx4						x																		x							x					
Xxxx5							x			x								x																		
Xxxx6								x	x																		x									
Xxxx7													x							x																x
Xxxx8												x														x									x	
Xxxx9											x								x															x		

Population pharmacokinetics analysis

The maximum plasma concentrations (Cmax) and the time to reach maximum plasma concentration (tmax) will be determined by inspection of the artesunate and dihydroartemisinin plasma concentration-time profiles. The terminal elimination rate constant (lz) will be calculated by log-linear regression of the terminal portion of the concentration-time profiles where there are sufficient data (a minimum of 3 plasma concentration values in the terminal log-linear phase, spanning an interval of at least 2 half-lives). Terminal elimination half-life (t1/2) will be calculated as 0.693/lz. The area under the plasma concentration-time curve, up to the time of the last quantifiable plasma concentration (AUC0-t) will be calculated by the linear trapezoidal rule. AUC (0-t) will be extrapolated to infinity using lz to obtain AUC where there are sufficient data. The apparent oral clearance (CL/F) will be calculated as Dose/AUC. The actual sampling times, if different from protocol sampling times, will be used in the pharmacokinetic calculations.

A population pharmacokinetic model for artesunate when administered to children will be developed and used to compare pharmacokinetic profiles between intravenous and intramuscular administration.
The population pharmacokinetics of artesunate and dhydroartemisinin will be modeled using the non-linear mixed effects approach.(e.g. phoenixxxxx) Individual profiles of artesunate and dhydroartemisinin exposure will be generated from Bayesian post-hoc estimates of individual pharmacokinetic parameters separately for both dosing schemes. Important covariates will be considered. A statistically significant improvement in the objective function, improvement in the precision of the parameter estimate (standard error), and reduction in inter-individual and intra-individual variability determines the importance of the covariates as predictors. Multiple linear or logistic regressions, where appropriate, will be performed to model the relationship between random effects (i.e. estimates of pharmacokinetic parameters) and fixed effects (e.g. demographic, laboratory, clinical, and genetic data) as independent variables. The correlation between pharmacodynamic measures (e.g. parasite clearance) and pharmacokinetic parameters, host genotypes and covariates will be modeled using multiple linear or logistic regression analysis as appropriate.
